# Risk factors for unsuccessful tuberculosis treatment outcomes in children

**DOI:** 10.1371/journal.pone.0222776

**Published:** 2019-09-25

**Authors:** Meherunissa Hamid, Meredith B. Brooks, Falak Madhani, Hassan Ali, Mohammad Junaid Naseer, The Childhood Tuberculosis Karachi Group, Mercedes Becerra, Farhana Amanullah

**Affiliations:** 1 Global Health Directorate, The Indus Health Network, Karachi, Sindh, Pakistan; 2 Department of Global Health and Social Medicine, Harvard Medical School, Boston, MA, United States of America; 3 Department of Pediatrics, The Indus Hospital, Karachi, Sindh, Pakistan; Jamia Hamdard, INDIA

## Abstract

**Objective:**

Pakistan has a high pediatric burden of tuberculosis, but few studies describe the treatment experience of children with tuberculosis in Pakistan. We sought to identify risk factors for unsuccessful treatment outcomes in children with drug-susceptible tuberculosis identified in eight hospitals in Karachi, Pakistan.

**Design:**

We conducted a retrospective cohort study among children (<15 years old) treated with first-line anti-tuberculosis drugs for presumed or confirmed drug-susceptible tuberculosis between 2016 and 2017. We assessed risk factors for experiencing an unsuccessful treatment outcome through multivariable logistic regression analysis.

**Results:**

In total, 1,665 children initiated tuberculosis treatment, including 916 (55.0%) identified through intensified case finding. Unsuccessful treatment outcomes were experienced by 197 (11.8%) children, comprising 27 (1.6%) deaths, 16 (1.0%) treatment failures, and 154 (9.3%) lost to follow-up. An additional 47 (2.8%) children had outcomes not evaluable. In multivariable analysis, children 0–4 years old (OR: 1.80, 95% CI: 1.07–3.04), males (OR: 1.48, 95% CI: 1.04, 2.11), and those with bacteriologic confirmation of disease (OR: 3.39, 95% CI: 1.98, 5.80) had increased odds of experiencing an unsuccessful treatment outcome.

**Conclusion:**

Our findings suggest a need to deploy strategies to identify children earlier in the disease process and point to the need for interventions tailored for young children once treatment is initiated.

## Introduction

Globally, one million children (<15 years old) fall sick with tuberculosis (TB) each year.[1,2] In 2017, there were approximately 233,000 childhood TB-related deaths worldwide.[3] Children are a uniquely vulnerable population who are often under-diagnosed because they experience non-specific symptoms.[4] Conventional diagnostic tests, such as sputum smear microscopy and culture, have low sensitivity in children due to an inability to produce sputum or the presence of paucibacillary TB.[5] A lack of microbiological confirmation and capacity to clinically diagnose TB in children may contribute to missed cases, delayed or inappropriate treatment, progression of disease, and increased risk of disease and death. Poverty, overcrowding, and malnutrition further contribute to childhood TB in communities with high TB burdens.[6]

Among children who are diagnosed with TB disease and initiate treatment, some groups may be at higher risk of experiencing unsuccessful treatment outcomes, including death or being lost to follow-up. Previously identified risk factors for unsuccessful treatment outcomes in children with TB include being HIV positive[7,8], being less than five years of age[7,9], having low body weight[10], and smear positivity.[11] The role of these and other risk factors may vary by setting and population.

Pakistan is a high TB-burden country with an estimated 525,000 (373,000–704,000) TB cases in 2017, including 57,000 (51,000–63,000) children ≤ 14 years old.[3] This is likely an underestimation, however, because the most common model used for TB diagnosis and treatment in Pakistan has been passive case finding (PCF), which is known to miss a large proportion of prevalent TB cases.[12] The childhood TB program of the Zero TB Initiative in Karachi, Pakistan, deployed intensified case finding (ICF) efforts to identify and diagnose the missing children suffering from TB in the health system and start them more promptly on effective treatment.

To date, there has been limited patient-level data on pediatric TB available in Pakistan and TB outcomes are not reported, hindering the ability to describe the treatment experiences of children and to identify risk factors for unsuccessful treatment outcomes. This knowledge is essential to inform local programs to tailor their childhood TB management efforts to better serve children at risk of experiencing unsuccessful TB treatment outcomes. To address this gap, we assessed risk factors for unsuccessful treatment outcomes for children with drug-susceptible (DS-) TB in Karachi, Pakistan.

## Materials and methods

Through a thorough chart review, we identified children less than 15 years old who were initiated on first-line anti-TB treatment for presumed or confirmed DS-TB in the pediatric outpatient departments of eight public and private hospitals in Karachi, Pakistan, between July 2016 and December 2017. Children were initially identified through either PCF mechanisms—such as routine walk-in, doctor referral for TB screening, or transfer in from another clinic—or through ICF efforts. The ICF algorithm began with assigning a trained nurse to screen children using structured questions about symptoms, such as cough, fever, poor weight gain or weight loss and other risk factors, including TB contact history in the preceding 24 months. If any symptoms or risk factors were present, the child was identified as being at high risk for TB and was evaluated by a trained medical officer. After a medical history and physical exam were completed, additional tests were requested, including tuberculin skin tests, chest X-ray or other relevant imaging, and collection of a sputum specimen or gastric aspirate for an Xpert MTB/RIF assay as first line bacteriologic testing. Children were diagnosed with TB either through bacteriologic confirmation via Xpert MTB/RIF or clinically.

Children were enrolled into treatment and initiated on a TB treatment regimen per local (National TB program) guidelines[13] that are based on recommendations from the World Health Organization.[14] As part of routine care, children were followed throughout the duration of treatment until a treatment outcome was experienced, per standard definitions.[13,14] While the standard definitions are used for adult patients and children able to provide samples for bacteriologic testing, in children who are diagnosed on clinical grounds, treatment failure is defined as a lack of response to 3–5 months of compliant TB treatment, as evidenced by a persistence of symptoms, weight loss or no weight gain, and/or no change or worsening of the chest radiograph. For this analysis, we used composite treatment outcomes: successful (cure and treatment completion) or unsuccessful (death, treatment failure, or lost to follow-up). Individuals whose treatment outcome was not evaluated were excluded from analysis.

During the chart review, children’s demographic, clinical, and treatment-related characteristics were extracted from the clinical records. Demographic information included sex, age, height, weight, and the hospital where the child was enrolled for TB treatment. Children were classified into relevant categories by age group: 0–4, 5–9 and 10–14 years old. We calculated body mass index (BMI-) for-age Z-scores for each child and categorized children as malnourished if their Z-score was two or more standard deviations [SD] below the reference mean. Clinical characteristics included mechanism of diagnosis (i.e., whether the child was bacteriologically positive or was clinically diagnosed based on suggestive history, symptoms, exposure, and tests), the type of TB (pulmonary or extra-pulmonary), and whether the child was identified via PCF or ICF. Children were excluded from this analysis for any of the following reasons: if they were still on treatment at the time of the study; if drug-susceptibility test results showed resistance requiring a regimen change; or if they were found to have an illness other than TB.

We report the frequency and percentage of baseline characteristics and treatment outcomes, stratifying by PCF and ICF and using chi-squared tests to assess whether the two case-finding strategies identified different populations of children. We assess the association of covariates with an unsuccessful treatment outcome through univariate logistic regression analysis. Covariates associated with unsuccessful outcomes in univariate analysis with a p-value ≤0.20 were considered as candidates for inclusion in the final multivariable model. Covariates retained in the final model were those associated with an unsuccessful treatment outcome (p-value ≤0.05), as well as age, sex, site of TB disease, and malnourishment because of the latter’s established link with TB treatment outcomes; we also retained in the final model the type of case-finding strategy. Multiple imputation was used to account for missing values in multivariable analysis. All tests are two-sided with an alpha of 0.05. Analyses were completed in SAS version 9.4 (SAS Institute Inc., Cary, NC, USA).

### Ethics statement

This analysis used data collected programmatically for routine screening, thus no one provided informed consent, and these data were completely de-identified prior to the analysis. The Institutional Review Board (IRB) of the Indus Hospital determined the study exempt from ongoing IRB review and waived the requirement for informed consent.

## Results

A total of 1,709 children less than 15 years old initiated treatment with first-line anti-TB drugs between July 1, 2016 and December 31, 2017, in one of the eight included hospitals in Karachi, Pakistan. We excluded 36 (2.1%) children who were still on TB treatment at the time of the analysis. Another 6 (0.4%) were excluded due to a change in final diagnosis and 2 (0.1%) because their regimen was changed to include second-line drugs after receiving drug-susceptibility test results. Baseline demographic, clinical, and treatment-related characteristics of the 1,665 included children are summarized in Table 1. In all, 732 (44.0%) patients were male, and 933 (56.0%) were female. A total of 444 (26.7%) children were 0–4 years old, 644 (38.7%) were 5–9, and 575 (34.6%) were 10–14. A large proportion (565 [40.5%]) were malnourished. Only 136 (8.2%) children had a positive bacteriologic TB confirmation, of which the majority (92 [67.7%]) were aged 10–14 years old. A total of 1,252 (75.2%) children were diagnosed with pulmonary TB only, while 413 (24.8%) had extra-pulmonary involvement. ICF efforts identified 916 (55.5%) children who initiated treatment. Compared to children identified through PCF, those identified through ICF were younger (p<0.001), a larger proportion were males (p = 0.003), and fewer had bacteriologic confirmation of disease (p<0.001), extra-pulmonary involvement (p<0.001), or malnutrition (p<0.001).

**Table 1:** Baseline demographics and clinical characteristics of 1,665 children treated for drug-susceptible tuberculosis, stratified by type of case-finding strategy.

Characteristic	Variable	Total N available N = 1,665	Total, n (%)	ICF (n = 916)	PCF (n = 734)	P-value
Age	0–4 y	1,663	444 (26.7)	269 (29.4)	168 (22.9)	<0.001
	5–9 y		644 (38.7)	385 (42.1)	255 (34.8)	
	10–14 y		575 (34.6)	261 (28.5)	310 (42.3)	
Sex	Male	1,665	732 (44.0)	432 (47.2)	292 (39.8)	0.003
Bacteriologically positive	B+	1,665	136 (8.2)	38 (4.2)	97 (13.2)	<0.001
Site of TB disease	PTB	1,665	1,252 (75.2)	766 (83.6)	472 (64.3)	<0.001
Malnourished	BMI-for-age Z-score ≤2	1,396	565 (40.5)	239 (34.1)	324 (47.2)	<0.001
Type of case-finding	ICF	1,650	916 (55.5)			
	PCF		734 (44.5)			
	Walk-in		585 (35.3)			
	Referral		44 (8.7)			
	Transfer in		5 (0.3)			

Abbreviations: B+: Bacteriologically positive; BMI: Body Mass Index; ICF: Intensified Case Finding; PCF: Passive Case Finding; PTB: Pulmonary Tuberculosis; TB: Tuberculosis.

A total of 1,421 (85.4%) children experienced successful treatment outcomes while 197 (11.8%) had unsuccessful outcomes. The latter comprised 27 (1.6%) children who died, 16 (1.0%) children in whom treatment failed, and 154 (9.3%) children were lost to follow-up. Another 47 (2.8%) children were not evaluable (Table 2). While the overall frequency of unsuccessful treatment outcomes is similar for children identified through ICF and PCF (p = 0.138), more children identified through PCF either died (p = <0.001) or had treatment fail (p = 0.003).

**Table 2:** Tuberculosis treatment outcomes among children in Karachi, Pakistan, stratified by type of case-finding strategy.

	Total (n = 1,665)	ICF (n = 916)	PCF (n = 734)	P-value
**Successful**	**1,421 (85.4)**	**787 (85.9)**	**621 (84.6)**	**0.138**
Cure	38 (2.3)	16 (1.8)	22 (3.0)	0.092
Treatment complete	1383 (83.1)	771 (84.2)	599 (81.6)	0.168
**Unsuccessful**	**197 (11.8)**	**98 (10.7)**	**97 (13.2)**	**0.138**
Died	27 (1.6)	6 (0.7)	21 (2.9)	<0.001
Treatment failure	16 (1.0)	3 (0.3)	13 (1.8)	0.003
Lost to follow-up	154 (9.3)	89 (9.7)	63 (8.6)	0.429
**Not evaluated**	**47 (2.8)**	**31 (3.4)**	**16 (2.2)**	**0.144**

Abbreviations: ICF: Intensified Case Finding; PCF: Passive Case Finding.

In univariate analysis, the odds of having an unsuccessful treatment outcome was 2.5 times higher in children who had bacteriologic confirmation compared to children who were clinically diagnosed (p<0.001) (Table 3). In multivariate analysis—controlling for malnourishment, site of TB disease, and type of case-finding strategy—children 0–4 years old, males, and those with bacteriologic confirmation of disease had increased odds of an unsuccessful treatment outcome compared to children 10–14 years old (OR: 1.80, 95% CI: 1.07, 3.04; p = 0.028), females (OR: 1.48, 95% CI: 1.04, 2.11; p = 0.030) and those who were clinically diagnosed (OR: 3.39, 95% CI: 1.98, 5.80; p<0.001), respectively.

**Table 3:** Univariate and multivariable associations of child characteristics and unsuccessful treatment outcomes.

Characteristic	Variable	Univariate OR (95% CI)	p-value	Multivariable OR (95% CI)	p-value
Age	0–4 y	1.35 (0.92, 1.97)	0.135	1.80 (1.07, 3.04)	0.028
	5–9 y	1.11 (0.77, 1.58)	0.754	1.03 (0.64, 1.67)	0.900
	10–14 y	REF	REF	REF	REF
Sex	Male	1.34 (1.00, 1.81)	0.052	1.48 (1.04, 2.11)	0.030
Bacteriologically positive	B+	2.49 (1.62, 3.85)	<0.001	3.39 (1.98, 5.80)	<0.001
Site of TB disease	PTB	1.17 (0.84, 1.64)	0.362	1.33 (0.88, 1.99)	0.174
Malnourished	BMI-for-age Z-score ≤2	1.28 (0.91, 1.79)	0.157	1.35 (0.90, 2.03)	0.143
Type of case-finding	ICF	0.80 (0.59, 1.08)	0.138	0.87 (0.60, 1.26)	0.469
	PCF	REF	REF	REF	REF

Abbreviations: B+: Bacteriologically positive; BMI: Body Mass Index; CI: Confidence Interval; ICF: Intensified Case Finding; OR: Odds Ratio; PCF: Passive Case Finding; PTB: Pulmonary Tuberculosis; TB: Tuberculosis

## Discussion

Unsuccessful TB treatment outcomes were experienced by fifteen percent of children younger than 15 years old undergoing DS-TB treatment in Karachi, Pakistan. The majority (78%) of those unsuccessful outcomes were lost to follow-up. Younger age (0–4 years), male sex, and having bacteriologic confirmation of disease were risk factors for unsuccessful treatment outcomes.

Unsuccessful outcomes among children in Karachi were experienced more frequently compared to a recent study of child TB outcomes from Sindh, Pakistan, by Laghari et al.[11], despite consisting of children in the same age range with a similar breakdown by sex, age, and proportion with pulmonary TB. Major differences between the two cohorts include that, in the Laghari et al.[11] study, almost half of the study population were from a rural district and over ninety percent of children less than five years of age experienced a successful treatment outcome. In contrast, our study includes only an urban population and eighty-three percent of children less than five years old experienced a successful treatment outcome; the majority of unsuccessful outcomes for this age group were lost to follow-up. Another important difference to note is that the most of our cohort was found through ICF activities. These activities, identified the majority of children under five years old who were lost to follow-up and who otherwise might not have been diagnosed.

Consistent with our observations, Laghari et al.[11] also found that children with bacteriologically confirmed smear positive pulmonary TB were at increased risk of unsuccessful treatment outcomes. While our cohort had a smaller proportion of children with bacteriologically confirmed TB compared to other studies in Pakistan[11] and globally[15,16], that group had a three-fold increased odds of experiencing an unsuccessful outcome. These findings are not surprising, as children are often unable to produce sputum to undergo conventional bacteriological testing for disease confirmation.[5] Thus, children who are able to provide a sputum specimen and undergo bacteriological testing are often either older[17] or have more severe TB disease; the latter is associated with worse outcomes.

We also found that young children less than five years of age had an increased risk of unsuccessful treatment outcomes compared to older children, which is consistent with findings from other studies.[7,9,18] TB diagnosis in young children is often delayed due to the presentation of non-specific symptoms, a need to rule out other pathogens[4], and the inability of young children to produce a sputum specimen to confirm the presence of TB disease.[5] Waiting for a clinical diagnosis may have delayed treatment initiation and, ultimately, led to disease progression to a more severe state.

There were more female children with TB than male children in our cohort, which is consistent with other pediatric treatment cohorts that report even larger gender gaps.[11] Gender was a found to be a risk factor for unsuccessful treatment outcomes in this cohort and there were observed differences between males and females across several covariates—age, percentage of bacteriologically confirmed cases, site of TB disease, and malnourishment. It is unclear whether there is a larger amount of female children with TB due to biological factors or other factors, such as gender inequality in access to food, health care, and education. It is also possible that parents were less likely to bring their male child to the health center, so they were identified later in their disease progression through ICF efforts. Their more severe disease stage at diagnosis may have led to the association with experiencing unsuccessful treatment outcomes.

Although ICF itself was not associated with unsuccessful treatment outcomes, ICF identified many young children who experienced an unsuccessful outcome. Our ICF intervention was designed to find children earlier in their disease progression and who might otherwise not have been diagnosed and treated for TB.

Our study has several limitations. There was a large amount of missing data for some variables, such as malnutrition, which we addressed through use of multiple imputation methods. Only malnutrition was included as a comorbidity. Malnutrition is the major comorbidity observed in this population. Other comorbidities, such as diabetes, hepatitis, immunodeficiency, and chronic lung diseases are anticipated to be extremely low in this population and HIV testing was not consistently done. Additionally, due to the retrospective nature of the chart review and the programmatic nature of data collection, information on other patient-level characteristics that may be associated with poor treatment outcomes were not routinely assessed for or recorded. We were also unable to include social factors, such as financial status of the household or education level of the parent due to them not being recorded in patient charts. The comorbidities and social factors may have had direct or indirect impact on the child’s health or care-seeking behavior of the parents that contributed to the child experiencing unsuccessful treatment outcomes. Specific reasons for deaths were unable to be ascertained for all children, as they were not standardly recorded as part of routine practice. If death was due to factors unrelated to TB treatment, the associations observed may be overestimated.

Our results can inform efforts to improve detection and treatment of children with TB in Karachi and similar large Asian cities. They suggest that programs need to deploy strategies to identify children earlier in the disease process. Such strategies may include active case finding efforts, such as screening the household contacts of individuals diagnosed with TB, community-based interventions to screen for TB in the community or in areas where individuals may be at high risk of TB disease, and education campaigns to increase knowledge on signs, symptoms, and risks of TB disease. Our results also suggest that young children may require tailored interventions once treatment is initiated.

## Supporting information

S1 File Data set. (PDF)
