# Supplementary material for: Risk factors for unsuccessful tuberculosis treatment outcomes in children
Source: PLoS One. 2019 Sep 25;14(9):e0222776. doi: 10.1371/journal.pone.0222776 (PMC6760830; doi:10.1371/journal.pone.0222776)
Supplement: S1 File — (PDF) [file pone.0222776.s001.pdf]

#	Site_Name	Age_month	Sex	weight	Height	Patient_Source	TB_type	Regimen	Diagnosis_type	Treatment_Outcome
1	Indus Hospital	60	M	14	100	Walk-In	PTB	CAT-1	Clinical	Treatment Complete
2	Indus Hospital	144	F	19	125	Walk-In	EPTB	CAT-1	Clinical	Treatment Complete
3	Indus Hospital	156	F	25	125	Walk-In	PTB	CAT-1	Clinical	Treatment Complete
4	Indus Hospital	156	F	30	150	Walk-In	EPTB	CAT-1	Clinical	Treatment Complete
5	Indus Hospital	72	F	12	107	Walk-In	EPTB	CAT-1	Clinical	Treatment Complete
6	Indus Hospital	60	M	10	N/A	Walk-In	PTB	CAT-1	B+	Lost to follow up
7	Indus Hospital	156	F	29	138	Walk-In	EPTB	CAT-1	Clinical	Treatment Complete
8	Indus Hospital	156	M	31	150	Walk-In	PTB	CAT-1	Clinical	Treatment Complete
9	Indus Hospital	144	F	25	140	Walk-In	EPTB	CAT-1	Clinical	Treatment Complete
10	Indus Hospital	168	M	9	75	Walk-In	PTB	CAT-1	Clinical	Treatment Complete
11	Indus Hospital	12	M	6	70	Walk-In	PTB	CAT-1	Clinical	Treatment Complete
12	Indus Hospital	72	F	45	160	Walk-In	PTB	CAT-1	Clinical	Treatment Complete
13	Indus Hospital	120	M	18	117	Walk-In	EPTB	CAT-1	Clinical	Treatment Complete
14	Indus Hospital	144	F	15	126	Walk-In	PTB	CAT-1	B+	Cure
15	Indus Hospital	132	F	20	133	Walk-In	PTB	CAT-1	Clinical	Treatment Complete
16	Indus Hospital	84	F	20	120	Walk-In	PTB,EPTB	CAT-1	Clinical	Treatment Complete
17	Indus Hospital	144	F	24	135	Walk-In	PTB	CAT-1	B+	Cure
18	Indus Hospital	72	F	15	110	Walk-In	PTB	CAT-1	Clinical	Treatment Complete
19	Indus Hospital	168	F	27	160	Walk-In	PTB	CAT-1	B+	Failure
20	Indus Hospital	144	M	21	135	Walk-In	PTB	CAT-1	Clinical	Treatment Complete
21	Indus Hospital	84	F	14	113	Walk-In	PTB	CAT-1	B+	Cure
22	Indus Hospital	120	F	7	N/A	Walk-In	PTB	CAT-1	B+	Died
23	Indus Hospital	120	M	21	115	Walk-In	PTB	CAT-1	Clinical	Treatment Complete
24	Indus Hospital	144	F	23	150	Walk-In	PTB	CAT-2	B+	Transfer out
25	Indus Hospital	132	M	20	115	Walk-In	PTB	CAT-1	Clinical	Treatment Complete
26	Indus Hospital	12	M	8	76	Walk-In	PTB	CAT-1	Clinical	Treatment Complete
27	Indus Hospital	144	F	17	130	Walk-In	PTB	CAT-1	Clinical	Treatment Complete
28	Indus Hospital	24	M	9	77	Walk-In	PTB	CAT-1	Clinical	Treatment Complete
29	Indus Hospital	84	M	18	118	Walk-In	EPTB	CAT-1	Clinical	Treatment Complete
30	Indus Hospital	132	F	27	133	Walk-In	EPTB	CAT-1	Clinical	Treatment Complete
31	Indus Hospital	96	F	15	110	Walk-In	PTB	CAT-1	Clinical	Treatment Complete
32	Indus Hospital	36	F	15	95	Walk-In	PTB	CAT-1	B+	Move to second line
33	Indus Hospital	96	M	17	120	Walk-In	EPTB	CAT-1	Clinical	Treatment Complete
34	Indus Hospital	156	F	32	N	Walk-In	EPTB	CAT-1	Clinical	Died
35	Indus Hospital	156	F	38	155	Walk-In	PTB	CAT-2	B+	Treatment Complete
36	Indus Hospital	156	F	8	N/A	Walk-In	PTB	N/A	B+	Failure
37	Indus Hospital	48	M	11	92	Walk-In	PTB	CAT-1	Clinical	Treatment Complete
38	Indus Hospital	96	F	19	122	Walk-In	PTB	CAT-2	Clinical	Diagnosis Changed
39	Indus Hospital	120	F	11	33	Walk-In	PTB	CAT-1	Clinical	Treatment Complete
40	Indus Hospital	12	M	10	75	Walk-In	PTB	CAT-1	Clinical	Treatment Complete

41	Indus Hospital	12	M	6	75	Walk-In	PTB	CAT-1	Clinical	Treatment Complete
42	Indus Hospital	24	M	7	79	Walk-In	PTB	CAT-1	Clinical	Treatment Complete
43	Indus Hospital	168	F	33	152	Walk-In	PTB	CAT-1	B+	Treatment Complete
44	Indus Hospital	96	F	16	115	Walk-In	EPTB	CAT-1	Clinical	Treatment Complete
45	Indus Hospital	48	M	12	93	Walk-In	PTB	CAT-1	Clinical	Treatment Complete
46	Indus Hospital	168	M	32	150	Walk-In	EPTB	CAT-1	Clinical	Treatment Complete
47	Indus Hospital	96	F	19	127	Walk-In	EPTB	CAT-1	Clinical	Treatment Complete
48	Indus Hospital	120	M	27	138	Walk-In	PTB	CAT-1	B+	Treatment Complete
49	Indus Hospital	12	F	7	71	Walk-In	PTB	CAT-1	Clinical	Treatment Complete
50	Indus Hospital	72	F	13	105	Walk-In	PTB	CAT-2	Clinical	Treatment Complete
51	Indus Hospital	120	F	22	133	Walk-In	PTB	CAT-1	B+	Cure
52	Indus Hospital	48	M	14	103	Walk-In	EPTB	CAT-1	Clinical	Treatment Complete
53	Indus Hospital	156	F	28	147	Walk-In	PTB,EPTB	CAT-1	Clinical	Treatment Complete
54	Indus Hospital	168	F	33	146	Walk-In	PTB	CAT-1	B+	Transfer out
55	Indus Hospital	72	F	14	105	Walk-In	PTB	CAT-1	Clinical	Treatment Complete
56	Indus Hospital	120	M	27	138	Walk-In	PTB	CAT-1	B+	Lost to follow up
57	Indus Hospital	96	F	16	123	Walk-In	PTB	CAT-1	Clinical	Treatment Complete
58	Indus Hospital	48	M	10	84	Walk-In	PTB,EPTB	CAT-1	Clinical	Died
59	Indus Hospital	168	F	31	148	Walk-In	PTB	CAT-1	B+	Cure
60	Indus Hospital	132	M	20	133	Walk-In	PTB	CAT-1	Clinical	Treatment Complete
61	Indus Hospital	96	F	17	118	Walk-In	PTB	CAT-1	Clinical	Treatment Complete
62	Indus Hospital	144	F	25	137	Walk-In	EPTB	CAT-1	Clinical	Treatment Complete
63	Indus Hospital	108	M	23	134	Walk-In	EPTB	CAT-1	Clinical	Treatment Complete
64	Indus Hospital	120	F	24	115	Walk-In	PTB	CAT-1	Clinical	Treatment Complete
65	Indus Hospital	36	M	11	86	Walk-In	PTB	CAT-1	Clinical	Lost to follow up
66	Indus Hospital	48	M	14	98	Walk-In	PTB	CAT-1	Clinical	Treatment Complete
67	Indus Hospital	12	M	7	73.5	Walk-In	PTB	CAT-1	Clinical	Treatment Complete
68	Indus Hospital	168	F	29	145	Walk-In	EPTB	CAT-1	Clinical	Treatment Complete
69	Indus Hospital	96	F	18	120	Walk-In	PTB	CAT-1	Clinical	Treatment Complete
70	Indus Hospital	96	F	17	110	Walk-In	PTB	CAT-1	Clinical	Treatment Complete
71	Indus Hospital	36	M	10	83	Walk-In	PTB	CAT-1	Clinical	Treatment Complete
72	Indus Hospital	144	F	23	133	Walk-In	EPTB	CAT-1	Clinical	Treatment Complete
73	Indus Hospital	84	F	16	110	Walk-In	PTB	CAT-1	Clinical	Treatment Complete
74	Indus Hospital	96	M	19	115	Walk-In	PTB	CAT-1	Clinical	Treatment Complete
75	Indus Hospital	144	F	37	147	Walk-In	PTB	CAT-1	B+	Treatment Complete
76	Indus Hospital	84	M	16	107	Walk-In	PTB	CAT-1	Clinical	Treatment Complete
77	Indus Hospital	36	F	10	85	Walk-In	PTB	CAT-1	Clinical	Treatment Complete
78	Indus Hospital	156	F	16	115	Walk-In	EPTB	CAT-1	Clinical	Died
79	Indus Hospital	120	M	7	74	Walk-In	EPTB	CAT-1	Clinical	Treatment Complete
80	Indus Hospital	144	F	38	160	Walk-In	PTB	CAT-1	B+	Treatment Complete
81	Indus Hospital	12	F	10.5	83	Walk-In	EPTB	CAT-1	Clinical	Treatment Complete

82	Indus Hospital	96	F	21	114	Walk-In	EPTB	CAT-1	Clinical	Treatment Complete
83	Indus Hospital	36	F	11.5	85	Walk-In	PTB	CAT-1	Clinical	Treatment Complete
84	Indus Hospital	24	M	10	78	Walk-In	PTB,EPTB	CAT-1	Clinical	Treatment Complete
85	Indus Hospital	120	F	34	150	Walk-In	PTB	CAT-1	Clinical	Treatment Complete
86	Indus Hospital	120	F	25	130	Walk-In	EPTB	CAT-1	Clinical	Treatment Complete
87	Indus Hospital	144	M	29	146	Walk-In	PTB	CAT-1	Clinical	Lost to follow up
88	Indus Hospital	108	M	20	118	Walk-In	EPTB	CAT-2	Clinical	Treatment Complete
89	Indus Hospital	132	M	20	130	Walk-In	PTB	CAT-1	Clinical	Died
90	Indus Hospital	24	F	9	76	Walk-In	PTB	CAT-1	Clinical	Treatment Complete
91	Indus Hospital	96	F	21	130	Walk-In	PTB	CAT-1	Clinical	Treatment Complete
92	Indus Hospital	168	F	28	140	Walk-In	EPTB	CAT-1	Clinical	Treatment Complete
93	Indus Hospital	84	M	16	115	Walk-In	PTB	CAT-1	Clinical	Treatment Complete
94	Indus Hospital	144	F	29	148	Walk-In	PTB	CAT-1	Clinical	Treatment Complete
95	Indus Hospital	48	F	10	90	Walk-In	PTB,EPTB	CAT-1	Clinical	Treatment Complete
96	Indus Hospital	156	F	35	158	Walk-In	PTB	CAT-1	B+	Treatment Complete
97	Indus Hospital	156	F	20	120	Walk-In	EPTB	CAT-1	Clinical	Treatment Complete
98	Indus Hospital	96	M	19	120	Walk-In	EPTB	CAT-1	Clinical	Treatment Complete
99	Indus Hospital	48	M	15	100	Walk-In	PTB	CAT-1	Clinical	Treatment Complete
100	Indus Hospital	48	M	9	85	Screening	PTB	CAT-1	Clinical	Lost to follow up
101	Indus Hospital	24	F	8	78	Screening	PTB	CAT-1	Clinical	Treatment Complete
102	Indus Hospital	156	F	45.5	158	Walk-In	PTB	CAT-1	B+	Treatment Complete
103	Indus Hospital	84	F	15	107	Walk-In	EPTB	CAT-1	Clinical	Treatment Complete
104	Indus Hospital	72	M	12	99	Walk-In	EPTB	CAT-1	Clinical	Treatment Complete
105	Indus Hospital	48	F	11	90	Walk-In	EPTB	CAT-1	Clinical	Treatment Complete
106	Indus Hospital	12	M	8	78	Walk-In	PTB	CAT-1	Clinical	Treatment Complete
107	Indus Hospital	156	M	28	143	Walk-In	PTB	CAT-2	Clinical	Treatment Complete
108	Indus Hospital	48	F	9	85	Walk-In	PTB	CAT-1	Clinical	Treatment Complete
109	Indus Hospital	36	F	10	88	Walk-In	PTB	CAT-1	Clinical	Treatment Complete
110	Indus Hospital	96	F	17	120	Walk-In	PTB	CAT-1	B+	Cure
111	Indus Hospital	84	F	12	95	Screening	PTB	CAT-1	Clinical	transfer out
112	Indus Hospital	72	F	11	100	Walk-In	EPTB	CAT-1	Clinical	Treatment Complete
113	Indus Hospital	48	F	4	64	Walk-In	PTB	CAT-1	Clinical	Diagnosis Changed
114	Indus Hospital	48	F	41	157	Walk-In	PTB	CAT-1	Clinical	Treatment Complete
115	Indus Hospital	144	M	20	135	Walk-In	PTB	CAT-1	Clinical	Treatment Complete
116	Indus Hospital	132	M	27	138	Walk-In	PTB	CAT-1	Clinical	Lost to follow up
117	Indus Hospital	108	F	17	118	Walk-In	PTB	CAT-1	B+	Cure
118	Indus Hospital	96	F	17	114	Walk-In	PTB	CAT-1	Clinical	Treatment Complete
119	Indus Hospital	144	M	27.5	147	Walk-In	EPTB	CAT-1	Clinical	Treatment Complete
120	Indus Hospital	72	F	18.5	115	Walk-In	EPTB	CAT-1	Clinical	Treatment Complete
121	Indus Hospital	168	F	26.5	149	Walk-In	PTB	CAT-1	B+	Cure
122	Indus Hospital	144	F	26.5	135	Screening	PTB	CAT-1	B+	Treatment Complete

123	Indus Hospital	108	F	15	113	Screening	PTB	CAT-1	Clinical	Transfer out
124	Indus Hospital	156	F	28	146	Walk-In	PTB	CAT-1	B+	Move to second line
125	Indus Hospital	24	F	8	78	Walk-In	PTB	CAT-1	Clinical	Diagnosis Changed
126	Indus Hospital	12	F	7.5	72	Walk-In	PTB	CAT-1	Clinical	Treatment Complete
127	Indus Hospital	108	M	25.5	137	Walk-In	PTB	CAT-1	Clinical	Treatment Complete
128	Indus Hospital	156	F	34	151	Walk-In	EPTB	CAT-1	Clinical	Treatment Complete
129	Indus Hospital	96	F	26.5	141	Walk-In	PTB,EPTB	CAT-1	Clinical	Treatment Complete
130	Indus Hospital	156	F	28	135	Walk-In	PTB	CAT-1	Clinical	Treatment Complete
131	Indus Hospital	84	F	15.5	120	Walk-In	EPTB	CAT-1	Clinical	Treatment Complete
132	Indus Hospital	144	F	35.5	146	Walk-In	PTB	CAT-1	Clinical	Treatment Complete
133	Indus Hospital	120	F	21	131	Walk-In	PTB	CAT-1	Clinical	Treatment Complete
134	Indus Hospital	144	F	38	158	Walk-In	PTB	CAT-1	Clinical	Treatment Complete
135	Indus Hospital	156	F	28	137	Walk-In	PTB	CAT-1	B+	Treatment Complete
136	Indus Hospital	120	M	15	120	Screening	PTB	CAT-1	Clinical	Treatment Complete
137	Indus Hospital	84	M	12	99	Walk-In	PTB	CAT-1	Clinical	Treatment Complete
138	Indus Hospital	36	F	10	83	Walk-In	PTB	CAT-2	Clinical	Treatment Complete
139	Indus Hospital	24	F	9	85	Screening	PTB	CAT-1	Clinical	Treatment Complete
140	Indus Hospital	144	M	24	127	Screening	PTB	CAT-1	Clinical	Treatment Complete
141	Indus Hospital	132	M	26	141	Walk-In	EPTB	CAT-1	Clinical	Treatment Complete
142	Indus Hospital	72	M	11	100	Walk-In	EPTB	CAT-1	Clinical	Died
143	Indus Hospital	60	M	15	100	Walk-In	EPTB	CAT-1	Clinical	Treatment Complete
144	Indus Hospital	96	F	16	107	Walk-In	PTB	CAT-1	B+	Lost to follow up
145	Indus Hospital	24	F	8	78	Screening	PTB,EPTB	CAT-1	Clinical	Died
146	Indus Hospital	84	M	18	106	Walk-In	EPTB	CAT-1	Clinical	Treatment Complete
147	Indus Hospital	96	F	14.5	115	Screening	PTB	CAT-1	Clinical	Treatment Complete
148	Indus Hospital	168	F	31	146	Walk-In	PTB	CAT-1	B+	Transfer out
149	Indus Hospital	12	F	8	74	Walk-In	PTB	CAT-1	Clinical	Lost to follow up
150	Indus Hospital	48	M	13	100	Screening	PTB	CAT-1	Clinical	Treatment Complete
151	Indus Hospital	12	M	8	75	Walk-In	PTB	CAT-1	Clinical	Treatment Complete
152	Indus Hospital	12	M	10	86	Walk-In	PTB,EPTB	CAT-1	Clinical	Treatment Complete
153	Indus Hospital	132	F	36	138	Walk-In	EPTB	CAT-1	Clinical	Transfer out
154	Indus Hospital	120	F	23	125	Walk-In	EPTB	CAT-1	Clinical	Treatment Complete
155	Indus Hospital	48	F	7	69	Screening	PTB	CAT-1	Clinical	Transfer out
156	Indus Hospital	84	M	17.5	113	Walk-In	PTB	CAT-1	Clinical	Treatment Complete
157	Indus Hospital	84	M	14	102	Screening	PTB	CAT-1	Clinical	Treatment Complete
158	Indus Hospital	144	F	22	129	Walk-In	EPTB	CAT-1	Clinical	Treatment Complete
159	Indus Hospital	144	F	38	154	Walk-In	PTB	CAT-1	Clinical	Treatment Complete
160	Indus Hospital	72	F	14	105	Walk-In	EPTB	CAT-1	Clinical	Treatment Complete
161	Indus Hospital	96	F	18	113	Walk-In	PTB	CAT-1	Clinical	Treatment Complete
162	Indus Hospital	132	M	20	126	Walk-In	PTB	CAT-2	Clinical	Treatment Complete
163	Indus Hospital	84	F	20	114	Walk-In	PTB,EPTB	CAT-1	Clinical	Treatment Complete

164	Indus Hospital	48	M	18	102	Walk-In	PTB	CAT-1	Clinical	Treatment Complete
165	Indus Hospital	156	M	25	130	Walk-In	EPTB	CAT-1	Clinical	Treatment Complete
166	Indus Hospital	144	F	31.5	141	Walk-In	EPTB	CAT-1	Clinical	Treatment Complete
167	Indus Hospital	168	F	33	155	Walk-In	PTB	CAT-1	Clinical	Treatment Complete
168	Indus Hospital	96	F	18	118	Walk-In	PTB	CAT-1	Clinical	Treatment Complete
169	Indus Hospital	120	F	28.5	146	Screening	EPTB	CAT-1	Clinical	Treatment Complete
170	Indus Hospital	36	F	6	74	Walk-In	PTB	CAT-1	Clinical	Treatment Complete
171	Indus Hospital	120	F	23	132	Walk-In	PTB	CAT-1	Clinical	Treatment Complete
172	Indus Hospital	84	M	17	112	Walk-In	PTB	CAT-1	Clinical	Treatment Complete
173	Indus Hospital	12	M	13	86	Walk-In	PTB	CAT-1	Clinical	Treatment Complete
174	Indus Hospital	120	F	19	120	Walk-In	PTB	CAT-1	Clinical	Treatment Complete
175	Indus Hospital	48	M	8.5	85	Screening	PTB	CAT-1	Clinical	Treatment Complete
176	Indus Hospital	48	M	17	118	Walk-In	PTB	CAT-1	Clinical	Lost to follow up
177	Indus Hospital	48	M	8.5	70	Walk-In	PTB	CAT-1	Clinical	Treatment Complete
178	Indus Hospital	120	M	22.5	124	Walk-In	EPTB	CAT-1	Clinical	Treatment Complete
179	Indus Hospital	12	M	9.5	80	Walk-In	EPTB	CAT-1	Clinical	Treatment Complete
180	Indus Hospital	168	F	24	147	Walk-In	PTB	CAT-1	Clinical	Treatment Complete
181	Indus Hospital	12	M	8.5	75	Walk-In	EPTB	CAT-1	Clinical	Treatment Complete
182	Indus Hospital	108	F	22.5	130	Walk-In	EPTB	CAT-1	Clinical	Treatment Complete
183	Indus Hospital	12	M	7	76.5	Walk-In	PTB	CAT-1	Clinical	Lost to follow up
184	Indus Hospital	156	F	29	143	Walk-In	PTB	CAT-1	B+	Cure
185	Indus Hospital	168	M	29	150	Walk-In	EPTB	CAT-1	Clinical	Diagnosis Changed
186	Indus Hospital	156	F	19	128	Walk-In	EPTB	CAT-1	Clinical	Treatment Complete
187	Indus Hospital	144	M	29	142	Walk-In	PTB	CAT-1	Clinical	Died
188	Indus Hospital	132	F	19.5	131	Walk-In	PTB	CAT-1	B+	Treatment Complete
189	Indus Hospital	96	M	19	120	Screening	EPTB	CAT-1	Clinical	Treatment Complete
190	Indus Hospital	96	F	13.5	110	Walk-In	EPTB	CAT-1	Clinical	Treatment Complete
191	Indus Hospital	120	F	11.5	115	Screening	PTB	CAT-1	Clinical	Treatment Complete
192	Indus Hospital	132	F	30	143	Walk-In	EPTB	CAT-1	Clinical	Treatment Complete
193	Indus Hospital	132	M	23	133	Walk-In	EPTB	CAT-1	Clinical	Treatment Complete
194	Indus Hospital	12	M	8	74	Walk-In	PTB	CAT-1	Clinical	Treatment Complete
195	Indus Hospital	120	M	15	115	Walk-In	PTB	CAT-1	Clinical	Treatment Complete
196	Indus Hospital	108	F	18	119	Screening	PTB	CAT-1	Clinical	Diagnosis Changed
197	Indus Hospital	60	M	8	72	Screening	PTB	CAT-1	Clinical	Treatment Complete
198	Indus Hospital	120	M	23	135	Walk-In	PTB	CAT-1	Clinical	Treatment Complete
199	Indus Hospital	36	M	9.5	84	Walk-In	EPTB	CAT-1	Clinical	Diagnosis Changed
200	Indus Hospital	132	F	21	138	Walk-In	EPTB	CAT-1	Clinical	Treatment Complete
201	Indus Hospital	48	M	15	103	Walk-In	EPTB	CAT-2	Clinical	Treatment Complete
202	Indus Hospital	48	M	11.5	90	Walk-In	PTB	CAT-1	Clinical	Lost to follow up
203	Indus Hospital	144	M	23	135	Walk-In	EPTB	CAT-1	Clinical	Treatment Complete
204	Indus Hospital	48	M	13.5	100	Walk-In	EPTB	CAT-1	Clinical	Treatment Complete

205	Indus Hospital	96	M	16.5	113	Walk-In	EPTB	CAT-1	Clinical	Lost to follow up
206	Indus Hospital	132	F	19.5	124	Walk-In	PTB	CAT-1	Clinical	Treatment Complete
207	Indus Hospital	12	M	10	78	Walk-In	PTB	CAT-2	Clinical	Treatment Complete
208	Indus Hospital	156	F	22	148	Walk-In	PTB	CAT-1	Clinical	Treatment Complete
209	Indus Hospital	156	F	34	153	Walk-In	EPTB	CAT-1	Clinical	Treatment Complete
210	Indus Hospital	156	F	32.5	142	Walk-In	PTB	CAT-1	Clinical	Treatment Complete
211	Indus Hospital	84	F	14	107	Walk-In	PTB	CAT-1	Clinical	Died
212	Indus Hospital	96	F	5	60	Walk-In	PTB	CAT-1	Clinical	Treatment Complete
213	Indus Hospital	24	M	8	77	Walk-In	PTB	CAT-1	Clinical	Treatment Complete
214	Indus Hospital	24	F	9.5	82	Walk-In	PTB	CAT-1	Clinical	Treatment Complete
215	Indus Hospital	60	M	15	102	Walk-In	PTB	CAT-1	Clinical	Treatment Complete
216	Indus Hospital	144	F	31	145	Walk-In	PTB	CAT-1	Clinical	Lost to follow up
217	Indus Hospital	168	F	30	150	Walk-In	PTB	CAT-2	Clinical	Treatment Complete
218	Indus Hospital	72	M	19	112	Walk-In	PTB	CAT-1	Clinical	Treatment Complete
219	Indus Hospital	48	F	10.5	95	Walk-In	PTB	CAT-1	Clinical	Treatment Complete
220	Indus Hospital	84	F	21.5	123	Walk-In	PTB	CAT-1	Clinical	Treatment Complete
221	Indus Hospital	24	M	7	74	Walk-In	PTB	CAT-1	Clinical	Lost to follow up
222	Indus Hospital	96	F	17	115	Walk-In	PTB	CAT-1	Clinical	Treatment Complete
223	Indus Hospital	84	F	23	120	Walk-In	PTB	CAT-1	Clinical	Treatment Complete
224	Indus Hospital	132	M	23	138	Walk-In	PTB	CAT-1	B+	Cure
225	Indus Hospital	36	F	9.5	89	Screening	PTB	CAT-1	Clinical	Transfer out
226	Indus Hospital	72	F	16	109	Walk-In	PTB	CAT-1	Clinical	Treatment Complete
227	Indus Hospital	60	F	14	101	Walk-In	PTB	CAT-2	Clinical	Treatment Complete
228	Indus Hospital	12	M	5.5	67	Screening	PTB	CAT-1	Clinical	Lost to follow up
229	Indus Hospital	144	F	31	153	Walk-In	EPTB	CAT-1	Clinical	Treatment Complete
230	Indus Hospital	156	F	31	147	Walk-In	PTB	CAT-1	Clinical	Treatment Complete
231	Indus Hospital	84	M	11	87	Walk-In	EPTB	CAT-1	Clinical	Treatment Complete
232	Indus Hospital	72	F	10.5	95	Walk-In	EPTB	CAT-1	Clinical	Treatment Complete
233	Indus Hospital	108	M	15.5	111	Screening	PTB	CAT-1	Clinical	Lost to follow up
234	Indus Hospital	12	M	7.5	72	Screening	PTB	CAT-1	Clinical	Treatment Complete
235	Indus Hospital	48	M	10.5	91	Walk-In	PTB	CAT-1	Clinical	Treatment Complete
236	Indus Hospital	96	F	13	103	Walk-In	PTB	CAT-2	Clinical	Treatment Complete
237	Indus Hospital	84	F	7.5	68	Walk-In	PTB	CAT-1	Clinical	Transfer out
238	Indus Hospital	156	F	27	138	Walk-In	PTB	CAT-1	B+	Treatment Complete
239	Indus Hospital	48	F	8	93	Screening	PTB	CAT-1	Clinical	Treatment Complete
240	Indus Hospital	72	M	19	118	Screening	EPTB	CAT-1	Clinical	Treatment Complete
241	Indus Hospital	120	M	22	123	Screening	EPTB	CAT-1	Clinical	Treatment Complete
242	Indus Hospital	60	M	16	105	Walk-In	PTB	CAT-1	Clinical	Treatment Complete
243	Indus Hospital	36	M	12.5	87	Walk-In	PTB	CAT-1	Clinical	Treatment Complete
244	Indus Hospital	120	F	20	128	Walk-In	PTB	CAT-1	B+	Cure
245	Indus Hospital	84	F	13	105	Walk-In	PTB	CAT-1	Clinical	Treatment Complete

246	Indus Hospital	36	F	10.5	90	Walk-In	PTB,EPTB	CAT-1	Clinical	Lost to follow up
247	Indus Hospital	60	M	14	103	Walk-In	PTB	CAT-1	Clinical	Treatment Complete
248	Indus Hospital	60	F	14.5	110	Walk-In	PTB	CAT-1	Clinical	Treatment Complete
249	Indus Hospital	72	M	15	108	Screening	PTB	CAT-1	Clinical	Treatment Complete
250	Indus Hospital	132	M	40	140	Walk-In	EPTB	CAT-1	Clinical	Treatment Complete
251	Indus Hospital	24	F	11	90	Walk-In	PTB	CAT-1	Clinical	Treatment Complete
252	Indus Hospital	48	F	3	51	Walk-In	PTB	CAT-1	Clinical	Died
253	Indus Hospital	72	F	10.5	89	Walk-In	PTB	CAT-1	Clinical	Treatment Complete
254	Indus Hospital	156	F	36	154	Walk-In	EPTB	CAT-1	Clinical	Treatment Complete
255	Indus Hospital	48	F	10	90	Walk-In	EPTB	CAT-1	Clinical	Treatment Complete
256	Indus Hospital	24	M	10	78	Walk-In	PTB	CAT-1	Clinical	Treatment Complete
257	Indus Hospital	48	F	13.5	98	Screening	PTB	CAT-1	Clinical	Treatment Complete
258	Indus Hospital	24	M	8.5	77	Walk-In	PTB	CAT-1	Clinical	Treatment Complete
259	Indus Hospital	144	M	22.5	132	Screening	EPTB	CAT-1	Clinical	Treatment Complete
260	Indus Hospital	84	M	19	116	Walk-In	EPTB	CAT-1	Clinical	Treatment Complete
261	Indus Hospital	120	M	24.5	133	Walk-In	PTB	CAT-1	Clinical	Treatment Complete
262	Indus Hospital	144	F	27.5	146	Screening	PTB	CAT-1	Clinical	Treatment Complete
263	Indus Hospital	84	F	16	105	Walk-In	EPTB	CAT-2	Clinical	Treatment Complete
264	Indus Hospital	84	F	14.5	104	Screening	EPTB	CAT-1	Clinical	Treatment Complete
265	Indus Hospital	12	M	9.5	78	Walk-In	PTB	CAT-1	Clinical	Treatment Complete
266	Indus Hospital	24	F	11.5	84	Walk-In	PTB	CAT-1	Clinical	Treatment Complete
267	Indus Hospital	60	M	10.5	90	Walk-In	EPTB	CAT-1	Clinical	Lost to follow up
268	Indus Hospital	72	F	6	68	Walk-In	PTB	CAT-1	Clinical	Treatment Complete
269	Indus Hospital	24	M	11	78	Walk-In	PTB	CAT-1	Clinical	Treatment Complete
270	Indus Hospital	48	M	12	92	Walk-In	EPTB	CAT-1	Clinical	Treatment Complete
271	Indus Hospital	36	M	12	88	Screening	PTB	CAT-1	Clinical	Treatment Complete
272	Indus Hospital	60	M	14.5	101	Walk-In	PTB	CAT-1	Clinical	Treatment Complete
273	Indus Hospital	144	F	31	137	Screening	PTB	CAT-1	Clinical	Treatment Complete
274	Indus Hospital	156	F	31.5	149	Walk-In	EPTB	CAT-1	Clinical	Treatment Complete
275	Indus Hospital	84	F	18	115	Walk-In	EPTB	CAT-1	Clinical	Treatment Complete
276	Indus Hospital	60	F	14	97	Screening	PTB	CAT-1	Clinical	Treatment Complete
277	Indus Hospital	36	M	12	90	Walk-In	PTB	CAT-1	Clinical	Treatment Complete
278	Indus Hospital	48	F	7	65	Walk-In	PTB	CAT-1	Clinical	Lost to follow up
279	Indus Hospital	60	F	13.5	101	Walk-In	PTB	CAT-1	Clinical	Treatment Complete
280	Indus Hospital	120	F	14	120	Walk-In	EPTB	CAT-1	Clinical	Treatment Complete
281	Indus Hospital	36	F	13	91	Walk-In	EPTB	CAT-1	Clinical	Treatment Complete
282	Indus Hospital	144	M	20	137	Screening	PTB	CAT-1	Clinical	Treatment Complete
283	Indus Hospital	156	F	28.5	141	Walk-In	PTB	CAT-1	B+	Treatment Complete
284	Indus Hospital	48	F	11.5	88	Screening	PTB	CAT-1	Clinical	Treatment Complete
285	Indus Hospital	48	F	11.5	88	Screening	PTB	CAT-1	Clinical	Treatment Complete
286	Indus Hospital	60	F	10.5	97	Walk-In	PTB	CAT-1	Clinical	Treatment Complete

287	Indus Hospital	24	F	11.5	96	Screening	PTB	CAT-1	Clinical	Treatment Complete
288	Indus Hospital	84	F	16	117	Walk-In	PTB,EPTB	CAT-1	Clinical	Lost to follow up
289	Indus Hospital	24	M	11	82	Walk-In	PTB	CAT-1	Clinical	Treatment Complete
290	Indus Hospital	84	F	16	112	Walk-In	PTB	CAT-1	Clinical	Treatment Complete
291	Indus Hospital	12	M	9	76	Screening	PTB	CAT-1	Clinical	Treatment Complete
292	Indus Hospital	144	F	24.5	128	Screening	PTB	CAT-1	Clinical	Transfer out
293	Indus Hospital	84	F	18	110	Walk-In	PTB	CAT-1	Clinical	Transfer out
294	Indus Hospital	48	M	15	105	Walk-In	EPTB	CAT-2	Clinical	Treatment Complete
295	Indus Hospital	132	F	31	145	Walk-In	EPTB	CAT-1	Clinical	Treatment Complete
296	Indus Hospital	144	F	14.5	114	Screening	PTB	CAT-1	B+	Cure
297	Indus Hospital	168	M	27	141	Walk-In	PTB	CAT-1	Clinical	Treatment Complete
298	Indus Hospital	120	F	27	138	Walk-In	EPTB	CAT-2	Clinical	Lost to follow up
299	Indus Hospital	144	F	31	138	Walk-In	EPTB	CAT-1	Clinical	Treatment Complete
300	Indus Hospital	144	M	27	152	Screening	PTB	CAT-1	Clinical	Treatment Complete
301	Indus Hospital	144	M	27	135	Walk-In	PTB	CAT-2	Clinical	Treatment Complete
302	Indus Hospital	84	F	21.5	121	Walk-In	EPTB	CAT-1	Clinical	Lost to follow up
303	Indus Hospital	96	F	21	117	Walk-In	PTB	CAT-1	Clinical	Treatment Complete
304	Indus Hospital	24	M	9.5	83	Walk-In	PTB	CAT-2	Clinical	Treatment Complete
305	Indus Hospital	72	M	13.5	101	Walk-In	EPTB	CAT-1	Clinical	Treatment Complete
306	Indus Hospital	120	M	22	125	Walk-In	PTB	CAT-1	Clinical	Treatment Complete
307	Indus Hospital	108	M	19	119	Walk-In	EPTB	CAT-1	Clinical	Treatment Complete
308	Indus Hospital	48	F	12.5	99	Screening	PTB	CAT-1	Clinical	Treatment Complete
309	Indus Hospital	72	M	6.5	67	Screening	PTB	CAT-1	Clinical	Treatment Complete
310	Indus Hospital	144	F	26	141	Walk-In	PTB	CAT-2	Clinical	Treatment Complete
311	Indus Hospital	72	M	8	93	Walk-In	EPTB	CAT-1	Clinical	Treatment Complete
312	Indus Hospital	72	F	17.5	112	Walk-In	PTB	CAT-1	Clinical	Treatment Complete
313	Indus Hospital	156	M	35.5	162	Walk-In	PTB	CAT-1	B+	Failure
314	Indus Hospital	96	M	22	121	Walk-In	EPTB	CAT-2	Clinical	Treatment Complete
315	Indus Hospital	84	M	8.5	66	Walk-In	PTB	CAT-1	Clinical	Treatment Complete
316	Indus Hospital	48	M	13.5	95	Screening	PTB	CAT-1	Clinical	Treatment Complete
317	Indus Hospital	24	F	9	72	Walk-In	PTB	CAT-1	Clinical	Treatment Complete
318	Indus Hospital	72	M	21.5	119	Walk-In	EPTB	CAT-1	Clinical	Treatment Complete
319	Indus Hospital	60	F	9.5	84	Walk-In	PTB	CAT-2	Clinical	Treatment Complete
320	Indus Hospital	168	M	29.5	150	Walk-In	PTB	CAT-1	Clinical	Treatment Complete
321	Indus Hospital	96	M	15.5	106	Walk-In	PTB	CAT-2	Clinical	Treatment Complete
322	Indus Hospital	24	F	11	83	Walk-In	EPTB	CAT-2	Clinical	Treatment Complete
323	Indus Hospital	144	F	27.5	145	Walk-In	EPTB	CAT-1	Clinical	Treatment Complete
324	Indus Hospital	72	F	14	103	Walk-In	EPTB	CAT-1	Clinical	Treatment Complete
325	Indus Hospital	168	F	28	142	Walk-In	PTB	CAT-1	Clinical	Treatment Complete
326	Indus Hospital	24	M	7	78	Walk-In	PTB	CAT-1	Clinical	Treatment Complete
327	Indus Hospital	48	F	14.5	107	Walk-In	EPTB	CAT-1	Clinical	Treatment Complete

328	Indus Hospital	132	F	33	143	Screening	PTB	CAT-1	Clinical	Treatment Complete
329	Indus Hospital	96	F	26	130	Screening	PTB	CAT-1	Clinical	Treatment Complete
330	Indus Hospital	132	M	23.5	133	Walk-In	PTB	CAT-2	Clinical	Treatment Complete
331	Indus Hospital	48	F	13	102	Walk-In	PTB	CAT-1	Clinical	Treatment Complete
332	Indus Hospital	60	M	19	112	Walk-In	PTB	CAT-1	Clinical	Treatment Complete
333	Indus Hospital	84	F	22	119	Walk-In	PTB	CAT-1	Clinical	Treatment Complete
334	Indus Hospital	156	M	30	155	Walk-In	PTB	CAT-1	Clinical	Treatment Complete
335	Indus Hospital	144	F	25.5	129	Walk-In	PTB	CAT-1	Clinical	Lost to follow up
336	Indus Hospital	168	F	37	147	Walk-In	PTB	CAT-1	Clinical	Treatment Complete
337	Indus Hospital	96	F	19.5	120	Walk-In	PTB	CAT-1	B+	Treatment Complete
338	Indus Hospital	60	F	14	100	Walk-In	PTB	CAT-1	Clinical	Treatment Complete
339	Indus Hospital	132	F	18	131	Walk-In	PTB	CAT-1	Clinical	Treatment Complete
340	Indus Hospital	192	M	16	115	Walk-In	EPTB	CAT-1	Clinical	Treatment Complete
341	Indus Hospital	156	F	29	150	Walk-In	PTB	CAT-1	B+	Cure
342	Indus Hospital	24	F	34	156	Walk-In	EPTB	CAT-1	Clinical	Treatment Complete
343	Indus Hospital	132	F	21	134	Screening	PTB	CAT-1	Clinical	Treatment Complete
344	Indus Hospital	24	F	9	75	Walk-In	PTB	CAT-1	Clinical	Treatment Complete
345	Indus Hospital	168	F	37	158	Walk-In	PTB	CAT-1	B+	Cure
346	Indus Hospital	120	F	19	116	Walk-In	EPTB	CAT-1	Clinical	Treatment Complete
347	Indus Hospital	84	F	18	117	Walk-In	EPTB	CAT-1	Clinical	Cure
348	Indus Hospital	168	F	26.5	153	Walk-In	EPTB	CAT-1	Clinical	Treatment Complete
349	Indus Hospital	96	M	6.5	64	Walk-In	PTB	CAT-1	Clinical	Treatment Complete
350	Indus Hospital	120	F	24.5	138	Walk-In	PTB	CAT-1	Clinical	Treatment Complete
351	Indus Hospital	132	F	23	120	Walk-In	PTB	CAT-1	Clinical	Treatment Complete
352	Indus Hospital	156	F	33.5	148	Screening	PTB	CAT-1	Clinical	Treatment Complete
353	Indus Hospital	120	F	16.5	126	Walk-In	PTB,EPTB	CAT-1	Clinical	Treatment Complete
354	Indus Hospital	144	F	37.5	143	Walk-In	EPTB	CAT-1	Clinical	Treatment Complete
355	Indus Hospital	36	F	9	82	Walk-In	PTB	CAT-1	Clinical	Treatment Complete
356	Indus Hospital	144	F	36.5	153	Walk-In	PTB	CAT-1	B+	Transfer out
357	Indus Hospital	144	F	25	144	Walk-In	PTB	CAT-1	Clinical	Treatment Complete
358	Indus Hospital	144	F	62	153	Walk-In	PTB	CAT-1	Clinical	Treatment Complete
359	Indus Hospital	120	M	17	114	Walk-In	PTB	CAT-1	Clinical	Treatment Complete
360	Indus Hospital	144	F	38	146	Walk-In	PTB,EPTB	CAT-1	Clinical	Treatment Complete
361	Indus Hospital	120	F	7	72	Walk-In	PTB	CAT-1	Clinical	Treatment Complete
362	Indus Hospital	12	M	15	113	Walk-In	PTB	CAT-1	Clinical	Lost to follow up
363	Indus Hospital	84	F	12.5	108	Walk-In	PTB	CAT-1	Clinical	Treatment Complete
364	Indus Hospital	108	M	16	122	Walk-In	PTB	CAT-1	B+	Treatment Complete
365	Indus Hospital	108	F	15.5	117	Walk-In	PTB	CAT-1	Clinical	Died
366	Indus Hospital	12	M	10	79	Walk-In	EPTB	CAT-1	Clinical	Treatment Complete
367	Indus Hospital	12	M	8	70	Screening	PTB	CAT-1	Clinical	Treatment Complete
368	Indus Hospital	48	F	11	87	Walk-In	EPTB	CAT-2	B+	Treatment Complete

369	Indus Hospital	48	F	12.5	94	Walk-In	PTB	CAT-1	Clinical	Treatment Complete
370	Indus Hospital	144	F	16	130	Walk-In	EPTB	CAT-1	Clinical	Treatment Complete
371	Indus Hospital	168	F	39	145	Walk-In	PTB	CAT-1	Clinical	Treatment Complete
372	Indus Hospital	168	M	38	156	Walk-In	PTB	CAT-1	Clinical	Treatment Complete
373	Indus Hospital	72	F	17	108	Walk-In	PTB	CAT-1	Clinical	Lost to follow up
374	Indus Hospital	144	M	23	135	Walk-In	PTB	CAT-2	B+	Treatment Complete
375	Indus Hospital	156	F	23	146	Walk-In	PTB	CAT-1	Clinical	Lost to follow up
376	Indus Hospital	156	F	29.5	136	Walk-In	PTB	CAT-1	Clinical	Treatment Complete
377	Indus Hospital	84	M	17.5	118	Walk-In	EPTB	CAT-1	Clinical	Treatment Complete
378	Indus Hospital	156	F	37.5	166	Walk-In	PTB	CAT-1	Clinical	Treatment Complete
379	Indus Hospital	48	F	9	85	Walk-In	PTB	CAT-1	Clinical	Treatment Complete
380	Indus Hospital	108	F	18.5	125	Walk-In	PTB	CAT-1	Clinical	Treatment Complete
381	Indus Hospital	96	F	24.5	127	Walk-In	PTB	CAT-1	Clinical	Transfer out
382	Indus Hospital	60	F	14.5	105	Walk-In	EPTB	CAT-2	Clinical	Treatment Complete
383	Indus Hospital	168	F	35	141	Walk-In	PTB	CAT-1	Clinical	Treatment Complete
384	Indus Hospital	36	M	11	90	Walk-In	EPTB	CAT-1	B+	Treatment Complete
385	Indus Hospital	144	F	23	140	Walk-In	EPTB	CAT-1	Clinical	Treatment Complete
386	Indus Hospital	108	F	20	120	Walk-In	PTB	CAT-1	Clinical	Treatment Complete
387	Indus Hospital	36	F	11	19	Walk-In	PTB	CAT-2	Clinical	Treatment Complete
388	Indus Hospital	120	F	30	136	Walk-In	PTB	CAT-1	B+	Cure
389	Indus Hospital	24	M	8	76	Walk-In	PTB	CAT-1	Clinical	Treatment Complete
390	Indus Hospital	120	M	17.5	119	Walk-In	PTB	CAT-1	B+	Lost to follow up
391	Indus Hospital	96	F	12	117	Walk-In	PTB	CAT-1	Clinical	Treatment Complete
392	Indus Hospital	168	F	34	146	Walk-In	PTB,EPTB	CAT-1	Clinical	Treatment Complete
393	Indus Hospital	132	M	25	114	Walk-In	PTB	CAT-1	Clinical	Treatment Complete
394	Indus Hospital	12	F	4	62	Walk-In	PTB	CAT-1	Clinical	Treatment Complete
395	Indus Hospital	72	F	14	104	Walk-In	EPTB	CAT-1	Clinical	Treatment Complete
396	Indus Hospital	168	F	36	152	Walk-In	PTB	CAT-1	B+	Cure
397	Indus Hospital	120	M	17	125	Walk-In	EPTB	CAT-1	Clinical	Treatment Complete
398	Indus Hospital	156	F	13	133	Walk-In	PTB	CAT-2	Clinical	Treatment Complete
399	Indus Hospital	168	F	38	153	Walk-In	PTB	CAT-1	Clinical	Lost to follow up
400	Indus Hospital	144	F	22	135	Walk-In	PTB	CAT-2	Clinical	Transfer out
401	Indus Hospital	84	M	16	112	Walk-In	PTB,EPTB	CAT-1	Clinical	Treatment Complete
402	Indus Hospital	144	F	12	33	Walk-In	EPTB	CAT-1	Clinical	Treatment Complete
403	Indus Hospital	144	F	15.5	116	Walk-In	PTB	CAT-1	Clinical	Treatment Complete
404	Indus Hospital	156	F	32	144	Walk-In	PTB	CAT-1	Clinical	Treatment Complete
405	Indus Hospital	84	F	11.5	106	Walk-In	PTB	CAT-1	B+	Cure
406	Indus Hospital	144	M	37	151	Walk-In	EPTB	CAT-1	Clinical	Treatment Complete
407	Indus Hospital	168	F	37	159	Walk-In	PTB	CAT-1	B+	Treatment Complete
408	Indus Hospital	156	F	33	145	Walk-In	PTB	CAT-1	Clinical	Treatment Complete
409	Indus Hospital	132	F	28	146	Walk-In	PTB,EPTB	CAT-1	Clinical	Treatment Complete

410	Indus Hospital	96	F	18	110	Walk-In	EPTB	CAT-1	Clinical	Treatment Complete
411	Indus Hospital	60	M	11	94	Walk-In	PTB	CAT-2	Clinical	Treatment Complete
412	Indus Hospital	144	F	37	153	Walk-In	EPTB	CAT-1	Clinical	Treatment Complete
413	Indus Hospital	144	M	38	157	Walk-In	PTB	CAT-1	Clinical	Treatment Complete
414	Indus Hospital	72	F	12.5	106	Walk-In	EPTB	CAT-1	Clinical	Treatment Complete
415	Indus Hospital	84	M	15	107	Walk-In	EPTB	CAT-1	Clinical	Treatment Complete
416	Indus Hospital	156	F	37	143	Walk-In	EPTB	CAT-1	Clinical	Treatment Complete
417	Indus Hospital	168	M	30	155	Screening	EPTB	CAT-1	Clinical	Treatment Complete
418	Indus Hospital	132	F	22	137	Walk-In	EPTB	CAT-1	Clinical	Treatment Complete
419	Indus Hospital	168	M	17	123	Walk-In	EPTB	CAT-1	Clinical	Treatment Complete
420	Indus Hospital	120	F	17	128	Walk-In	EPTB	CAT-1	Clinical	Treatment Complete
421	Indus Hospital	84	M	15	106	Walk-In	PTB	CAT-1	Clinical	Treatment Complete
422	Indus Hospital	132	F	21	137	Walk-In	EPTB	CAT-2	Clinical	Treatment Complete
423	Indus Hospital	132	F	16.5	123	Walk-In	PTB	CAT-1	Clinical	Treatment Complete
424	Indus Hospital	132	F	21.5	132	Walk-In	EPTB	CAT-1	Clinical	Lost to follow up
425	Indus Hospital	60	M	13.5	97	Walk-In	EPTB	CAT-1	Clinical	Treatment Complete
426	Indus Hospital	156	F	28	150	Walk-In	PTB	CAT-1	Clinical	Treatment Complete
427	Indus Hospital	60	M	14	106	Walk-In	EPTB	CAT-1	Clinical	Treatment Complete
428	Indus Hospital	60	F	12	90	Walk-In	EPTB	CAT-1	Clinical	Treatment Complete
429	Indus Hospital	60	M	13	98	Walk-In	EPTB	CAT-1	Clinical	Treatment Complete
430	Indus Hospital	72	F	6.5	62	Walk-In	EPTB	CAT-2	Clinical	Failure
431	Indus Hospital	168	F	32	140	Walk-In	EPTB	CAT-1	Clinical	Treatment Complete
432	Indus Hospital	60	M	5.5	66	Walk-In	PTB	CAT-1	Clinical	Treatment Complete
433	Indus Hospital	72	M	15	115	Walk-In	PTB	CAT-1	B+	Failure
434	Indus Hospital	132	M	8.5	65	Walk-In	PTB	CAT-1	Clinical	Treatment Complete
435	Indus Hospital	60	M	12	92	Walk-In	PTB	CAT-2	Clinical	Treatment Complete
436	Indus Hospital	144	M	17.5	123	Walk-In	PTB	CAT-1	Clinical	Treatment Complete
437	Indus Hospital	168	F	34	140	Walk-In	PTB	CAT-1	Clinical	Treatment Complete
438	Indus Hospital	132	M	21	123	Walk-In	PTB	CAT-1	Clinical	Treatment Complete
439	Indus Hospital	96	F	14	106	Walk-In	PTB	CAT-1	Clinical	Lost to follow up
440	Indus Hospital	72	M	19	118	Walk-In	EPTB	CAT-1	Clinical	Treatment Complete
441	Indus Hospital	144	F	26	135	Walk-In	PTB	CAT-1	Clinical	Treatment Complete
442	Indus Hospital	132	F	25	138	Walk-In	PTB	CAT-1	Clinical	Treatment Complete
443	Indus Hospital	120	F	29	138	Walk-In	EPTB	CAT-1	Clinical	Treatment Complete
444	Indus Hospital	24	F	9	86	Screening	PTB	CAT-1	Clinical	Treatment Complete
445	Indus Hospital	156	M	24	137	Walk-In	PTB	CAT-1	Clinical	Treatment Complete
446	Indus Hospital	132	M	28	142	Walk-In	PTB	CAT-1	Clinical	Treatment Complete
447	Indus Hospital	168	M	38	162	Walk-In	PTB,EPTB	CAT-1	Clinical	Treatment Complete
448	Indus Hospital	120	F	26	140	Walk-In	EPTB	CAT-1	Clinical	Treatment Complete
449	Indus Hospital	36	M	10	86	Walk-In	PTB	CAT-1	Clinical	Treatment Complete
450	Indus Hospital	156	F	30	154	Walk-In	EPTB	CAT-2	Clinical	Treatment Complete

451	Indus Hospital	168	F	41	151	Walk-In	PTB	CAT-2	B+	Cure
452	Indus Hospital	36	F	10	83	Walk-In	PTB	CAT-1	Clinical	Treatment Complete
453	Indus Hospital	108	F	18	118	Walk-In	EPTB	CAT-1	B+	Treatment Complete
454	Indus Hospital	156	F	18	59	Walk-In	EPTB	CAT-1	B+	Treatment Complete
455	Indus Hospital	120	F	26	138	Walk-In	EPTB	CAT-1	B+	Treatment Complete
456	Indus Hospital	168	F	37	154	Walk-In	EPTB	CAT-1	Clinical	Treatment Complete
457	Indus Hospital	120	F	19	121	Walk-In	PTB	CAT-1	Clinical	Treatment Complete
458	Indus Hospital	60	F	12	98	Walk-In	EPTB	CAT-1	B+	Treatment Complete
459	Indus Hospital	60	F	15	98	Walk-In	PTB	CAT-1	Clinical	Treatment Complete
460	Indus Hospital	84	M	18	120	Walk-In	EPTB	CAT-1	Clinical	Treatment Complete
461	Indus Hospital	120	M	25	137	Walk-In	EPTB	CAT-1	Clinical	Lost to follow up
462	Indus Hospital	120	F	7	72	Walk-In	PTB	CAT-1	Clinical	Lost to follow up
463	Indus Hospital	24	M	11	87	Walk-In	PTB	CAT-1	B+	Failure
464	Indus Hospital	84	M	20	125	Walk-In	EPTB	CAT-1	Clinical	Treatment Complete
465	Indus Hospital	12	M	10	71	Walk-In	PTB	CAT-1	Clinical	Lost to follow up
466	Indus Hospital	72	M	5	56	Walk-In	PTB	CAT-1	Clinical	Treatment Complete
467	Indus Hospital	108	F	24	124	Walk-In	PTB	CAT-1	B+	Failure
468	Indus Hospital	156	F	30	147	Walk-In	PTB	CAT-1	Clinical	Failure
469	Indus Hospital	156	F	40	150	Walk-In	PTB,EPTB	CAT-1	B+	Failure
470	Indus Hospital	120	M	29	146	Walk-In	PTB	CAT-1	B+	Treatment Complete
471	Indus Hospital	144	F	20	130	Walk-In	PTB	CAT-1	Clinical	Lost to follow up
472	Indus Hospital	156	F	38	151	Screening	PTB	CAT-1	B+	Treatment Complete
473	Indus Hospital	132	F	18	120	Walk-In	EPTB	CAT-1	Clinical	Treatment Complete
474	Indus Hospital	60	F	15	111	Walk-In	EPTB	CAT-1	Clinical	Treatment Complete
475	Indus Hospital	12	M	8	70	Walk-In	EPTB	CAT-1	Clinical	Treatment Complete
476	Indus Hospital	132	F	25	132	Walk-In	EPTB	CAT-1	Clinical	Treatment Complete
477	Indus Hospital	120	F	21.5	123	Walk-In	PTB	CAT-1	B+	Treatment Complete
478	Indus Hospital	156	F	27	133	Walk-In	EPTB	CAT-1	Clinical	Treatment Complete
479	Indus Hospital	156	M	26.5	138	Walk-In	EPTB	CAT-1	Clinical	Treatment Complete
480	Indus Hospital	156	F	37	143	Walk-In	EPTB	CAT-1	Clinical	Treatment Complete
481	Indus Hospital	84	F	18	113	Walk-In	PTB	CAT-2	Clinical	Treatment Complete
482	Indus Hospital	24	F	8	76	Walk-In	EPTB	CAT-1	Clinical	Treatment Complete
483	Indus Hospital	48	M	11	96	Screening	EPTB	CAT-1	Clinical	Treatment Complete
484	Indus Hospital	48	F	13.5	94	Walk-In	PTB	CAT-1	Clinical	Treatment Complete
485	Indus Hospital	12	F	9	75	Walk-In	PTB	CAT-1	Clinical	Treatment Complete
486	Indus Hospital	24	M	10	80	Walk-In	PTB	CAT-1	Clinical	Treatment Complete
487	Indus Hospital	120	F	48	140	Walk-In	EPTB	CAT-1	Clinical	Treatment Complete
488	Indus Hospital	24	F	7	70	Walk-In	PTB	CAT-1	Clinical	Treatment Complete
489	Indus Hospital	132	M	29.5	139	Walk-In	EPTB	CAT-1	Clinical	Treatment Complete
490	Indus Hospital	36	M	9.5	81	Walk-In	PTB	CAT-1	Clinical	Treatment Complete
491	Indus Hospital	144	M	21.5	133	Walk-In	EPTB	CAT-1	Clinical	Treatment Complete

492	Indus Hospital	24	M	8.5	70	Walk-In	PTB	CAT-1	Clinical	Treatment Complete
493	Indus Hospital	96	M	22	124	Walk-In	EPTB	CAT-1	Clinical	Treatment Complete
494	Indus Hospital	144	F	28	144	Walk-In	EPTB	CAT-1	Clinical	Treatment Complete
495	Indus Hospital	84	F	12.5	94	Walk-In	EPTB	CAT-1	Clinical	Still on treatment
496	Indus Hospital	168	M	30	146	Walk-In	PTB	CAT-1	Clinical	Lost to follow up
497	Indus Hospital	60	F	18	111	Walk-In	EPTB	CAT-1	Clinical	Lost to follow up
498	Indus Hospital	120	F	19	122	Walk-In	PTB	CAT-2	Clinical	Treatment Complete
499	Indus Hospital	84	F	19	114	Walk-In	PTB	CAT-1	Clinical	Treatment Complete
500	Indus Hospital	168	M	40.5	157	Screening	PTB	CAT-1	Clinical	Treatment Complete
501	Indus Hospital	144	F	30	140	Walk-In	PTB	CAT-1	Clinical	Treatment Complete
502	Indus Hospital	144	F	25	126	Walk-In	PTB	CAT-2	B+	Treatment Complete
503	Indus Hospital	132	F	26	139	Walk-In	PTB	CAT-1	Clinical	Treatment Complete
504	Indus Hospital	144	F	17	110	Walk-In	PTB	CAT-1	Clinical	Lost to follow up
505	Indus Hospital	144	F	35	140	Screening	PTB	CAT-1	Clinical	Treatment Complete
506	Indus Hospital	168	F	37	160	Walk-In	EPTB	CAT-1	Clinical	Treatment Complete
507	Indus Hospital	168	F	39	154	Walk-In	PTB,EPTB	CAT-1	Clinical	Treatment Complete
508	Indus Hospital	132	F	28	149	Walk-In	EPTB	CAT-1	Clinical	Treatment Complete
509	Indus Hospital	156	F	35	156	Walk-In	PTB	CAT-1	B+	Treatment Complete
510	Indus Hospital	12	M	7	71	Walk-In	PTB	CAT-1	Clinical	Treatment Complete
511	Indus Hospital	60	M	13	64	Walk-In	PTB	CAT-1	Clinical	Treatment Complete
512	Indus Hospital	156	F	29	140	Walk-In	PTB	CAT-1	B+	Treatment Complete
513	Indus Hospital	36	M	10.5	90	Walk-In	EPTB	CAT-1	Clinical	Treatment Complete
514	Indus Hospital	144	F	22	139	Screening	PTB	CAT-1	B+	Failure
515	Indus Hospital	108	F	16.5	114	Screening	PTB	CAT-1	Clinical	Treatment Complete
516	Indus Hospital	120	M	28	141	Walk-In	PTB	CAT-1	Clinical	Treatment Complete
517	Indus Hospital	48	M	10	95	Walk-In	PTB	CAT-1	Clinical	Treatment Complete
518	Indus Hospital	84	F	22	125	Walk-In	PTB	CAT-1	Clinical	Treatment Complete
519	Indus Hospital	120	M	24.5	125	Walk-In	PTB	CAT-1	Clinical	Treatment Complete
520	Indus Hospital	96	F	60	167	Walk-In	EPTB	CAT-2	Clinical	Treatment Complete
521	Indus Hospital	96	M	9	104	Walk-In	PTB	CAT-1	Clinical	Treatment Complete
522	Indus Hospital	108	M	19.3	108	Walk-In	EPTB	CAT-1	Clinical	Treatment Complete
523	Indus Hospital	60	F	12	92	Walk-In	EPTB	CAT-1	Clinical	Failure
524	Indus Hospital	12	F	9	80	Walk-In	EPTB	CAT-1	Clinical	Lost to follow up
525	Indus Hospital	24	F	7.5	78	Screening	PTB	CAT-1	Clinical	Treatment Complete
526	Indus Hospital	120	F	20	122	Screening	PTB	CAT-1	Clinical	Treatment Complete
527	Indus Hospital	132	F	22	128	Walk-In	PTB	CAT-2	B+	Failure
528	Indus Hospital	132	F	20	125	Walk-In	EPTB	CAT-1	Clinical	Treatment Complete
529	Indus Hospital	168	M	38	153	Walk-In	EPTB	CAT-1	Clinical	Treatment Complete
530	Indus Hospital	12	F	8	70	Walk-In	EPTB	CAT-1	Clinical	Treatment Complete
531	Indus Hospital	168	F	31	136	Walk-In	EPTB	CAT-1	Clinical	Treatment Complete
532	Indus Hospital	108	F	25	128	Walk-In	PTB	CAT-1	Clinical	Treatment Complete

533	Indus Hospital	84	M	19.5	124	Walk-In	PTB	CAT-1	Clinical	Treatment Complete
534	Indus Hospital	36	F	11	85	Walk-In	PTB	CAT-1	Clinical	Treatment Complete
535	Indus Hospital	120	F	19	124	Walk-In	PTB	CAT-1	Clinical	Treatment Complete
536	Indus Hospital	144	F	28	143	Walk-In	PTB	CAT-1	B+	Treatment Complete
537	Indus Hospital	108	F	16	118	Walk-In	EPTB	CAT-1	Clinical	Treatment Complete
538	Indus Hospital	36	F	11.5	83	Walk-In	PTB	CAT-1	Clinical	Treatment Complete
539	Indus Hospital	156	F	29	142	Walk-In	PTB	CAT-1	B+	Treatment Complete
540	Indus Hospital	12	M	9.5	75	Walk-In	PTB	CAT-1	Clinical	Treatment Complete
541	Indus Hospital	168	M	29	146	Walk-In	PTB	CAT-1	B+	Treatment Complete
542	Indus Hospital	144	M	26	137	Walk-In	PTB	CAT-1	B+	Died
543	Indus Hospital	96	M	19.3	108	Walk-In	EPTB	CAT-1	Clinical	Still on treatment
544	Indus Hospital	120	F	18.5	118	Walk-In	PTB	CAT-1	Clinical	Treatment Complete
545	Indus Hospital	60	M	19	116	Walk-In	PTB	CAT-1	Clinical	Treatment Complete
546	Indus Hospital	84	M	19	120	Walk-In	EPTB	CAT-1	Clinical	Treatment Complete
547	Indus Hospital	120	M	19.5	124	Walk-In	EPTB	CAT-1	Clinical	Treatment Complete
548	Indus Hospital	72	M	13	102	Walk-In	PTB	CAT-1	Clinical	Treatment Complete
549	Indus Hospital	36	F	10.5	85	Walk-In	PTB	CAT-2	Clinical	Treatment Complete
550	Indus Hospital	84	F	10	101	Walk-In	PTB	CAT-1	Clinical	Died
551	Indus Hospital	144	F	22	127	Walk-In	EPTB	CAT-2	Clinical	Treatment Complete
552	Indus Hospital	156	F	30.5	144	Walk-In	PTB	CAT-1	Clinical	Treatment Complete
553	Indus Hospital	48	F	12	92	Walk-In	PTB	CAT-1	Clinical	Treatment Complete
554	Indus Hospital	84	F	21.5	124	Walk-In	PTB	CAT-2	Clinical	Treatment Complete
555	Indus Hospital	96	M	18	118	Walk-In	EPTB	CAT-1	Clinical	Still on treatment
556	Indus Hospital	24	M	10	80	Walk-In	PTB	CAT-1	Clinical	Treatment Complete
557	Indus Hospital	144	F	16	120	Walk-In	PTB	CAT-1	B+	Treatment Complete
558	Indus Hospital	12	F	6	70	Walk-In	PTB	CAT-1	Clinical	Treatment Complete
559	Indus Hospital	156	F	32	150	Walk-In	PTB	CAT-1	B+	Treatment Complete
560	Indus Hospital	156	F	34	147	Walk-In	PTB	CAT-1	B+	Failure
561	Indus Hospital	60	F	13	96	Walk-In	PTB	CAT-1	Clinical	Treatment Complete
562	Indus Hospital	72	M	17	110	Walk-In	PTB	CAT-1	Clinical	Treatment Complete
563	Indus Hospital	96	M	19	119	Walk-In	PTB	CAT-1	Clinical	Treatment Complete
564	Indus Hospital	156	F	23.5	144	Walk-In	PTB	CAT-1	B+	Treatment Complete
565	Indus Hospital	156	F	34	151	Walk-In	PTB,EPTB	CAT-1	Clinical	Treatment Complete
566	Indus Hospital	48	M	13	97	Walk-In	PTB	CAT-1	Clinical	Treatment Complete
567	Indus Hospital	132	F	35	147	Walk-In	EPTB	CAT-1	Clinical	Treatment Complete
568	Indus Hospital	84	M	21	123	Walk-In	EPTB	CAT-1	Clinical	Treatment Complete
569	Indus Hospital	120	F	20.3	135	Walk-In	PTB,EPTB	CAT-1	Clinical	Lost to follow up
570	Indus Hospital	156	F	32	147	Walk-In	PTB	CAT-1	Clinical	Treatment Complete
571	Indus Hospital	24	F	8	82	Walk-In	PTB	CAT-1	Clinical	Treatment Complete
572	Indus Hospital	156	F	38	146	Walk-In	PTB	CAT-2	B+	Treatment Complete
573	Indus Hospital	12	F	9	72	Walk-In	PTB	CAT-1	Clinical	Treatment Complete

574	Indus Hospital	168	F	35	148	Walk-In	EPTB	CAT-1	Clinical	Treatment Complete
575	Indus Hospital	132	F	5.5	66	Walk-In	PTB	CAT-1	Clinical	Treatment Complete
576	Indus Hospital	72	F	16	104	Walk-In	PTB	CAT-1	Clinical	Treatment Complete
577	Indus Hospital	120	M	21	121	Walk-In	PTB	CAT-1	Clinical	Treatment Complete
578	Indus Hospital	36	F	8.5	82	Walk-In	PTB	CAT-1	Clinical	Treatment Complete
579	Indus Hospital	132	M	21	126	Walk-In	PTB	CAT-1	Clinical	Treatment Complete
580	Indus Hospital	168	F	40	155	Walk-In	PTB	CAT-1	Clinical	Treatment Complete
581	Indus Hospital	144	F	27	144	Walk-In	PTB	CAT-1	Clinical	Treatment Complete
582	Indus Hospital	156	F	27	146	Walk-In	PTB	CAT-1	Clinical	Treatment Complete
583	Indus Hospital	96	F	20.5	128	Walk-In	PTB	CAT-1	Clinical	Treatment Complete
584	Indus Hospital	72	F	14	107	Walk-In	EPTB	CAT-1	Clinical	Transfer out
585	Indus Hospital	60	M	10	93	Walk-In	PTB	CAT-1	Clinical	Treatment Complete
586	Indus Hospital	48	M	9.5	87	Walk-In	PTB	CAT-1	Clinical	Treatment Complete
587	Indus Hospital	84	F	N/A	N/A	Walk-In	EPTB	CAT-1	Clinical	Lost to follow up
588	Indus Hospital	12	M	8	71	Walk-In	PTB	CAT-1	Clinical	Treatment Complete
589	Indus Hospital	120	F	20	128	Walk-In	PTB	CAT-1	Clinical	Treatment Complete
590	Indus Hospital	108	F	19	123	Walk-In	PTB	CAT-1	B+	Treatment Complete
591	Indus Hospital	72	M	16.5	113	Walk-In	PTB	CAT-1	Clinical	Treatment Complete
592	Indus Hospital	84	M	18.5	120	Walk-In	PTB	CAT-1	Clinical	Treatment Complete
593	Indus Hospital	84	F	19	116	Walk-In	EPTB	CAT-1	Clinical	Lost to follow up
594	Indus Hospital	120	F	15.5	112	Walk-In	EPTB	CAT-1	Clinical	Treatment Complete
595	Indus Hospital	12	F	6	68	Walk-In	PTB	CAT-1	Clinical	Treatment Complete
596	Indus Hospital	108	F	25	127	Walk-In	PTB	CAT-2	Clinical	Lost to follow up
597	Indus Hospital	72	M	5.5	67	Walk-In	PTB	CAT-1	Clinical	Lost to follow up
598	Indus Hospital	120	F	16	122	Walk-In	EPTB	CAT-1	Clinical	Still on treatment
599	Indus Hospital	48	M	13	100	Walk-In	EPTB	CAT-1	Clinical	Treatment Complete
600	Indus Hospital	72	F	15.5	104	Walk-In	EPTB	CAT-1	Clinical	Treatment Complete
601	Indus Hospital	108	M	20	124	Walk-In	EPTB	CAT-1	Clinical	Still on treatment
602	Indus Hospital	12	M	7	70	Walk-In	EPTB	CAT-1	Clinical	Treatment Complete
603	Indus Hospital	168	F	48	156	Walk-In	PTB	CAT-1	Clinical	Treatment Complete
604	Indus Hospital	72	F	11	97	Walk-In	PTB	CAT-1	Clinical	Treatment Complete
605	Indus Hospital	156	M	20.5	133	Walk-In	EPTB	CAT-1	Clinical	Treatment Complete
606	Indus Hospital	108	F	19	125	Walk-In	EPTB	CAT-1	Clinical	Still on treatment
607	Indus Hospital	96	M	20	122	Walk-In	PTB	CAT-1	Clinical	Treatment Complete
608	Indus Hospital	156	M	31.5	146	Walk-In	EPTB	CAT-1	Clinical	Treatment Complete
609	Indus Hospital	48	M	14.5	100	Walk-In	PTB	CAT-1	Clinical	Treatment Complete
610	Indus Hospital	120	F	21	128	Walk-In	PTB	CAT-1	Clinical	Treatment Complete
611	Indus Hospital	144	F	29	142	Walk-In	EPTB	CAT-1	Clinical	Treatment Complete
612	Indus Hospital	12	M	5.5	65	Walk-In	PTB	CAT-1	Clinical	Treatment Complete
613	Indus Hospital	84	M	17.5	117	Walk-In	EPTB	CAT-1	Clinical	Still on treatment
614	Indus Hospital	168	F	35.5	154	Walk-In	EPTB	CAT-1	Clinical	Lost to follow up

615	Indus Hospital	84	F	18	124	Walk-In	EPTB	CAT-1	Clinical	Treatment Complete
616	Indus Hospital	12	M	6.6	74	Walk-In	EPTB	CAT-1	Clinical	Still on treatment
617	Indus Hospital	12	F	7	68	Walk-In	PTB	CAT-1	Clinical	Treatment Complete
618	Indus Hospital	48	F	13	87	Walk-In	PTB	CAT-1	Clinical	Treatment Complete
619	Indus Hospital	96	M	18.5	124	Walk-In	PTB	CAT-1	Clinical	Treatment Complete
620	Indus Hospital	144	F	21.5	141	Walk-In	PTB	CAT-1	Clinical	Died
621	Indus Hospital	36	F	9.5	88	Walk-In	PTB	CAT-1	Clinical	Treatment Complete
622	Indus Hospital	36	F	9	80	Walk-In	PTB	CAT-1	Clinical	Treatment Complete
623	Indus Hospital	144	M	32	151	Walk-In	EPTB	CAT-2	Clinical	Still on treatment
624	Indus Hospital	144	F	21	130	Walk-In	EPTB	CAT-1	Clinical	Treatment Complete
625	Indus Hospital	144	M	24	139	Walk-In	PTB	CAT-1	Clinical	Treatment Complete
626	Indus Hospital	12	M	8	72	Walk-In	PTB	CAT-1	Clinical	Treatment Complete
627	Indus Hospital	120	F	23.5	143	Walk-In	PTB	CAT-1	Clinical	Treatment Complete
628	Indus Hospital	108	F	17	117	Walk-In	PTB	CAT-1	Clinical	Treatment Complete
629	Indus Hospital	60	F	12	106	Walk-In	EPTB	CAT-1	Clinical	Still on treatment
630	Indus Hospital	60	M	11	94	Walk-In	EPTB	CAT-1	Clinical	Lost to follow up
631	Indus Hospital	60	M	11	97	Walk-In	PTB	CAT-1	Clinical	Treatment Complete
632	Indus Hospital	60	F	15	107	Walk-In	EPTB	CAT-1	Clinical	Still on treatment
633	Indus Hospital	120	F	25	136	Walk-In	EPTB	CAT-1	Clinical	Treatment Complete
634	Indus Hospital	156	M	21.5	129	Walk-In	PTB	CAT-1	Clinical	Treatment Complete
635	Indus Hospital	12	F	4.5	72	Walk-In	PTB	CAT-1	Clinical	Treatment Complete
636	Indus Hospital	72	M	17	113	Walk-In	EPTB	CAT-1	Clinical	Treatment Complete
637	Indus Hospital	84	M	17.5	118	Walk-In	PTB	CAT-1	Clinical	Treatment Complete
638	Indus Hospital	12	F	6	71	Walk-In	PTB	CAT-1	Clinical	Treatment Complete
639	Indus Hospital	144	F	29	140	Walk-In	PTB	CAT-1	Clinical	Treatment Complete
640	Indus Hospital	144	M	21	130	Walk-In	PTB	CAT-1	Clinical	Died
641	Indus Hospital	108	M	12	117	Walk-In	EPTB	CAT-1	Clinical	Still on treatment
642	Indus Hospital	108	F	28	138	Walk-In	PTB	CAT-1	Clinical	Treatment Complete
643	Indus Hospital	48	F	4.5	43	Walk-In	PTB	CAT-1	Clinical	Died
644	Indus Hospital	36	F	11	93	Walk-In	PTB	CAT-1	Clinical	Treatment Complete
645	Indus Hospital	48	M	15	96	Walk-In	PTB	CAT-1	Clinical	Treatment Complete
646	Indus Hospital	96	F	16	116	Walk-In	PTB	CAT-1	Clinical	Treatment Complete
647	Indus Hospital	108	M	9	75	Walk-In	EPTB	CAT-1	Clinical	Lost to follow up
648	Indus Hospital	108	F	17	120	Walk-In	EPTB	CAT-1	Clinical	Still on treatment
649	Indus Hospital	156	F	59	159	Walk-In	EPTB	CAT-1	Clinical	Transfer out
650	Indus Hospital	60	M	17.5	108	Walk-In	EPTB	CAT-1	Clinical	Still on treatment
651	Indus Hospital	84	M	15	107	Walk-In	PTB	CAT-1	Clinical	Treatment Complete
652	Indus Hospital	36	F	11.5	91	Walk-In	EPTB	CAT-1	Clinical	Still on treatment
653	Indus Hospital	156	F	24	132	Walk-In	EPTB	CAT-1	Clinical	Still on treatment
654	Indus Hospital	36	M	13	94	Walk-In	EPTB	CAT-1	Clinical	Still on treatment
655	Indus Hospital	96	F	8	16	Walk-In	PTB	CAT-1	Clinical	Treatment Complete

656	Indus Hospital	120	M	23	131	Walk-In	PTB	CAT-1	Clinical	Treatment Complete
657	Indus Hospital	144	F	17	120	Walk-In	PTB	CAT-1	B+	Still on treatment
658	Indus Hospital	36	M	4	55	Walk-In	PTB	CAT-1	Clinical	Treatment Complete
659	Indus Hospital	96	F	17	106	Walk-In	EPTB	CAT-2	Clinical	Treatment Complete
660	Indus Hospital	120	F	29	143	Walk-In	PTB,EPTB	CAT-1	Clinical	Still on treatment
661	Indus Hospital	144	F	24	141	Walk-In	PTB	CAT-1	B+	Treatment Complete
662	Indus Hospital	84	F	23	130	Walk-In	PTB	CAT-1	Clinical	Treatment Complete
663	Indus Hospital	96	M	17	112	Walk-In	EPTB	CAT-1	Clinical	Lost to follow up
664	Indus Hospital	84	F	20	127	Walk-In	EPTB	CAT-1	Clinical	Still on treatment
665	Indus Hospital	120	F	16	127	Walk-In	EPTB	CAT-1	Clinical	Lost to follow up
666	Indus Hospital	132	M	16	33.3	Walk-In	EPTB	CAT-1	Clinical	Still on treatment
667	Indus Hospital	12	F	7.5	64	Walk-In	EPTB	CAT-1	Clinical	Still on treatment
668	Indus Hospital	84	M	23	130	Walk-In	PTB	CAT-2	Clinical	Died
669	Indus Hospital	144	F	26	142	Walk-In	EPTB	CAT-1	Clinical	Died
670	Indus Hospital	72	F	13.3	96	Walk-In	PTB	CAT-1	Clinical	Treatment Complete
671	Indus Hospital	132	F	26	140	Walk-In	PTB	CAT-1	Clinical	Treatment Complete
672	Indus Hospital	108	F	17	118	Walk-In	EPTB	CAT-1	Clinical	Still on treatment
673	Indus Hospital	156	F	22	135	Walk-In	PTB	CAT-1	Clinical	Treatment Complete
674	Indus Hospital	168	F	30	153	Walk-In	PTB	CAT-1	B+	failure
675	Indus Hospital	132	F	16-9	119	Walk-In	PTB	CAT-1	Clinical	Still on treatment
676	SGH Korangi	24	F	8	82.5	Screening	PTB	CAT-1	Clinical	Treatment Complete
677	SGH Korangi	156	F	N/A	N/A	Screening	PTB	N/A	B+	Transfer out
678	SGH Korangi	120	F	N/A	N/A	Screening	PTB	N/A	Clinical	Transfer out
679	SGH Korangi	12	F	N/A	N/A	Screening	PTB	N/A	Clinical	Transfer out
680	SGH Korangi	132	F	18	11	Screening	PTB	N/A	Clinical	Cure
681	SGH Korangi	96	F	18	N/A	Screening	EPTB	CAT-1	Clinical	Treatment Complete
682	SGH Korangi	96	F	19	109	Screening	PTB	CAT-1	Clinical	Treatment Complete
683	SGH Korangi	120	M	23	125	Screening	EPTB	CAT-1	Clinical	Cure
684	SGH Korangi	48	F	12.1	101.5	Screening	PTB	CAT-1	Clinical	Treatment Complete
685	SGH Korangi	120	F	30	134	Referral	EPTB	CAT-1	Clinical	Treatment Complete
686	SGH Korangi	84	F	17	112	Screening	PTB	CAT-1	Clinical	Cure
687	SGH Korangi	12	F	8.5	N/A	Screening	PTB	CAT-2	Clinical	Cure
688	SGH Korangi	120	F	20	N/A	Screening	PTB	CAT-1	B+	Cure
689	SGH Korangi	48	M	14	N/A	Screening	PTB	CAT-1	Clinical	Treatment Complete
690	SGH Korangi	48	F	14	95	Screening	PTB	CAT-1	Clinical	Treatment Complete
691	SGH Korangi	24	M	10	83	Screening	PTB	CAT-1	Clinical	Treatment Complete
692	SGH Korangi	60	M	14	N/A	Screening	PTB	CAT-1	Clinical	Treatment Complete
693	SGH Korangi	132	F	23	131	Referral	PTB	CAT-1	B+	Treatment Complete
694	SGH Korangi	36	F	14	90	Screening	PTB	CAT-1	Clinical	Treatment Complete
695	SGH Korangi	168	F	26	134	Referral	PTB	CAT-1	B+	Treatment Complete
696	SGH Korangi	168	F	32.8	146	Screening	PTB	CAT-1	Clinical	Treatment Complete

697	SGH Korangi	120	F	25	135	Screening	PTB	CAT-1	B+	Cure
698	SGH Korangi	72	F	15	109	Screening	PTB	CAT-1	Clinical	Treatment Complete
699	SGH Korangi	48	F	12	90	Referral	PTB	CAT-1	Clinical	Treatment Complete
700	SGH Korangi	12	F	9	73	Referral	EPTB	CAT-1	Clinical	Transfer out
701	SGH Korangi	132	F	31.3	141	Screening	PTB	CAT-1	B+	Cure
702	SGH Korangi	144	M	29.9	139	Screening	PTB,EPTB	CAT-1	Clinical	Treatment Complete
703	SGH Korangi	36	M	9.8	78	Referral	PTB	CAT-1	Clinical	Treatment Complete
704	SGH Korangi	26.4	M	8.5	75	Screening	PTB	CAT-1	Clinical	Treatment Complete
705	SGH Korangi	72	F	15.7	107	Screening	PTB	CAT-1	Clinical	Treatment Complete
706	SGH Korangi	96	F	16.9	108	Referral	PTB	CAT-1	Clinical	Treatment Complete
707	SGH Korangi	120	F	25.3	132	Screening	PTB	CAT-1	Clinical	Treatment Complete
708	SGH Korangi	84	F	16.6	108.5	Screening	PTB	CAT-1	Clinical	Treatment Complete
709	SGH Korangi	156	F	34.5	134	Referral	PTB	CAT-1	Clinical	Treatment Complete
710	SGH Korangi	48	F	10.8	87.5	Referral	EPTB	CAT-1	Clinical	Treatment Complete
711	SGH Korangi	168	F	35.5	148	Referral	PTB	CAT-1	B+	Treatment Complete
712	SGH Korangi	132	M	26	130	Screening	PTB	CAT-1	Clinical	Treatment Complete
713	SGH Korangi	132	F	23	134	Screening	PTB	CAT-1	Clinical	Treatment Complete
714	SGH Korangi	156	M	11	23.3	Screening	PTB	CAT-1	Clinical	Treatment Complete
715	SGH Korangi	156	M	33	135	Screening	PTB	CAT-1	Clinical	Treatment Complete
716	SGH Korangi	144	M	36.7	146	Screening	PTB	CAT-1	Clinical	Treatment Complete
717	SGH Korangi	84	M	15	110	Screening	PTB	CAT-1	Clinical	Treatment Complete
718	SGH Korangi	120	F	31.5	132	Screening	PTB	CAT-1	Clinical	Treatment Complete
719	SGH Korangi	132	M	30	132	Screening	PTB	CAT-1	Clinical	Treatment Complete
720	SGH Korangi	120	M	20	111	Screening	PTB	CAT-1	Clinical	Treatment Complete
721	SGH Korangi	84	M	17.4	116	Screening	PTB	CAT-1	Clinical	Treatment Complete
722	SGH Korangi	120	F	20.5	115	Screening	PTB	CAT-1	Clinical	Treatment Complete
723	SGH Korangi	60	M	15.4	95	Screening	PTB	CAT-1	Clinical	Treatment Complete
724	SGH Korangi	12	M	6	68	Screening	PTB	CAT-1	Clinical	Treatment Complete
725	SGH Korangi	156	F	32.7	145	Screening	PTB	CAT-1	Clinical	Treatment Complete
726	SGH Korangi	132	F	33.5	142	Screening	PTB	CAT-1	Clinical	Treatment Complete
727	SGH Korangi	108	F	24.5	129	Screening	PTB	CAT-1	Clinical	Treatment Complete
728	SGH Korangi	12	M	10.5	81	Screening	PTB	CAT-1	Clinical	Treatment Complete
729	SGH Korangi	84	M	13.6	101	Screening	PTB	CAT-1	Clinical	Treatment Complete
730	SGH Korangi	108	F	15.4	110	Screening	PTB	CAT-1	Clinical	Treatment Complete
731	SGH Korangi	120	F	22.3	125	Screening	PTB	CAT-1	Clinical	Treatment Complete
732	SGH Korangi	72	M	15	100	Screening	PTB	CAT-1	Clinical	Treatment Complete
733	SGH Korangi	36	M	13	85	Screening	PTB	CAT-1	Clinical	Treatment Complete
734	SGH Korangi	60	F	13.5	99	Screening	PTB	CAT-1	Clinical	Treatment Complete
735	SGH Korangi	96	M	21	123	Screening	PTB	CAT-1	Clinical	Treatment Complete
736	SGH Korangi	72	M	18	113	Screening	PTB	CAT-1	Clinical	Treatment Complete
737	SGH Korangi	108	F	17.3	107	Screening	PTB	CAT-1	Clinical	Treatment Complete

738	SGH Korangi	72	M	17	100	Screening	PTB	CAT-1	Clinical	Treatment Complete
739	SGH Korangi	96	F	22	117	Screening	PTB	CAT-1	Clinical	Treatment Complete
740	SGH Korangi	108	F	7.5	70	Screening	PTB	CAT-1	Clinical	Treatment Complete
741	SGH Korangi	84	M	16.7	155	Screening	PTB	CAT-1	Clinical	Treatment Complete
742	SGH Korangi	96	M	N/A	N/A	Referral	PTB	CAT-1	Clinical	Treatment Complete
743	SGH Korangi	84	M	17.2	109	Screening	PTB	CAT-1	Clinical	Treatment Complete
744	SGH Korangi	72	F	13.3	94	Screening	PTB	CAT-1	Clinical	Treatment Complete
745	SGH Korangi	18	F	7.6	70	Screening	PTB	CAT-1	Clinical	Treatment Complete
746	SGH Korangi	72	F	13.7	94	Screening	PTB	CAT-1	Clinical	Treatment Complete
747	SGH Korangi	120	F	5.3	60	Screening	PTB	CAT-1	Clinical	Treatment Complete
748	SGH Korangi	60	M	16.5	N/A	Referral	PTB	CAT-1	Clinical	Treatment Complete
749	SGH Korangi	60	M	15.3	97	Screening	PTB	CAT-1	Clinical	Treatment Complete
750	SGH Korangi	18	F	7	70	Screening	PTB	CAT-1	Clinical	Treatment Complete
751	SGH Korangi	72	M	18.5	110	Screening	PTB	CAT-1	Clinical	Treatment Complete
752	SGH Korangi	36	F	10.8	82	Screening	PTB	CAT-1	Clinical	Treatment Complete
753	SGH Korangi	48	M	14	101	Screening	PTB	CAT-1	Clinical	Treatment Complete
754	SGH Korangi	96	F	21.6	117	Screening	PTB	CAT-1	Clinical	Treatment Complete
755	SGH Korangi	84	F	22.5	116	Screening	PTB	CAT-1	Clinical	Treatment Complete
756	SGH Korangi	60	F	17	100	Screening	PTB	CAT-1	Clinical	Treatment Complete
757	SGH Korangi	36	F	13.2	91	Screening	PTB	CAT-1	Clinical	Treatment Complete
758	SGH Korangi	60	F	12.5	91	Screening	PTB	CAT-1	Clinical	Treatment Complete
759	SGH Korangi	48	M	15.8	97.5	Screening	PTB	CAT-1	Clinical	Treatment Complete
760	SGH Korangi	84	M	17.3	112	Screening	PTB	CAT-1	Clinical	Treatment Complete
761	SGH Korangi	36	F	134	23.5	Screening	PTB	CAT-1	Clinical	Treatment Complete
762	SGH Korangi	72	M	14.8	102	Screening	PTB	CAT-1	Clinical	Treatment Complete
763	SGH Korangi	84	F	17.9	115	Screening	PTB	CAT-1	Clinical	Treatment Complete
764	SGH Korangi	18	M	N/A	N/A	Screening	PTB	N/A	Clinical	Treatment Complete
765	SGH Korangi	72	M	16.5	110	Screening	PTB	CAT-1	Clinical	Treatment Complete
766	SGH Korangi	84	F	15	99	Screening	PTB	CAT-1	Clinical	Treatment Complete
767	SGH Korangi	84	M	17.6	113	Screening	PTB	CAT-1	Clinical	Treatment Complete
768	SGH Korangi	60	M	16	109	Screening	PTB	CAT-1	Clinical	Treatment Complete
769	SGH Korangi	24	F	9.4	80	Screening	PTB	CAT-1	Clinical	Treatment Complete
770	SGH Korangi	120	M	20	N/A	Screening	PTB	CAT-1	Clinical	Treatment Complete
771	SGH Korangi	72	M	15	100	Screening	PTB	CAT-1	Clinical	Treatment Complete
772	SGH Korangi	60	M	14.6	96	Screening	PTB	CAT-1	Clinical	Treatment Complete
773	SGH Korangi	72	F	17.9	102	Screening	PTB	CAT-1	Clinical	Treatment Complete
774	SGH Korangi	120	F	18	112	Screening	PTB	CAT-1	Clinical	Treatment Complete
775	SGH Korangi	36	F	12.5	82	Screening	PTB	CAT-1	Clinical	Treatment Complete
776	SGH Korangi	60	M	13.9	100	Screening	PTB	CAT-1	Clinical	Treatment Complete
777	SGH Korangi	120	F	16	122	Referral	PTB	CAT-1	Clinical	Treatment Complete
778	SGH Korangi	72	M	16	109	Referral	PTB	CAT-1	Clinical	Treatment Complete

779	SGH Korangi	120	F	134	23.5	Screening	PTB	CAT-1	Clinical	Treatment Complete
780	SGH Korangi	84	F	17.1	106	Screening	PTB	CAT-1	Clinical	Treatment Complete
781	SGH Korangi	132	F	23.4	137	Screening	PTB	CAT-1	Clinical	Treatment Complete
782	SGH Korangi	60	M	14	99	Screening	PTB	CAT-1	Clinical	Treatment Complete
783	SGH Korangi	36	F	10	80	Screening	PTB	CAT-1	Clinical	Treatment Complete
784	SGH Korangi	84	M	16	112	Screening	PTB	CAT-1	Clinical	Treatment Complete
785	SGH Korangi	168	F	34.2	143	Screening	PTB	CAT-1	Clinical	Cure
786	SGH Korangi	60	M	15	98	Screening	PTB	CAT-1	Clinical	Treatment Complete
787	SGH Korangi	96	M	22.8	117	Referral	PTB	CAT-1	Clinical	Treatment Complete
788	SGH New Karachi	39.6	F	3.3	81	Screening	PTB,EPTB	CAT-1	Clinical	Transfer out
789	SGH New Karachi	84	M	15	111	Screening	PTB	CAT-1	Clinical	Treatment Complete
790	SGH New Karachi	132	F	28	139	Referral	PTB	CAT-1	Clinical	Treatment Complete
791	SGH New Karachi	84	F	23.5	121	Referral	PTB	CAT-1	B+	Treatment Complete
792	SGH New Karachi	12	M	7.5	65	Screening	PTB	CAT-1	Clinical	Treatment Complete
793	SGH New Karachi	132	F	31.5	126	Referral	EPTB	CAT-1	Clinical	Treatment Complete
794	SGH New Karachi	15.6	M	8.2	70	Referral	PTB	CAT-1	Clinical	Treatment Complete
795	SGH New Karachi	120	F	24	130	Screening	PTB	CAT-1	Clinical	Treatment Complete
796	SGH New Karachi	156	M	39	140	Referral	PTB,EPTB	CAT-1	Clinical	Treatment Complete
797	SGH New Karachi	156	F	35	153	Screening	PTB	CAT-1	B+	Cure
798	SGH New Karachi	108	F	21	110	Referral	PTB	CAT-1	Clinical	Treatment Complete
799	SGH New Karachi	168	M	27	140	Referral	PTB	CAT-1	Clinical	Treatment Complete
800	SGH New Karachi	132	F	25	N/A	Screening	PTB	CAT-1	Clinical	Treatment Complete
801	SGH New Karachi	96	M	20.5	N/A	Screening	PTB	CAT-1	Clinical	Treatment Complete
802	SGH New Karachi	24	F	9.5	70	Referral	EPTB	CAT-1	Clinical	Lost to follow up
803	SGH New Karachi	168	F	25	N/A	Referral	PTB	CAT-1	B+	Cure
804	SGH New Karachi	96	M	11	100.8	Screening	EPTB	CAT-1	Clinical	Lost to follow up
805	SGH New Karachi	120	F	18	120	Referral	PTB	CAT-1	Clinical	Treatment Complete
806	SGH New Karachi	84	M	22	110	Referral	PTB	CAT-1	Clinical	Treatment Complete
807	SGH New Karachi	144	F	25	140	Screening	PTB	CAT-1	Clinical	Treatment Complete
808	SGH New Karachi	72	M	11	99	Referral	PTB	CAT-1	B+	Cure
809	SGH New Karachi	144	M	23.5	130	Referral	EPTB	CAT-2	Clinical	Still on treatment
810	SGH New Karachi	132	F	25	N/A	Referral	EPTB	CAT-2	B+	Treatment Complete
811	SGH New Karachi	120	M	25	141	Referral	PTB	CAT-1	B+	Cure
812	SGH New Karachi	120	F	31	140.3	N/A	EPTB	CAT-1	B+	Lost to follow up
813	SGH New Karachi	132	F	6.9	75	Referral	PTB	CAT-1	Clinical	Treatment Complete
814	SGH New Karachi	144	M	23	N/A	Referral	PTB	CAT-1	B+	Lost to follow up
815	SGH New Karachi	60	F	12	90	Referral	EPTB	CAT-1	Clinical	Treatment Complete
816	SGH New Karachi	42	M	12	N/A	Referral	PTB	CAT-1	Clinical	Treatment Complete
817	SGH New Karachi	84	F	14	110	Referral	PTB	CAT-1	Clinical	Treatment Complete
818	SGH New Karachi	24	M	10	70.5	Screening	EPTB	CAT-1	B+	Treatment Complete
819	SGH New Karachi	30	M	9	75	Screening	PTB	CAT-1	Clinical	Treatment Complete

820	SGH New Karachi	54	F	14	95	Referral	PTB	CAT-1	Clinical	Treatment Complete
821	SGH New Karachi	96	M	15.5	110	Referral	EPTB	CAT-1	Clinical	Treatment Complete
822	SGH New Karachi	132	M	25	N/A	Referral	PTB,EPTB	CAT-1	Clinical	Treatment Complete
823	SGH New Karachi	144	M	19	120.8	Screening	PTB	CAT-1	Clinical	Treatment Complete
824	SGH New Karachi	132	M	23.5	133	Screening	PTB	CAT-1	Clinical	Treatment Complete
825	SGH New Karachi	42	M	13.9	80	Referral	EPTB	CAT-1	Clinical	Treatment Complete
826	SGH New Karachi	96	F	13	100.4	Referral	EPTB	CAT-1	Clinical	Lost to follow up
827	SGH New Karachi	96	F	13	100.4	Referral	EPTB	CAT-1	Clinical	Lost to follow up
828	SGH New Karachi	78	M	14	N/A	Referral	EPTB	CAT-1	Clinical	Treatment Complete
829	SGH New Karachi	84	M	17.8	110	Referral	PTB,EPTB	CAT-1	Clinical	Treatment Complete
830	SGH New Karachi	84	F	16	100.7	Referral	PTB	CAT-1	Clinical	Treatment Complete
831	SGH New Karachi	168	F	32	140.5	Screening	PTB	CAT-1	B+	Cure
832	SGH New Karachi	60	M	12.9	N/A	Referral	PTB,EPTB	CAT-1	Clinical	Treatment Complete
833	SGH New Karachi	108	F	21	120.7	Screening	PTB	CAT-1	B+	Lost to follow up
834	SGH New Karachi	156	F	34	N/A	Referral	EPTB	CAT-1	Clinical	Treatment Complete
835	SGH New Karachi	30	F	11	2.5	Referral	EPTB	CAT-1	B+	Treatment Complete
836	SGH New Karachi	120	M	17	120.2	Screening	PTB	CAT-2	B+	Lost to follow up
837	SGH New Karachi	132	F	28	122	Referral	EPTB	CAT-2	B+	Treatment Complete
838	SGH New Karachi	84	M	14	110.6	Referral	PTB,EPTB	CAT-1	Clinical	Treatment Complete
839	SGH New Karachi	72	M	16	100.6	Referral	PTB	CAT-1	Clinical	Treatment Complete
840	SGH New Karachi	132	F	23	N/A	Screening	PTB,EPTB	CAT-1	Clinical	Treatment Complete
841	SGH New Karachi	132	F	14.5	120	Screening	PTB,EPTB	CAT-1	Clinical	Treatment Complete
842	SGH New Karachi	36	F	11	80.4	Referral	PTB	CAT-1	Clinical	Treatment Complete
843	SGH New Karachi	96	F	18	110.4	Referral	PTB	CAT-1	Clinical	Treatment Complete
844	SGH New Karachi	108	F	19	120	Screening	PTB	CAT-1	Clinical	Treatment Complete
845	SGH New Karachi	18	M	7.5	60.8	Screening	PTB	CAT-1	Clinical	Treatment Complete
846	SGH New Karachi	108	M	19	121	Screening	PTB	CAT-1	Clinical	Treatment Complete
847	SGH New Karachi	144	M	33.9	132	Screening	PTB,EPTB	CAT-1	Clinical	Still on treatment
848	SGH New Karachi	96	M	21	111	Screening	PTB	CAT-1	Clinical	Treatment Complete
849	SGH New Karachi	84	M	21.8	111	Screening	EPTB	CAT-1	Clinical	Treatment Complete
850	SGH New Karachi	120	F	32	121	Screening	PTB	CAT-1	Clinical	Lost to follow up
851	SGH New Karachi	120	F	24.5	130	Screening	PTB	CAT-1	Clinical	Treatment Complete
852	SGH New Karachi	30	M	11	80	Screening	PTB	CAT-1	Clinical	Treatment Complete
853	SGH New Karachi	24	F	7.5	79	Screening	PTB	CAT-1	Clinical	Treatment Complete
854	SGH New Karachi	78	M	14.5	120	Referral	PTB	CAT-1	B+	Treatment Complete
855	SGH New Karachi	108	F	20.5	120	Referral	PTB	CAT-1	Clinical	Treatment Complete
856	SGH New Karachi	30	M	7.7	85	Screening	PTB	CAT-1	Clinical	Treatment Complete
857	SGH New Karachi	144	F	28.5	141	Screening	PTB	CAT-1	Clinical	Treatment Complete
858	SGH New Karachi	96	F	21	130	Screening	EPTB	CAT-1	Clinical	Treatment Complete
859	SGH New Karachi	120	F	30	130	Screening	EPTB	CAT-1	Clinical	Treatment Complete
860	SGH New Karachi	108	M	21	121	Referral	PTB	CAT-1	Clinical	Treatment Complete

861	SGH New Karachi	24	M	9	71	Screening	PTB	CAT-1	Clinical	Treatment Complete
862	SGH New Karachi	120	M	27.5	130	Screening	PTB	CAT-1	Clinical	Treatment Complete
863	SGH New Karachi	60	F	18.5	71	Screening	PTB	CAT-1	Clinical	Treatment Complete
864	SGH New Karachi	60	F	18.5	71	Screening	PTB	CAT-1	Clinical	Treatment Complete
865	SGH New Karachi	144	M	28	130	Referral	PTB	CAT-1	Clinical	Treatment Complete
866	SGH New Karachi	60	M	13.5	90	Transfer In	PTB	CAT-1	Clinical	Treatment Complete
867	SGH New Karachi	48	M	11.5	89	Screening	EPTB	CAT-1	Clinical	Lost to follow up
868	SGH New Karachi	48	F	12.5	98	Referral	PTB	CAT-1	Clinical	Treatment Complete
869	SGH New Karachi	84	M	18	119	Referral	PTB	CAT-1	Clinical	Treatment Complete
870	SGH New Karachi	60	M	16	100.3	Referral	EPTB	CAT-1	Clinical	Treatment Complete
871	SGH New Karachi	30	F	11.5	87	Referral	PTB	CAT-1	Clinical	Treatment Complete
872	SGH New Karachi	72	F	13	93	Screening	PTB	CAT-1	Clinical	Treatment Complete
873	SGH New Karachi	48	F	11.5	90	Referral	PTB,EPTB	CAT-1	Clinical	Treatment Complete
874	SGH New Karachi	36	F	8	71	Referral	EPTB	CAT-1	Clinical	Lost to follow up
875	SGH New Karachi	96	F	16	133	Referral	PTB	CAT-1	B+	Treatment Complete
876	SGH New Karachi	108	F	24	120.3	Referral	EPTB	CAT-1	Clinical	Treatment Complete
877	SGH New Karachi	120	F	30	142	Referral	PTB	CAT-1	B+	Treatment Complete
878	SGH New Karachi	132	F	28	103	Referral	EPTB	CAT-1	Clinical	Treatment Complete
879	SGH New Karachi	132	F	29.5	130	Referral	EPTB	CAT-1	Clinical	Treatment Complete
880	SGH New Karachi	108	M	21	121	Referral	PTB	CAT-1	Clinical	Treatment Complete
881	SGH New Karachi	84	M	20	119	Screening	PTB	CAT-1	B+	Treatment Complete
882	SGH New Karachi	12	M	6.5	66	Screening	PTB	CAT-1	B+	Treatment Complete
883	SGH New Karachi	36	F	13	N/A	Transfer In	EPTB	CAT-1	Clinical	Treatment Complete
884	SGH Liaquatabad	24	M	7	68	Screening	PTB	CAT-1	Clinical	Treatment Complete
885	SGH Liaquatabad	36	F	9	86	Screening	PTB	CAT-1	Clinical	Treatment Complete
886	SGH Liaquatabad	60	F	15	142	Screening	PTB	CAT-1	Clinical	Treatment Complete
887	SGH Liaquatabad	120	F	21	121	Screening	EPTB	CAT-1	Clinical	Treatment Complete
888	SGH Liaquatabad	156	F	27	142	Screening	EPTB	CAT-1	Clinical	Treatment Complete
889	SGH Liaquatabad	84	M	17	105.5	Screening	PTB	CAT-1	Clinical	Treatment Complete
890	SGH Liaquatabad	84	F	N/A	N/A	Screening	PTB	CAT-1	Clinical	Treatment Complete
891	SGH Liaquatabad	96	M	N/A	N/A	Screening	PTB	CAT-1	Clinical	Treatment Complete
892	SGH Liaquatabad	96	M	19	120	Screening	EPTB	CAT-2	Clinical	Treatment Complete
893	SGH Liaquatabad	108	F	N/A	N/A	Screening	PTB	CAT-1	Clinical	Treatment Complete
894	SGH Liaquatabad	132	F	27	120	Screening	PTB	CAT-1	Clinical	Treatment Complete
895	SGH Liaquatabad	120	M	N/A	N/A	Screening	PTB	CAT-2	Clinical	Treatment Complete
896	SGH Liaquatabad	24	F	7.5	70	Screening	PTB	CAT-1	Clinical	Treatment Complete
897	SGH Liaquatabad	108	F	N/A	N/A	Screening	PTB	CAT-1	Clinical	Treatment Complete
898	SGH Liaquatabad	36	F	11.5	95	Screening	PTB	CAT-1	Clinical	Treatment Complete
899	SGH Liaquatabad	48	M	14	142	Screening	PTB	CAT-1	Clinical	Treatment Complete
900	SGH Liaquatabad	84	M	18	N/A	Screening	PTB	CAT-1	Clinical	Treatment Complete
901	SGH Liaquatabad	18	M	5.5	151	Screening	PTB	CAT-1	Clinical	Treatment Complete

902	SGH Liaquatabad	84	F	16	107	Screening	PTB	CAT-1	Clinical	Lost to follow up
903	SGH Liaquatabad	36	F	10	85	Referral	EPTB	CAT-1	Clinical	Treatment Complete
904	SGH Liaquatabad	132	F	21	130	Screening	PTB	CAT-1	Clinical	Treatment Complete
905	SGH Liaquatabad	96	F	25	131	Screening	PTB	CAT-1	Clinical	Treatment Complete
906	SGH Liaquatabad	96	M	17	111	Screening	PTB	CAT-1	Clinical	Treatment Complete
907	SGH Liaquatabad	48	F	9	90	Screening	PTB	CAT-1	Clinical	Treatment Complete
908	SGH Liaquatabad	144	F	28	N/A	Screening	EPTB	CAT-1	Clinical	Treatment Complete
909	SGH Liaquatabad	84	F	26	144	Screening	EPTB	CAT-1	Clinical	Treatment Complete
910	SGH Liaquatabad	96	M	17	112	Screening	PTB	CAT-1	Clinical	Treatment Complete
911	SGH Liaquatabad	24	F	13	103	Screening	PTB	CAT-2	Clinical	Treatment Complete
912	SGH Liaquatabad	54	F	10	92	Screening	PTB	CAT-1	Clinical	Treatment Complete
913	SGH Liaquatabad	16.8	M	9.5	89	Screening	PTB	CAT-1	Clinical	Treatment Complete
914	SGH Liaquatabad	15.6	F	5	57	Screening	PTB	CAT-1	Clinical	Treatment Complete
915	SGH Liaquatabad	36	M	8.4	75	Screening	PTB	CAT-1	Clinical	Treatment Complete
916	SGH Liaquatabad	36	F	8	72	Screening	PTB	CAT-1	Clinical	Cure
917	SGH Liaquatabad	108	F	20.6	128	Screening	PTB	CAT-1	Clinical	Treatment Complete
918	SGH Liaquatabad	36	M	12.3	87.5	Screening	PTB	CAT-1	Clinical	Treatment Complete
919	SGH Liaquatabad	72	M	15	103	Screening	PTB	CAT-1	Clinical	Treatment Complete
920	SGH Liaquatabad	54	F	12	89	Screening	PTB	CAT-1	Clinical	Treatment Complete
921	SGH Liaquatabad	84	F	20	119	Screening	PTB	CAT-1	Clinical	Lost to follow up
922	SGH Liaquatabad	144	F	26	141	Screening	EPTB	CAT-1	Clinical	Treatment Complete
923	SGH Liaquatabad	108	F	15	115	Screening	EPTB	CAT-1	Clinical	Treatment Complete
924	SGH Liaquatabad	60	M	16.5	110	Screening	PTB	CAT-1	Clinical	Lost to follow up
925	SGH Liaquatabad	66	M	14	102	Screening	PTB	CAT-1	Clinical	Treatment Complete
926	SGH Liaquatabad	60	M	12	98	Screening	PTB	CAT-1	Clinical	Treatment Complete
927	SGH Liaquatabad	48	M	10.4	84	Screening	PTB	CAT-1	Clinical	Treatment Complete
928	SGH Liaquatabad	144	M	33.5	142	Screening	EPTB	CAT-1	Clinical	Treatment Complete
929	SGH Liaquatabad	24	M	5	70	Screening	PTB	CAT-1	Clinical	Cure
930	SGH Liaquatabad	90	F	18	119	Screening	PTB	CAT-1	Clinical	Treatment Complete
931	SGH Liaquatabad	24	M	7	73	Screening	PTB	CAT-1	Clinical	Lost to follow up
932	SGH Liaquatabad	144	F	27.4	137	Screening	PTB	CAT-1	Clinical	Treatment Complete
933	SGH Liaquatabad	36	F	11.2	90	Referral	PTB	CAT-1	B+	Treatment Complete
934	SGH Liaquatabad	84	M	15.5	116	Screening	PTB	CAT-1	Clinical	Treatment Complete
935	SGH Liaquatabad	120	M	18.2	125	Screening	PTB	CAT-1	Clinical	Treatment Complete
936	SGH Liaquatabad	84	F	12.7	99	Screening	PTB	CAT-1	Clinical	Treatment Complete
937	SGH Liaquatabad	144	F	16.1	117	Screening	PTB	CAT-1	Clinical	Treatment Complete
938	SGH Liaquatabad	12	M	7.2	67	Screening	EPTB	CAT-1	Clinical	Treatment Complete
939	SGH Liaquatabad	120	F	5.4	65	Screening	PTB	CAT-1	Clinical	Treatment Complete
940	SGH Liaquatabad	48	F	10.8	92.5	Screening	EPTB	CAT-1	Clinical	Treatment Complete
941	SGH Liaquatabad	120	F	5	67	Screening	PTB	CAT-1	Clinical	Treatment Complete
942	SGH Liaquatabad	36	M	8.8	77	Screening	PTB	CAT-1	Clinical	Lost to follow up

943	SGH Liaquatabad	96	M	18.6	117	Screening	PTB	CAT-1	Clinical	Treatment Complete
944	SGH Liaquatabad	96	F	4.6	55	Screening	PTB	CAT-1	Clinical	Lost to follow up
945	SGH Liaquatabad	108	F	23	134	Screening	PTB	CAT-1	Clinical	Treatment Complete
946	SGH Liaquatabad	84	M	22	124	Screening	PTB	CAT-1	Clinical	Treatment Complete
947	SGH Liaquatabad	120	F	5.2	60	Screening	EPTB	CAT-1	Clinical	Treatment Complete
948	SGH Liaquatabad	30	F	6.7	60	Screening	PTB	CAT-2	Clinical	Treatment Complete
949	SGH Liaquatabad	48	M	11.8	100	Screening	PTB	CAT-1	Clinical	Treatment Complete
950	SGH Liaquatabad	156	M	38.7	135	Screening	PTB	CAT-1	Clinical	Treatment Complete
951	SGH Liaquatabad	120	F	15.3	109	Screening	EPTB	CAT-1	Clinical	Treatment Complete
952	SGH Liaquatabad	18	F	4.8	65	Screening	PTB	CAT-1	Clinical	Treatment Complete
953	SGH Liaquatabad	24	M	9.1	80	Screening	PTB	CAT-1	Clinical	Treatment Complete
954	SGH Liaquatabad	120	M	24.5	123	Screening	PTB	CAT-1	Clinical	Treatment Complete
955	SGH Liaquatabad	132	F	23	130	Screening	PTB	CAT-1	Clinical	Treatment Complete
956	SGH Liaquatabad	120	M	21.3	130	Screening	PTB	CAT-1	Clinical	Treatment Complete
957	SGH Liaquatabad	144	M	29.3	140	Screening	PTB	CAT-1	Clinical	Treatment Complete
958	SGH Liaquatabad	132	M	25	135	Screening	PTB	CAT-1	Clinical	Treatment Complete
959	SGH Liaquatabad	36	M	9.5	81	Screening	PTB	CAT-1	Clinical	Treatment Complete
960	SGH Liaquatabad	48	F	15.4	102	Screening	PTB	CAT-1	Clinical	Treatment Complete
961	SGH Liaquatabad	120	F	23.4	130	Screening	PTB	CAT-1	Clinical	Treatment Complete
962	SGH Liaquatabad	16.8	M	8.7	71	Screening	PTB	CAT-1	Clinical	Treatment Complete
963	SGH Liaquatabad	12	F	7.3	76	Screening	PTB	CAT-1	Clinical	Lost to follow up
964	SGH Liaquatabad	120	F	23	127	Screening	PTB	CAT-1	Clinical	Treatment Complete
965	SGH Liaquatabad	72	M	14.2	111	Screening	EPTB	CAT-1	Clinical	Treatment Complete
966	SGH Liaquatabad	96	M	5.8	60	Screening	PTB	CAT-1	Clinical	Treatment Complete
967	SGH Liaquatabad	60	M	20.7	136	Screening	PTB	CAT-1	Clinical	Treatment Complete
968	SGH Liaquatabad	60	F	13.6	96	Screening	PTB	CAT-1	Clinical	Treatment Complete
969	SGH Liaquatabad	12	F	7.7	69	Screening	PTB	CAT-2	Clinical	Treatment Complete
970	SGH Liaquatabad	84	F	15.9	109	Screening	PTB	CAT-3	Clinical	Treatment Complete
971	SGH Liaquatabad	96	M	7	60	Screening	PTB	CAT-1	Clinical	Lost to follow up
972	SGH Liaquatabad	96	F	20	116	Screening	PTB	CAT-1	Clinical	Treatment Complete
973	SGH Liaquatabad	24	M	10	78	Screening	PTB	CAT-1	Clinical	Lost to follow up
974	SGH Liaquatabad	24	F	8.7	98	Screening	PTB	CAT-1	Clinical	Died
975	SGH Liaquatabad	132	F	23.5	132	Screening	PTB	CAT-1	Clinical	Treatment Complete
976	SGH Liaquatabad	132	M	9.5	65	Screening	PTB	CAT-1	Clinical	Treatment Complete
977	SGH Liaquatabad	12	F	7.4	60	Screening	PTB	CAT-1	Clinical	Treatment Complete
978	SGH Liaquatabad	36	F	9	86	Screening	PTB	CAT-1	Clinical	Treatment Complete
979	SGH Liaquatabad	120	M	26.4	136	Screening	PTB	CAT-1	Clinical	Treatment Complete
980	SGH Liaquatabad	84	M	29.8	141	Screening	EPTB	CAT-2	Clinical	Treatment Complete
981	SGH Liaquatabad	48	M	13.8	100	Screening	PTB	CAT-3	Clinical	Treatment Complete
982	SGH Liaquatabad	120	F	19	128	Screening	PTB	CAT-1	Clinical	Treatment Complete
983	SGH Liaquatabad	120	M	17	117	Screening	PTB	CAT-2	Clinical	Treatment Complete

984	SGH Liaquatabad	36	M	13	96	Screening	PTB	CAT-1	Clinical	Treatment Complete
985	SGH Liaquatabad	144	M	25.5	138	Screening	PTB	CAT-1	Clinical	Treatment Complete
986	SGH Liaquatabad	96	F	26	152	Screening	PTB	CAT-1	Clinical	Treatment Complete
987	SGH Liaquatabad	72	M	19	121	Screening	PTB	CAT-1	Clinical	Lost to follow up
988	SGH Liaquatabad	24	F	7.4	74	Screening	PTB	CAT-1	Clinical	Treatment Complete
989	SGH Liaquatabad	24	F	8.2	80	Screening	PTB	CAT-1	Clinical	Treatment Complete
990	SGH Liaquatabad	72	F	8.2	80	Screening	PTB	CAT-1	Clinical	Treatment Complete
991	SGH Liaquatabad	120	M	20.2	128	Screening	PTB	CAT-2	Clinical	Treatment Complete
992	SGH Liaquatabad	24	M	8.1	68	Screening	PTB	CAT-1	Clinical	Treatment Complete
993	SGH Liaquatabad	144	M	27	138	Screening	EPTB	CAT-1	Clinical	Treatment Complete
994	SGH Liaquatabad	18	F	5.8	75	Screening	PTB	CAT-1	Clinical	Lost to follow up
995	SGH Liaquatabad	36	F	5.9	65	Screening	PTB	CAT-1	Clinical	Treatment Complete
996	SGH Liaquatabad	96	M	22.5	117	Screening	PTB	CAT-1	Clinical	Treatment Complete
997	SGH Liaquatabad	84	F	18.7	110	Screening	PTB	CAT-1	Clinical	Treatment Complete
998	SGH Liaquatabad	132	M	31.3	149	Screening	PTB	CAT-1	Clinical	Treatment Complete
999	SGH Liaquatabad	54	M	14.3	104	Screening	EPTB	CAT-1	Clinical	Treatment Complete
1000	SGH Liaquatabad	24	F	9.2	76	Screening	PTB	CAT-1	Clinical	Lost to follow up
1001	SGH Liaquatabad	132	M	23	125	Screening	PTB	CAT-1	Clinical	Lost to follow up
1002	SGH Liaquatabad	96	F	7	75	Screening	PTB	CAT-1	Clinical	Treatment Complete
1003	SGH Liaquatabad	108	F	23	134	Screening	PTB	CAT-1	Clinical	Treatment Complete
1004	SGH Liaquatabad	96	F	19.4	125	Screening	EPTB	CAT-1	Clinical	Treatment Complete
1005	SGH Liaquatabad	18	M	7.2	72	Screening	PTB	CAT-1	Clinical	Treatment Complete
1006	SGH Liaquatabad	60	F	12.5	86	Screening	PTB	CAT-1	Clinical	Treatment Complete
1007	SGH Liaquatabad	48	M	12.7	90	Screening	PTB	CAT-1	Clinical	Lost to follow up
1008	SGH Liaquatabad	12	M	7.5	92	Screening	PTB	CAT-1	Clinical	Treatment Complete
1009	SGH Liaquatabad	84	M	18.7	116	Screening	PTB	CAT-1	Clinical	Treatment Complete
1010	SGH Liaquatabad	96	F	26	130	Screening	PTB	CAT-1	Clinical	Treatment Complete
1011	SGH Liaquatabad	48	M	12.5	93	Screening	PTB	CAT-1	Clinical	Treatment Complete
1012	SGH Liaquatabad	48	M	11.1	86	Screening	PTB	CAT-1	Clinical	Treatment Complete
1013	SGH Liaquatabad	12	M	9	92	Screening	PTB	CAT-1	Clinical	Treatment Complete
1014	SGH Liaquatabad	144	F	31.2	148	Screening	PTB	CAT-1	Clinical	Treatment Complete
1015	SGH Liaquatabad	108	M	19.4	120	Screening	PTB	CAT-1	Clinical	Treatment Complete
1016	SGH Liaquatabad	108	M	27.1	136	Screening	PTB	CAT-1	Clinical	Treatment Complete
1017	SGH Liaquatabad	108	F	23.4	131	Screening	PTB	CAT-1	Clinical	Treatment Complete
1018	SGH Liaquatabad	24	F	8.7	74	Screening	PTB	CAT-1	Clinical	Treatment Complete
1019	SGH Liaquatabad	120	M	24	130	Screening	PTB	CAT-1	Clinical	Treatment Complete
1020	SGH Liaquatabad	84	F	16	117	Screening	PTB	CAT-1	Clinical	Treatment Complete
1021	SGH Liaquatabad	60	F	14.6	102	Screening	PTB	CAT-1	Clinical	Treatment Complete
1022	SGH Liaquatabad	84	M	19	110	Screening	PTB	CAT-1	Clinical	Treatment Complete
1023	SGH Liaquatabad	96	F	24.8	128	Screening	PTB	CAT-1	Clinical	Lost to follow up
1024	SGH Liaquatabad	132	M	18.3	114	Screening	PTB	CAT-2	Clinical	Still on treatment

1025	SGH Liaquatabad	36	F	12	84	Screening	PTB	CAT-1	Clinical	Lost to follow up
1026	SGH Liaquatabad	60	M	14.9	104	Screening	PTB	CAT-2	Clinical	Still on treatment
1027	SGH Liaquatabad	144	M	25.5	139	Screening	PTB	CAT-1	Clinical	Treatment Complete
1028	SGH Liaquatabad	168	F	18.8	130	Screening	PTB	CAT-1	Clinical	Treatment Complete
1029	SGH Liaquatabad	72	M	12	97	Screening	EPTB	CAT-1	Clinical	Treatment Complete
1030	SGH Liaquatabad	72	F	17.5	106	Screening	PTB	CAT-1	Clinical	Treatment Complete
1031	SGH Liaquatabad	N/A	F	6.7	65	Screening	PTB	CAT-1	Clinical	Treatment Complete
1032	SGH Liaquatabad	108	F	14.3	113	Screening	PTB	CAT-1	Clinical	Treatment Complete
1033	SGH Liaquatabad	60	F	13.5	94	Screening	PTB	CAT-1	Clinical	Treatment Complete
1034	SGH Liaquatabad	96	F	13.8	108	Screening	PTB	CAT-1	Clinical	Cure
1035	SGH Liaquatabad	48	M	16	100	Screening	PTB	CAT-1	Clinical	Transfer out
1036	SGH Liaquatabad	180	F	33.2	143	Screening	PTB	CAT-1	Clinical	Treatment Complete
1037	Civil Hospital	84	F	24	N/A	Screening	PTB	CAT-1	Clinical	Treatment Complete
1038	Civil Hospital	12	F	8	N/A	Screening	PTB	CAT-1	Clinical	Treatment Complete
1039	Civil Hospital	72	F	15	N/A	Screening	PTB	CAT-1	Clinical	Treatment Complete
1040	Civil Hospital	84	F	20	N/A	Screening	EPTB	CAT-1	Clinical	Treatment Complete
1041	Civil Hospital	48	F	13	N/A	Screening	EPTB	CAT-1	Clinical	Treatment Complete
1042	Civil Hospital	48	M	12	N/A	Screening	PTB	CAT-1	Clinical	Treatment Complete
1043	Civil Hospital	144	F	28	N/A	Screening	PTB	CAT-1	B+	Treatment Complete
1044	Civil Hospital	120	F	20	N/A	Screening	EPTB	CAT-1	Clinical	Lost to follow up
1045	Civil Hospital	84	F	16	N/A	Screening	PTB,EPTB	CAT-1	Clinical	Treatment Complete
1046	Civil Hospital	96	F	N/A	N/A	Screening	EPTB	CAT-1	Clinical	Treatment Complete
1047	Civil Hospital	60	M	15	N/A	Screening	PTB	CAT-1	Clinical	Treatment Complete
1048	Civil Hospital	108	F	18	N/A	Screening	PTB	CAT-2	Clinical	Treatment Complete
1049	Civil Hospital	60	M	16	N/A	Screening	PTB	CAT-1	Clinical	Treatment Complete
1050	Civil Hospital	84	M	16	N/A	Screening	PTB	CAT-1	Clinical	Lost to follow up
1051	Civil Hospital	96	M	22	N/A	Screening	PTB	CAT-1	Clinical	Treatment Complete
1052	Civil Hospital	144	F	20	N/A	Screening	PTB	CAT-1	Clinical	Treatment Complete
1053	Civil Hospital	96	F	18	N/A	Screening	PTB	CAT-1	Clinical	Treatment Complete
1054	Civil Hospital	96	M	26	N/A	Screening	EPTB	CAT-2	Clinical	Lost to follow up
1055	Civil Hospital	60	F	15	N/A	Screening	PTB	CAT-1	Clinical	Treatment Complete
1056	Civil Hospital	84	F	15	N/A	Screening	PTB,EPTB	CAT-2	Clinical	Lost to follow up
1057	Civil Hospital	60	M	15	N/A	Screening	PTB,EPTB	CAT-1	Clinical	Lost to follow up
1058	Civil Hospital	132	F	N/A	N/A	Screening	PTB	CAT-1	Clinical	Treatment Complete
1059	Civil Hospital	30	F	7	N/A	Screening	PTB,EPTB	CAT-1	Clinical	Treatment Complete
1060	Civil Hospital	48	M	10	N/A	Screening	PTB	CAT-1	Clinical	Lost to follow up
1061	Civil Hospital	108	F	23	N/A	Screening	PTB	CAT-1	Clinical	Treatment Complete
1062	Civil Hospital	144	F	36	N/A	Screening	PTB	CAT-1	Clinical	Transfer out
1063	Civil Hospital	36	F	10	N/A	Screening	PTB	CAT-1	Clinical	Treatment Complete
1064	Civil Hospital	60	F	18	N/A	Screening	PTB	CAT-1	Clinical	Treatment Complete
1065	Civil Hospital	120	M	29	N/A	Screening	EPTB	CAT-1	Clinical	Treatment Complete

1066	Civil Hospital	144	M	19	N/A	Screening	PTB	CAT-1	Clinical	Treatment Complete
1067	Civil Hospital	84	F	13	N/A	Screening	PTB	CAT-1	Clinical	Treatment Complete
1068	Civil Hospital	96	F	19	N/A	Screening	EPTB	CAT-1	Clinical	Treatment Complete
1069	Civil Hospital	120	F	28	N/A	Screening	EPTB	CAT-1	Clinical	Treatment Complete
1070	Civil Hospital	144	M	23	N/A	Screening	EPTB	CAT-1	Clinical	Lost to follow up
1071	Civil Hospital	108	M	21	N/A	Screening	PTB	CAT-1	Clinical	Treatment Complete
1072	Civil Hospital	12	F	8	N/A	Screening	PTB	CAT-1	Clinical	Treatment Complete
1073	Civil Hospital	30	F	10	N/A	Screening	PTB	CAT-1	Clinical	Transfer out
1074	Civil Hospital	120	F	22	N/A	Screening	EPTB	CAT-1	Clinical	Treatment Complete
1075	Civil Hospital	84	F	18	N/A	Screening	PTB	CAT-1	Clinical	Transfer out
1076	Civil Hospital	96	F	18	N/A	Screening	EPTB	CAT-1	Clinical	Treatment Complete
1077	Civil Hospital	132	F	36	N/A	Screening	EPTB	CAT-1	Clinical	Treatment Complete
1078	Civil Hospital	84	F	22	N/A	Screening	EPTB	CAT-1	Clinical	Treatment Complete
1079	Civil Hospital	120	M	5	N/A	Screening	PTB	CAT-1	Clinical	Treatment Complete
1080	Civil Hospital	48	F	12	N/A	Screening	PTB,EPTB	CAT-1	Clinical	Treatment Complete
1081	Civil Hospital	216	M	10	N/A	Screening	PTB	CAT-1	Clinical	Treatment Complete
1082	Civil Hospital	132	F	23	N/A	Screening	PTB,EPTB	CAT-1	Clinical	Treatment Complete
1083	Civil Hospital	24	M	8	N/A	Screening	PTB	CAT-1	Clinical	Treatment Complete
1084	Civil Hospital	108	M	16	N/A	Screening	PTB	CAT-1	Clinical	Treatment Complete
1085	Civil Hospital	60	F	13	N/A	Screening	EPTB	CAT-1	Clinical	Treatment Complete
1086	Civil Hospital	42	M	N/A	N/A	Screening	PTB	N/A	Clinical	Treatment Complete
1087	Civil Hospital	96	F	18	N/A	Screening	PTB,EPTB	CAT-1	B+	Treatment Complete
1088	Civil Hospital	36	M	13	N/A	Screening	PTB	CAT-1	Clinical	Treatment Complete
1089	Civil Hospital	36	F	8.5	N/A	Screening	PTB	CAT-1	Clinical	Treatment Complete
1090	Civil Hospital	96	F	20	N/A	Screening	PTB	CAT-1	Clinical	Treatment Complete
1091	Civil Hospital	36	F	8	N/A	Screening	PTB	CAT-1	Clinical	Treatment Complete
1092	Civil Hospital	24	F	7	N/A	Screening	PTB	CAT-1	Clinical	Treatment Complete
1093	Civil Hospital	120	F	29	N/A	Screening	PTB	CAT-1	B+	Treatment Complete
1094	Civil Hospital	72	F	15	N/A	Screening	PTB	CAT-1	Clinical	Treatment Complete
1095	Civil Hospital	48	F	13	N/A	Screening	PTB	CAT-1	Clinical	Treatment Complete
1096	Civil Hospital	96	M	18	N/A	Screening	PTB	CAT-1	B+	Treatment Complete
1097	Civil Hospital	30	F	8.5	N/A	Screening	PTB	CAT-1	Clinical	Treatment Complete
1098	Civil Hospital	18	F	7.5	N/A	Screening	PTB	CAT-1	B+	Cure
1099	Civil Hospital	24	M	10	N/A	Screening	PTB	CAT-1	Clinical	Treatment Complete
1100	Civil Hospital	36	F	13	N/A	Screening	EPTB	CAT-1	Clinical	Treatment Complete
1101	Civil Hospital	12	F	13	N/A	Screening	PTB	CAT-1	Clinical	Treatment Complete
1102	Civil Hospital	108	F	25	N/A	Screening	PTB	CAT-2	Clinical	Treatment Complete
1103	Civil Hospital	67.2	F	14	N/A	Screening	PTB	CAT-1	Clinical	Treatment Complete
1104	Civil Hospital	132	F	28	N/A	Screening	PTB	CAT-1	Clinical	Treatment Complete
1105	Civil Hospital	36	M	10	N/A	Screening	PTB	CAT-1	Clinical	Treatment Complete
1106	Civil Hospital	60	M	19	N/A	Screening	PTB	CAT-1	Clinical	Treatment Complete

1107	Civil Hospital	168	M	9.3	N/A	Screening	PTB,EPTB	CAT-1	B+	Treatment Complete
1108	Civil Hospital	96	F	16.4	N/A	Screening	PTB	CAT-1	Clinical	Treatment Complete
1109	Civil Hospital	132	F	31	N/A	Screening	PTB,EPTB	CAT-1	Clinical	Treatment Complete
1110	Civil Hospital	120	M	18	N/A	Screening	PTB	CAT-1	Clinical	Treatment Complete
1111	Civil Hospital	84	M	19.5	N/A	Screening	PTB	CAT-1	Clinical	Treatment Complete
1112	Civil Hospital	31.2	M	12	N/A	Screening	PTB	CAT-1	Clinical	Treatment Complete
1113	Civil Hospital	84	F	20	N/A	Screening	PTB	CAT-1	Clinical	Treatment Complete
1114	Civil Hospital	60	M	12	N/A	Screening	PTB	CAT-1	Clinical	Treatment Complete
1115	Civil Hospital	120	F	25	N/A	Screening	PTB	CAT-1	Clinical	Treatment Complete
1116	Civil Hospital	60	F	12	N/A	Screening	PTB	CAT-1	Clinical	Treatment Complete
1117	Civil Hospital	72	M	15	N/A	Screening	PTB	CAT-1	Clinical	Treatment Complete
1118	Civil Hospital	60	F	17	N/A	Screening	PTB	CAT-1	Clinical	Treatment Complete
1119	Civil Hospital	96	F	19	N/A	Screening	PTB	CAT-1	Clinical	Treatment Complete
1120	Civil Hospital	120	M	7.3	N/A	Screening	PTB	CAT-1	Clinical	Lost to follow up
1121	Civil Hospital	120	F	23.5	N/A	Screening	PTB	CAT-1	Clinical	Treatment Complete
1122	Civil Hospital	84	M	15.5	N/A	Screening	EPTB	CAT-1	Clinical	Treatment Complete
1123	Civil Hospital	36	M	11	N/A	Screening	PTB	CAT-1	B+	Treatment Complete
1124	Civil Hospital	84	F	15.9	N/A	Screening	PTB	CAT-1	Clinical	Lost to follow up
1125	Civil Hospital	144	F	24.5	N/A	Screening	EPTB	CAT-1	Clinical	Treatment Complete
1126	Civil Hospital	84	F	19	N/A	Screening	PTB	CAT-1	Clinical	Treatment Complete
1127	Civil Hospital	36	F	11	N/A	Screening	EPTB	CAT-1	Clinical	Treatment Complete
1128	Civil Hospital	36	M	11	N/A	Screening	PTB	CAT-1	Clinical	Treatment Complete
1129	Civil Hospital	72	F	13.8	N/A	Screening	PTB	CAT-1	Clinical	Treatment Complete
1130	Civil Hospital	84	M	19.5	N/A	Screening	PTB	CAT-1	Clinical	Treatment Complete
1131	Civil Hospital	84	F	20	N/A	Screening	PTB	CAT-1	Clinical	Treatment Complete
1132	Civil Hospital	31.2	F	10	N/A	Screening	PTB	CAT-1	Clinical	Treatment Complete
1133	Civil Hospital	48	M	13	N/A	Screening	PTB	CAT-1	Clinical	Treatment Complete
1134	Civil Hospital	120	M	15	N/A	Screening	PTB	CAT-1	Clinical	Treatment Complete
1135	Civil Hospital	144	F	27	N/A	Screening	PTB,EPTB	CAT-1	Clinical	Transfer out
1136	Civil Hospital	120	F	25.4	N/A	Screening	PTB	CAT-1	Clinical	Treatment Complete
1137	Civil Hospital	132	F	24	N/A	Screening	PTB,EPTB	CAT-1	Clinical	Treatment Complete
1138	Civil Hospital	108	F	18.7	N/A	Screening	PTB,EPTB	CAT-1	Clinical	Treatment Complete
1139	Civil Hospital	132	F	24	N/A	Screening	EPTB	CAT-1	Clinical	Treatment Complete
1140	Civil Hospital	120	F	27	N/A	Screening	PTB,EPTB	CAT-1	Clinical	Treatment Complete
1141	Civil Hospital	60	M	7.3	N/A	Screening	PTB	CAT-1	Clinical	Treatment Complete
1142	Civil Hospital	96	M	24	N/A	Screening	PTB	CAT-1	Clinical	Lost to follow up
1143	Civil Hospital	96	F	21	N/A	Screening	PTB	CAT-1	Clinical	Treatment Complete
1144	Civil Hospital	24	M	9	83	Screening	PTB	CAT-1	Clinical	Treatment Complete
1145	Civil Hospital	108	F	22	N/A	Screening	PTB	CAT-1	Clinical	Treatment Complete
1146	Civil Hospital	84	F	18	N/A	Screening	PTB,EPTB	CAT-1	Clinical	Treatment Complete
1147	Civil Hospital	48	F	16	N/A	Screening	PTB	CAT-1	Clinical	Treatment Complete

1148	Civil Hospital	120	M	19.9	N/A	Screening	PTB	CAT-1	Clinical	Treatment Complete
1149	Civil Hospital	72	M	14	N/A	Screening	PTB,EPTB	CAT-1	Clinical	Treatment Complete
1150	Civil Hospital	27.6	M	8.3	78	Screening	PTB	CAT-1	Clinical	Treatment Complete
1151	Civil Hospital	72	F	18.4	118	Screening	PTB	CAT-1	Clinical	Treatment Complete
1152	Civil Hospital	24	M	12.3	86	Screening	PTB	CAT-1	Clinical	Treatment Complete
1153	Civil Hospital	96	F	6.5	N/A	Screening	PTB	CAT-1	Clinical	Treatment Complete
1154	Civil Hospital	24	F	8.8	80	Referral	PTB	CAT-1	Clinical	Treatment Complete
1155	Civil Hospital	72	M	14.7	115	Screening	PTB	CAT-1	Clinical	Treatment Complete
1156	Civil Hospital	144	F	13.2	115	Referral	EPTB	CAT-1	Clinical	Treatment Complete
1157	Civil Hospital	108	F	16.9	N/A	Referral	EPTB	CAT-1	Clinical	Treatment Complete
1158	Civil Hospital	27.6	M	10.5	86	Referral	PTB	CAT-1	Clinical	Treatment Complete
1159	Civil Hospital	132	F	23	131	Screening	PTB	CAT-1	Clinical	Treatment Complete
1160	Civil Hospital	60	F	13	N/A	Referral	PTB	CAT-1	Clinical	Treatment Complete
1161	Civil Hospital	84	M	17	N/A	Screening	PTB	CAT-1	Clinical	Treatment Complete
1162	Civil Hospital	132	M	19.4	N/A	Screening	PTB	CAT-1	Clinical	Treatment Complete
1163	Civil Hospital	108	M	18	N/A	Screening	PTB	CAT-1	Clinical	Lost to follow up
1164	Civil Hospital	120	F	17	N/A	Screening	PTB	CAT-1	Clinical	Lost to follow up
1165	Civil Hospital	72	M	16	N/A	Screening	PTB	CAT-1	Clinical	Treatment Complete
1166	Civil Hospital	60	F	11	N/A	Screening	PTB	CAT-1	Clinical	Treatment Complete
1167	Civil Hospital	48	F	11	N/A	Screening	PTB	CAT-1	Clinical	Treatment Complete
1168	Civil Hospital	60	M	10.6	N/A	Screening	PTB	CAT-1	Clinical	Lost to follow up
1169	Civil Hospital	72	M	14.7	N/A	Referral	EPTB	CAT-1	Clinical	Treatment Complete
1170	Civil Hospital	36	F	11	N/A	Screening	PTB	CAT-1	Clinical	Treatment Complete
1171	Civil Hospital	24	M	9	N/A	Screening	PTB	CAT-1	Clinical	Lost to follow up
1172	Civil Hospital	156	M	26	N/A	Referral	PTB	CAT-1	Clinical	Treatment Complete
1173	Civil Hospital	108	F	21	N/A	Screening	PTB	CAT-1	Clinical	Treatment Complete
1174	Civil Hospital	84	M	16.3	103	Referral	PTB	CAT-1	Clinical	Treatment Complete
1175	Civil Hospital	72	M	15.5	93	Referral	PTB	CAT-1	Clinical	Treatment Complete
1176	Civil Hospital	36	M	10.3	81	Referral	PTB	CAT-1	Clinical	Treatment Complete
1177	Civil Hospital	156	F	32	153	Referral	EPTB	CAT-1	Clinical	Treatment Complete
1178	Civil Hospital	12	M	5.5	N/A	Screening	PTB	CAT-1	Clinical	Treatment Complete
1179	Civil Hospital	36	F	11.9	77	Screening	PTB	CAT-1	Clinical	Treatment Complete
1180	Civil Hospital	96	M	18	112	Screening	PTB	CAT-1	Clinical	Treatment Complete
1181	Civil Hospital	120	F	18	N/A	Referral	PTB	CAT-1	Clinical	Treatment Complete
1182	Civil Hospital	84	M	20	N/A	Screening	PTB	CAT-1	Clinical	Treatment Complete
1183	Civil Hospital	84	F	14.5	N/A	Screening	PTB	CAT-1	Clinical	Lost to follow up
1184	Civil Hospital	120	M	5.8	N/A	Screening	PTB	CAT-1	Clinical	Lost to follow up
1185	Civil Hospital	30	M	10	90	Referral	PTB	CAT-1	Clinical	Treatment Complete
1186	Civil Hospital	108	F	24	N/A	Referral	PTB	CAT-2	Clinical	Lost to follow up
1187	Civil Hospital	60	M	14	N/A	Referral	PTB	CAT-1	Clinical	Treatment Complete
1188	Civil Hospital	144	M	29.4	N/A	Referral	PTB	CAT-1	Clinical	Treatment Complete

1189	Civil Hospital	108	M	18	N/A	Referral	EPTB	CAT-1	Clinical	Treatment Complete
1190	Civil Hospital	84	M	18	N/A	Referral	PTB,EPTB	CAT-1	Clinical	Treatment Complete
1191	Civil Hospital	96	F	15	N/A	Referral	PTB	CAT-1	Clinical	Treatment Complete
1192	Civil Hospital	84	F	21	N/A	Screening	PTB	CAT-1	Clinical	Lost to follow up
1193	Civil Hospital	108	F	25	N/A	Screening	EPTB	CAT-1	Clinical	Treatment Complete
1194	Civil Hospital	96	F	22	N/A	Referral	PTB	CAT-1	Clinical	Treatment Complete
1195	Civil Hospital	48	M	12	N/A	Screening	PTB	CAT-1	B+	Lost to follow up
1196	Civil Hospital	120	M	16	N/A	Referral	PTB,EPTB	CAT-1	B+	Treatment Complete
1197	Civil Hospital	120	M	17	N/A	N/A	PTB	CAT-1	Clinical	Treatment Complete
1198	Civil Hospital	36	F	8.9	N/A	N/A	PTB	CAT-1	Clinical	Treatment Complete
1199	Civil Hospital	72	F	13	N/A	N/A	PTB	CAT-1	Clinical	Treatment Complete
1200	Civil Hospital	18	M	7	N/A	N/A	PTB	CAT-1	Clinical	Lost to follow up
1201	Civil Hospital	72	F	12	N/A	Referral	PTB	CAT-1	Clinical	Lost to follow up
1202	Civil Hospital	60	M	12	97	N/A	PTB	CAT-1	Clinical	Treatment Complete
1203	Civil Hospital	108	F	13	N/A	Referral	PTB	CAT-1	Clinical	Lost to follow up
1204	Civil Hospital	78	M	15.6	109	N/A	PTB	CAT-1	Clinical	Treatment Complete
1205	Civil Hospital	252	M	10.5	N/A	N/A	PTB	CAT-1	Clinical	Treatment Complete
1206	Civil Hospital	120	M	18.9	115	N/A	PTB	CAT-1	Clinical	Treatment Complete
1207	Civil Hospital	30	F	9.4	86	N/A	PTB	CAT-1	Clinical	Treatment Complete
1208	Civil Hospital	96	M	16.5	108	N/A	PTB	CAT-1	Clinical	Treatment Complete
1209	Civil Hospital	12	F	7.9	N/A	N/A	PTB	CAT-1	Clinical	Treatment Complete
1210	Civil Hospital	240	F	8.5	N/A	N/A	PTB	CAT-1	Clinical	Treatment Complete
1211	Civil Hospital	72	M	17.2	111	Referral	PTB	CAT-1	Clinical	Treatment Complete
1212	Civil Hospital	36	M	11.6	87	N/A	PTB	CAT-1	Clinical	Treatment Complete
1213	Civil Hospital	120	F	21.2	119	N/A	PTB	CAT-1	Clinical	Treatment Complete
1214	Civil Hospital	72	F	16	N/A	Screening	PTB	CAT-1	Clinical	Treatment Complete
1215	Civil Hospital	60	M	15.5	N/A	Screening	PTB	CAT-1	Clinical	Treatment Complete
1216	Civil Hospital	84	M	13	105	Screening	PTB,EPTB	CAT-1	Clinical	Treatment Complete
1217	Civil Hospital	84	M	18	109	Screening	PTB	CAT-1	Clinical	Treatment Complete
1218	Civil Hospital	96	F	16	111	Screening	PTB	CAT-1	Clinical	Treatment Complete
1219	Civil Hospital	144	F	26	135	Screening	PTB	CAT-1	Clinical	Treatment Complete
1220	Civil Hospital	84	M	20	119	Screening	PTB	CAT-1	Clinical	Treatment Complete
1221	Civil Hospital	84	M	20	122	Screening	PTB	CAT-1	Clinical	Treatment Complete
1222	Civil Hospital	120	F	26	137	Screening	PTB	CAT-1	Clinical	Treatment Complete
1223	Civil Hospital	84	F	14.4	111	Referral	PTB	CAT-1	Clinical	Treatment Complete
1224	Civil Hospital	60	M	13	98	Referral	PTB	CAT-1	Clinical	Treatment Complete
1225	Civil Hospital	36	M	9.5	85	Referral	PTB	CAT-1	Clinical	Treatment Complete
1226	Civil Hospital	24	M	8.5	78	Screening	PTB	CAT-1	Clinical	Treatment Complete
1227	Civil Hospital	24	F	8	77	Screening	PTB	CAT-1	Clinical	Treatment Complete
1228	Civil Hospital	12	F	7	70	Screening	PTB	CAT-1	Clinical	Treatment Complete
1229	Civil Hospital	72	F	15	112	Referral	PTB,EPTB	CAT-1	B+	Treatment Complete

1230	Civil Hospital	36	F	12.5	92	Screening	PTB	CAT-1	Clinical	Treatment Complete
1231	Civil Hospital	84	M	6.8	65	Screening	PTB	CAT-1	Clinical	Cure
1232	Civil Hospital	48	M	14	96	Screening	PTB	CAT-1	Clinical	Treatment Complete
1233	Civil Hospital	168	F	8.1	70	Screening	PTB	CAT-1	Clinical	Treatment Complete
1234	Civil Hospital	72	M	15	103	Screening	PTB	CAT-1	Clinical	Treatment Complete
1235	Civil Hospital	84	F	13.5	98	Screening	PTB	CAT-1	Clinical	Treatment Complete
1236	Civil Hospital	120	F	21	124	Screening	EPTB	CAT-1	Clinical	Treatment Complete
1237	Civil Hospital	24	M	8.8	N/A	Screening	PTB	CAT-1	Clinical	Treatment Complete
1238	Civil Hospital	48	M	12	94	Screening	PTB	CAT-1	Clinical	Treatment Complete
1239	Civil Hospital	144	F	33.6	147	Screening	PTB	CAT-1	Clinical	Treatment Complete
1240	Civil Hospital	24	M	7.1	75	Referral	PTB	CAT-1	Clinical	Treatment Complete
1241	Civil Hospital	36	M	7.5	80	Referral	PTB	CAT-1	Clinical	Lost to follow up
1242	Civil Hospital	84	F	20	131	Screening	PTB,EPTB	CAT-1	Clinical	Treatment Complete
1243	Civil Hospital	84	F	18	108	Referral	EPTB	CAT-1	Clinical	Transfer out
1244	Civil Hospital	24	F	7.5	N/A	Screening	PTB	CAT-1	Clinical	Treatment Complete
1245	Civil Hospital	156	M	6.9	N/A	Referral	PTB	CAT-1	Clinical	Treatment Complete
1246	Civil Hospital	48	M	14	98	Screening	PTB	CAT-1	Clinical	Treatment Complete
1247	Civil Hospital	84	M	19	123	Screening	PTB	CAT-1	Clinical	Treatment Complete
1248	Civil Hospital	36	M	11	91	Screening	PTB	CAT-1	Clinical	Treatment Complete
1249	Civil Hospital	24	F	9	82	Screening	PTB	CAT-1	Clinical	Treatment Complete
1250	Civil Hospital	36	M	12	91	Screening	PTB	CAT-1	Clinical	Treatment Complete
1251	Civil Hospital	144	F	25	135	Screening	PTB	CAT-1	Clinical	Treatment Complete
1252	Civil Hospital	30	F	9	80	Screening	PTB	CAT-1	Clinical	Treatment Complete
1253	Civil Hospital	48	M	11	N/A	Screening	PTB	CAT-2	Clinical	Treatment Complete
1254	Civil Hospital	72	M	14	N/A	Screening	PTB,EPTB	CAT-1	Clinical	Treatment Complete
1255	Civil Hospital	24	M	9	N/A	Screening	PTB	CAT-1	Clinical	Treatment Complete
1256	Civil Hospital	60	M	14	101	Referral	PTB	CAT-1	Clinical	Treatment Complete
1257	Civil Hospital	60	M	19	110	Screening	PTB	CAT-1	Clinical	Treatment Complete
1258	Civil Hospital	60	M	17	N/A	Screening	PTB	CAT-1	Clinical	Treatment Complete
1259	Civil Hospital	96	M	N/A	7.5	Screening	PTB	CAT-1	Clinical	Treatment Complete
1260	Civil Hospital	18	M	9	N/A	Screening	EPTB	CAT-1	Clinical	Treatment Complete
1261	Civil Hospital	72	M	17	108	Screening	PTB	CAT-1	Clinical	Treatment Complete
1262	Civil Hospital	84	M	16	N/A	Screening	PTB	CAT-1	Clinical	Treatment Complete
1263	Civil Hospital	48	M	11	N/A	Screening	PTB	CAT-1	Clinical	Lost to follow up
1264	Civil Hospital	132	M	25	N/A	Screening	PTB	CAT-1	Clinical	Treatment Complete
1265	Civil Hospital	48	F	12	N/A	Screening	PTB	CAT-1	Clinical	Treatment Complete
1266	Civil Hospital	84	F	18	N/A	Screening	PTB,EPTB	CAT-1	Clinical	Treatment Complete
1267	Civil Hospital	120	F	21	N/A	Screening	PTB	CAT-1	Clinical	Treatment Complete
1268	Civil Hospital	24	F	8.8	N/A	Screening	PTB	CAT-1	Clinical	Treatment Complete
1269	Civil Hospital	144	F	27	N/A	Screening	PTB,EPTB	CAT-1	Clinical	Treatment Complete
1270	Civil Hospital	132	M	8.5	N/A	Screening	PTB	CAT-1	Clinical	Died

1271	Civil Hospital	120	M	19	N/A	Screening	PTB	CAT-1	Clinical	Treatment Complete
1272	Civil Hospital	132	F	26	N/A	Screening	EPTB	CAT-1	Clinical	Treatment Complete
1273	Civil Hospital	144	M	28	N/A	Screening	PTB	CAT-2	Clinical	Treatment Complete
1274	Civil Hospital	132	M	23	N/A	Screening	PTB,EPTB	CAT-1	Clinical	Treatment Complete
1275	Civil Hospital	48	M	13	N/A	Screening	PTB	CAT-1	Clinical	Treatment Complete
1276	Civil Hospital	120	M	20	N/A	Screening	PTB	CAT-1	Clinical	Treatment Complete
1277	Civil Hospital	72	F	12	103	Screening	PTB,EPTB	CAT-1	Clinical	Treatment Complete
1278	Civil Hospital	72	F	15	103	Referral	PTB,EPTB	CAT-1	Clinical	Treatment Complete
1279	Civil Hospital	60	F	11	83	Referral	PTB	CAT-1	Clinical	Treatment Complete
1280	Civil Hospital	108	M	22	130	Referral	PTB	CAT-2	Clinical	Lost to follow up
1281	Civil Hospital	120	M	26	136	Referral	PTB	CAT-1	Clinical	Treatment Complete
1282	Civil Hospital	132	M	24	N/A	Referral	PTB,EPTB	CAT-1	Clinical	Treatment Complete
1283	Civil Hospital	84	M	17	N/A	Screening	PTB	CAT-1	Clinical	Treatment Complete
1284	Civil Hospital	96	F	17	106	Screening	PTB	CAT-1	Clinical	Treatment Complete
1285	Civil Hospital	96	F	19	112	Referral	PTB	CAT-1	Clinical	Treatment Complete
1286	Civil Hospital	132	F	32	140	Screening	PTB	CAT-1	Clinical	Treatment Complete
1287	Civil Hospital	108	M	17.3	116	Screening	PTB,EPTB	CAT-1	Clinical	Treatment Complete
1288	Civil Hospital	144	M	28.5	138	Screening	PTB,EPTB	CAT-1	Clinical	Treatment Complete
1289	Civil Hospital	84	M	21	115	Screening	PTB,EPTB	CAT-1	Clinical	Treatment Complete
1290	Civil Hospital	48	M	8.9	80	Screening	PTB	CAT-1	Clinical	Treatment Complete
1291	Civil Hospital	60	F	13	93	Screening	PTB,EPTB	CAT-1	Clinical	Treatment Complete
1292	Civil Hospital	48	F	11.6	84	Screening	EPTB	CAT-1	Clinical	Treatment Complete
1293	Civil Hospital	60	M	17.5	N/A	Screening	PTB	CAT-1	Clinical	Treatment Complete
1294	Civil Hospital	84	M	20.2	N/A	Screening	PTB	CAT-1	Clinical	Treatment Complete
1295	Civil Hospital	132	F	27	136	Screening	PTB	CAT-1	Clinical	Treatment Complete
1296	Civil Hospital	108	M	24	122	Referral	PTB	CAT-1	Clinical	Treatment Complete
1297	Civil Hospital	84	F	19.5	113	Referral	PTB	CAT-1	Clinical	Treatment Complete
1298	Civil Hospital	108	F	17.5	115	Screening	PTB	CAT-1	Clinical	Lost to follow up
1299	Civil Hospital	24	F	7.1	N/A	Screening	PTB	CAT-1	Clinical	Treatment Complete
1300	Civil Hospital	120	F	20.5	112	Referral	PTB	CAT-1	Clinical	Treatment Complete
1301	Civil Hospital	96	M	20.3	119	Screening	EPTB	CAT-1	Clinical	Treatment Complete
1302	Civil Hospital	96	M	16	108	Referral	PTB,EPTB	CAT-1	Clinical	Lost to follow up
1303	Civil Hospital	84	M	18.2	115	Referral	EPTB	CAT-1	Clinical	Treatment Complete
1304	Civil Hospital	24	M	10	80	Referral	EPTB	CAT-1	Clinical	Treatment Complete
1305	Civil Hospital	84	M	15	100	Screening	EPTB	CAT-1	Clinical	Treatment Complete
1306	Civil Hospital	108	M	23.4	122	Screening	PTB	CAT-1	Clinical	Treatment Complete
1307	Civil Hospital	132	M	22	125	Screening	PTB	CAT-1	Clinical	Treatment Complete
1308	Civil Hospital	24	M	9.8	86	Screening	PTB	CAT-1	B+	Treatment Complete
1309	Civil Hospital	60	F	14.3	101	Screening	PTB,EPTB	CAT-1	Clinical	Treatment Complete
1310	Civil Hospital	84	M	17.6	107	Screening	PTB	CAT-1	Clinical	Treatment Complete
1311	Civil Hospital	96	M	18.7	118	Screening	PTB	CAT-1	Clinical	Treatment Complete

1312	Civil Hospital	60	M	15	98	Screening	PTB	CAT-1	Clinical	Lost to follow up
1313	Civil Hospital	36	M	11.6	85	Screening	PTB	CAT-1	Clinical	Lost to follow up
1314	Civil Hospital	48	F	12.4	N/A	Walk-In	PTB	CAT-1	Clinical	Treatment Complete
1315	Civil Hospital	132	F	24	N/A	Screening	PTB	CAT-1	Clinical	Treatment Complete
1316	Civil Hospital	84	M	16	110	Screening	PTB	CAT-2	Clinical	Treatment Complete
1317	Civil Hospital	84	M	19	110	Screening	PTB	CAT-1	Clinical	Treatment Complete
1318	Civil Hospital	36	M	13.5	N/A	Screening	PTB	CAT-1	Clinical	Treatment Complete
1319	Civil Hospital	36	F	11	87	Screening	PTB	CAT-1	Clinical	Treatment Complete
1320	Civil Hospital	84	F	20.8	120.5	Screening	PTB	CAT-1	Clinical	Treatment Complete
1321	Civil Hospital	60	F	17	N/A	Screening	PTB	CAT-1	Clinical	Treatment Complete
1322	Civil Hospital	60	F	12.8	101	Screening	PTB	CAT-1	Clinical	Treatment Complete
1323	Civil Hospital	120	M	20.5	125	Screening	PTB	CAT-1	Clinical	Treatment Complete
1324	Civil Hospital	120	M	27.8	136	Screening	PTB	CAT-1	Clinical	Treatment Complete
1325	Civil Hospital	96	F	19.8	114	Screening	EPTB	CAT-1	Clinical	Still on treatment
1326	Civil Hospital	84	M	16.9	115	Screening	PTB,EPTB	CAT-2	Clinical	Transfer out
1327	Civil Hospital	132	M	30	N/A	Screening	PTB	CAT-1	Clinical	Treatment Complete
1328	Civil Hospital	60	M	15	104	Screening	EPTB	CAT-1	Clinical	Still on treatment
1329	Civil Hospital	120	M	32.2	N/A	Screening	PTB	CAT-1	Clinical	Treatment Complete
1330	Civil Hospital	96	M	23	N/A	Screening	PTB	CAT-1	Clinical	Treatment Complete
1331	Civil Hospital	72	F	14	N/A	Screening	PTB	CAT-1	Clinical	Treatment Complete
1332	Civil Hospital	60	M	13.6	103	Screening	PTB	CAT-1	Clinical	Lost to follow up
1333	NICH	48	F	5.3	N/A	Screening	PTB	CAT-1	Clinical	Treatment Complete
1334	NICH	48	F	16	N/A	Screening	PTB	CAT-1	Clinical	Treatment Complete
1335	NICH	108	M	25	N/A	Screening	PTB	CAT-1	Clinical	Treatment Complete
1336	NICH	60	M	17	N/A	Screening	PTB	CAT-1	Clinical	Treatment Complete
1337	NICH	60	F	13.5	N/A	Screening	PTB	CAT-1	Clinical	Transfer out
1338	NICH	108	F	26	N/A	Screening	PTB,EPTB	CAT-1	Clinical	Treatment Complete
1339	NICH	84	M	24	123	Screening	PTB	CAT-1	Clinical	Treatment Complete
1340	NICH	120	F	25	129	Screening	PTB	CAT-1	Clinical	Transfer out
1341	NICH	48	F	11.1	88	Screening	PTB	CAT-1	Clinical	Treatment Complete
1342	NICH	96	M	24	127	Screening	PTB	CAT-1	Clinical	Treatment Complete
1343	NICH	54	M	12	91	Screening	PTB	CAT-2	Clinical	Treatment Complete
1344	NICH	72	M	13	93	Screening	PTB	CAT-1	Clinical	Treatment Complete
1345	NICH	144	M	26	128	Screening	EPTB	CAT-1	Clinical	Treatment Complete
1346	NICH	120	M	23	134	Screening	PTB	CAT-1	Clinical	Treatment Complete
1347	NICH	168	M	25	128	Screening	PTB	CAT-1	Clinical	Transfer out
1348	NICH	84	M	21	120	Screening	PTB	CAT-1	Clinical	Treatment Complete
1349	NICH	108	M	25	129	Screening	PTB	CAT-1	Clinical	Treatment Complete
1350	NICH	114	M	18	N/A	Screening	PTB	CAT-1	B+	Lost to follow up
1351	NICH	24	M	11	N/A	Screening	EPTB	CAT-1	B+	Lost to follow up
1352	NICH	84	F	20	137	Screening	EPTB	CAT-1	Clinical	Treatment Complete

1353	NICH	60	F	10	100	Referral	PTB,EPTB	CAT-1	B+	Lost to follow up
1354	NICH	54	M	14	N/A	Referral	EPTB	CAT-1	B+	Died
1355	NICH	84	M	20	120	Screening	PTB	CAT-1	B+	Treatment Complete
1356	NICH	132	F	21	N/A	Referral	EPTB	CAT-1	Clinical	Died
1357	NICH	72	M	15	N/A	Referral	PTB,EPTB	CAT-1	Clinical	Died
1358	NICH	24	F	10	80	Referral	EPTB	CAT-1	Clinical	Transfer out
1359	NICH	48	M	11	87	Screening	EPTB	CAT-1	Clinical	Treatment Complete
1360	NICH	120	F	25	N/A	Screening	EPTB	CAT-1	B+	Transfer out
1361	NICH	120	F	19	117	Screening	PTB	CAT-1	B+	Treatment Complete
1362	NICH	132	M	19	N/A	Referral	PTB	CAT-1	Clinical	Treatment Complete
1363	NICH	84	M	22.5	N/A	Referral	PTB,EPTB	CAT-1	B+	Died
1364	NICH	96	F	19	120	Screening	PTB	CAT-1	Clinical	Treatment Complete
1365	NICH	15.6	F	6	N/A	Referral	PTB	CAT-1	B+	Lost to follow up
1366	NICH	72	F	17	N/A	Screening	PTB	CAT-1	Clinical	Treatment Complete
1367	NICH	132	F	26	135	Screening	PTB	CAT-1	Clinical	Treatment Complete
1368	NICH	96	M	21	128	Screening	EPTB	CAT-1	Clinical	Treatment Complete
1369	NICH	144	M	28	132	Screening	PTB	CAT-1	B+	Treatment Complete
1370	NICH	120	M	19	115	Screening	PTB	CAT-1	Clinical	Treatment Complete
1371	NICH	120	M	20	120	Screening	EPTB	CAT-1	Clinical	Treatment Complete
1372	NICH	84	F	16	115	Referral	PTB	CAT-1	B+	Treatment Complete
1373	NICH	120	M	29	150	Referral	EPTB	CAT-1	Clinical	Treatment Complete
1374	NICH	84	F	4.5	76	Screening	PTB	CAT-1	Clinical	Treatment Complete
1375	NICH	144	F	21	133	Screening	EPTB	CAT-1	Clinical	Treatment Complete
1376	NICH	120	M	20	125	Screening	PTB	CAT-1	Clinical	Treatment Complete
1377	NICH	132	M	25	118	Referral	EPTB	CAT-1	Clinical	Treatment Complete
1378	NICH	156	M	8	79	Screening	PTB	CAT-1	Clinical	Treatment Complete
1379	NICH	30	F	7	75	Screening	PTB	CAT-1	Clinical	Lost to follow up
1380	NICH	48	M	12	90	Screening	PTB	CAT-1	Clinical	Treatment Complete
1381	NICH	84	M	19	119	Screening	PTB	CAT-1	Clinical	Treatment Complete
1382	NICH	84	F	14	112	Screening	PTB	CAT-1	Clinical	Treatment Complete
1383	NICH	144	M	27	134	Screening	EPTB	CAT-1	Clinical	Lost to follow up
1384	NICH	84	M	21	123	Screening	EPTB	CAT-1	Clinical	Lost to follow up
1385	NICH	96	M	16	107	Screening	PTB	CAT-1	Clinical	Treatment Complete
1386	NICH	18	M	8	N/A	Screening	PTB	CAT-1	Clinical	Lost to follow up
1387	NICH	168	M	21	128	Referral	PTB	CAT-1	B+	Lost to follow up
1388	NICH	24	M	9	76	Screening	PTB,EPTB	CAT-1	Clinical	Lost to follow up
1389	NICH	144	F	27	145	Referral	PTB	CAT-1	Clinical	Lost to follow up
1390	NICH	144	M	28	133	Screening	PTB	CAT-1	Clinical	Treatment Complete
1391	NICH	72	M	72		Screening	PTB	CAT-1	Clinical	Treatment Complete
1392	NICH	24	F	9	80	Screening	PTB	CAT-1	Clinical	Treatment Complete
1393	NICH	60	F	14	97	Screening	PTB	CAT-1	Clinical	Treatment Complete

1394	NICH	36	F	11	84	Screening	PTB	CAT-1	Clinical	Treatment Complete
1395	NICH	84	F	28	143	Screening	PTB	CAT-1	Clinical	Treatment Complete
1396	NICH	120	M	28	133	Screening	PTB	CAT-1	Clinical	Treatment Complete
1397	NICH	132	F	22	141	Referral	PTB	CAT-1	Clinical	Treatment Complete
1398	NICH	120	F	26	143	Screening	EPTB	CAT-1	Clinical	Treatment Complete
1399	NICH	120	M	18	131	Screening	EPTB	CAT-1	Clinical	Treatment Complete
1400	NICH	168	F	8	74	Screening	PTB	CAT-1	Clinical	Treatment Complete
1401	NICH	120	F	20	127	Screening	PTB	CAT-1	Clinical	Treatment Complete
1402	NICH	84	M	16	115	Screening	PTB	CAT-1	Clinical	Lost to follow up
1403	NICH	36	M	13	91	Screening	PTB	CAT-1	Clinical	Treatment Complete
1404	NICH	18	M	9	74	Screening	PTB	CAT-1	Clinical	Treatment Complete
1405	NICH	120	M	24	133	Screening	PTB	CAT-1	Clinical	Lost to follow up
1406	NICH	24	F	8	75	Screening	PTB	CAT-1	Clinical	Treatment Complete
1407	NICH	120	M	24	127	Screening	PTB	CAT-1	Clinical	Treatment Complete
1408	NICH	84	F	14	109	Screening	EPTB	CAT-1	Clinical	Treatment Complete
1409	NICH	120	F	20	136	Screening	EPTB	CAT-1	Clinical	Treatment Complete
1410	NICH	24	F	8	86	Screening	EPTB	CAT-1	Clinical	Treatment Complete
1411	NICH	120	F	26	141	Screening	EPTB	CAT-1	Clinical	Treatment Complete
1412	NICH	108	F	18	117	Screening	PTB	CAT-1	Clinical	Treatment Complete
1413	NICH	132	F	20	133	Screening	EPTB	CAT-1	Clinical	Treatment Complete
1414	NICH	144	M	25	132	Screening	PTB,EPTB	CAT-1	Clinical	Lost to follow up
1415	NICH	24	M	7	70	Screening	PTB	CAT-1	Clinical	Treatment Complete
1416	NICH	96	F	15	107	Screening	PTB	CAT-1	Clinical	Lost to follow up
1417	NICH	144	M	42	136	Screening	PTB	CAT-1	Clinical	Treatment Complete
1418	NICH	60	M	15	97	Screening	PTB	CAT-1	Clinical	Treatment Complete
1419	NICH	84	M	16	109	Screening	PTB	CAT-1	Clinical	Treatment Complete
1420	NICH	120	F	30	138	Screening	EPTB	CAT-1	Clinical	Treatment Complete
1421	NICH	132	F	21	134	Screening	PTB	CAT-1	Clinical	Treatment Complete
1422	NICH	60	F	14	107	Screening	PTB	CAT-1	Clinical	Treatment Complete
1423	NICH	60	F	14	104	Screening	PTB	CAT-1	Clinical	Treatment Complete
1424	NICH	24	M	8	84	Screening	PTB	CAT-1	Clinical	Treatment Complete
1425	NICH	24	M	4	79	Screening	PTB	CAT-1	Clinical	Lost to follow up
1426	NICH	48	M	15	103	Screening	EPTB	CAT-1	Clinical	Lost to follow up
1427	NICH	96	F	13	139	Screening	PTB	CAT-1	Clinical	Treatment Complete
1428	NICH	36	F	11	76	Screening	PTB	CAT-1	Clinical	Lost to follow up
1429	NICH	120	F	26	139	Screening	PTB	CAT-1	Clinical	Treatment Complete
1430	NICH	12	F	6	61	Screening	PTB	CAT-1	Clinical	Treatment Complete
1431	NICH	36	F	8	76	Screening	PTB	CAT-1	Clinical	Lost to follow up
1432	NICH	19.2	M	4	61	Referral	PTB	CAT-1	B+	Lost to follow up
1433	NICH	156	F	35	139	Screening	PTB	CAT-1	Clinical	Lost to follow up
1434	NICH	30	F	8.5	87	Screening	PTB	CAT-1	Clinical	Treatment Complete

1435	NICH	60	M	14	106	Screening	PTB,EPTB	CAT-1	Clinical	Treatment Complete
1436	NICH	30	F	10	76	Screening	PTB	CAT-1	Clinical	Treatment Complete
1437	NICH	19.2	F	10	84	Screening	PTB	CAT-1	Clinical	Lost to follow up
1438	NICH	60	F	12	103	Screening	EPTB	CAT-1	Clinical	Treatment Complete
1439	NICH	84	M	24	120	Screening	EPTB	CAT-1	Clinical	Treatment Complete
1440	NICH	120	F	20	136	Screening	PTB	CAT-1	Clinical	Treatment Complete
1441	NICH	48	F	13	96	Screening	PTB	CAT-1	Clinical	Transfer out
1442	NICH	120	F	15	124	Screening	PTB	CAT-1	Clinical	Treatment Complete
1443	NICH	36	M	14	91	Screening	EPTB	CAT-1	Clinical	Treatment Complete
1444	NICH	108	M	24	126	Screening	PTB	CAT-1	Clinical	Treatment Complete
1445	NICH	108	M	18	122	Screening	PTB	CAT-1	Clinical	Treatment Complete
1446	NICH	120	M	27	139	Screening	PTB	CAT-1	B+	Treatment Complete
1447	NICH	144	F	37	140	Screening	EPTB	CAT-1	Clinical	Treatment Complete
1448	NICH	132	M	36	138	Screening	PTB	CAT-1	Clinical	Treatment Complete
1449	NICH	24	F	13	82	Screening	PTB	CAT-1	Clinical	Treatment Complete
1450	NICH	84	F	18	106	Screening	PTB	CAT-1	B+	Treatment Complete
1451	NICH	43.2	F	N/A	N/A	Screening	PTB	CAT-1	Clinical	Transfer out
1452	NICH	60	M	17	109	Screening	EPTB	CAT-1	Clinical	Treatment Complete
1453	NICH	84	F	24	116	Screening	EPTB	CAT-1	Clinical	Treatment Complete
1454	NICH	144	F	21	136	Screening	EPTB	CAT-1	Clinical	Treatment Complete
1455	NICH	24	M	7	69	Screening	PTB	CAT-1	Clinical	Treatment Complete
1456	NICH	60	F	15	104	Screening	PTB	CAT-1	Clinical	Treatment Complete
1457	NICH	60	F	12	102	Screening	PTB	CAT-1	Clinical	Treatment Complete
1458	NICH	96	F	20	121	Screening	PTB	CAT-1	Clinical	Treatment Complete
1459	NICH	192	M	10	75	Referral	PTB	CAT-1	Clinical	Treatment Complete
1460	NICH	36	M	13.7	86	Referral	PTB	CAT-1	Clinical	Treatment Complete
1461	NICH	144	F	23	141	Screening	PTB	CAT-1	Clinical	Treatment Complete
1462	NICH	132	F	26	137	Screening	EPTB	CAT-1	Clinical	Treatment Complete
1463	NICH	96	F	20	115	Screening	EPTB	CAT-1	Clinical	Treatment Complete
1464	NICH	36	F	10.5	85	Screening	PTB	CAT-1	Clinical	Treatment Complete
1465	NICH	120	M	22	116	Screening	PTB	CAT-1	Clinical	Treatment Complete
1466	NICH	60	M	15.9	112	Referral	EPTB	CAT-1	Clinical	Still on treatment
1467	NICH	84	M	16	93	Screening	PTB	CAT-1	Clinical	Treatment Complete
1468	NICH	120	F	20	115	Referral	PTB	CAT-2	B+	Treatment Complete
1469	NICH	132	M	27	144	Screening	PTB	CAT-1	Clinical	Treatment Complete
1470	NICH	96	M	17.7	121	Screening	PTB	CAT-1	Clinical	Lost to follow up
1471	NICH	108	F	29	134	Screening	PTB	CAT-1	Clinical	Treatment Complete
1472	NICH	48	M	6	N/A	Screening	PTB	CAT-1	Clinical	Treatment Complete
1473	NICH	132	F	27	139	Screening	PTB	CAT-1	Clinical	Treatment Complete
1474	NICH	60	F	13.5	N/A	Screening	EPTB	CAT-1	Clinical	Still on treatment
1475	NICH	240	F	6.4	71	Screening	PTB	CAT-1	Clinical	Lost to follow up

1476	NICH	60	M	14.8	100	Referral	EPTB	CAT-2	Clinical	Still on treatment
1477	NICH	96	M	16	108	Screening	PTB	CAT-2	Clinical	Treatment Complete
1478	NICH	30	M	13	N/A	Screening	PTB	CAT-1	Clinical	Lost to follow up
1479	NICH	42	M	18	N/A	Screening	PTB	CAT-1	Clinical	Lost to follow up
1480	NICH	120	F	21	130	Screening	PTB,EPTB	CAT-1	Clinical	Treatment Complete
1481	NICH	120	F	23	123	Screening	PTB,EPTB	CAT-1	Clinical	Treatment Complete
1482	NICH	84	M	16	107	Screening	PTB	CAT-1	Clinical	Lost to follow up
1483	NICH	84	M	18	112	Screening	PTB	CAT-1	Clinical	Treatment Complete
1484	NICH	132	F	20.9	123	Screening	PTB	CAT-1	Clinical	Treatment Complete
1485	NICH	96	F	16	110	Screening	PTB	CAT-1	Clinical	Treatment Complete
1486	NICH	84	F	20	117	Screening	PTB	CAT-1	Clinical	Treatment Complete
1487	NICH	48	F	14	99	Screening	PTB	CAT-1	Clinical	Treatment Complete
1488	NICH	60	F	10.5	85	Screening	PTB	CAT-2	Clinical	Transfer out
1489	NICH	84	M	18	105	Screening	EPTB	CAT-1	Clinical	Lost to follow up
1490	NICH	84	M	16.5	105	Screening	EPTB	CAT-1	Clinical	Still on treatment
1491	NICH	96	F	20	120	Screening	PTB	CAT-2	Clinical	Lost to follow up
1492	NICH	48	F	13.9	110	Screening	PTB	CAT-1	Clinical	Treatment Complete
1493	NICH	72	F	16.5	110	Screening	PTB	CAT-1	Clinical	Treatment Complete
1494	NICH	36	F	7	71	Screening	PTB	CAT-1	Clinical	Treatment Complete
1495	NICH	144	F	32.5	148	Screening	EPTB	CAT-1	Clinical	Treatment Complete
1496	NICH	84	M	10.5	106	Screening	PTB	CAT-1	Clinical	Treatment Complete
1497	NICH	48	M	13	98	Screening	PTB	CAT-1	Clinical	Treatment Complete
1498	NICH	24	M	11.6	N/A	Screening	PTB	CAT-1	Clinical	Treatment Complete
1499	NICH	36	F	12.4	85	Screening	PTB	CAT-1	Clinical	Treatment Complete
1500	NICH	144	F	33.6	145	Screening	PTB	CAT-1	Clinical	Treatment Complete
1501	NICH	60	F	13.5	93	Screening	PTB	CAT-1	Clinical	Treatment Complete
1502	NICH	96	M	23.4	122	Screening	PTB	CAT-1	Clinical	Treatment Complete
1503	NICH	168	M	9	71	Screening	PTB	CAT-1	Clinical	Treatment Complete
1504	NICH	120	M	42	162	Screening	PTB	CAT-1	Clinical	Treatment Complete
1505	NICH	84	M	17	106	Screening	PTB	CAT-1	Clinical	Treatment Complete
1506	NICH	84	F	13	106	Screening	PTB	CAT-1	Clinical	Treatment Complete
1507	NICH	60	F	12	91	Screening	PTB	CAT-1	Clinical	Treatment Complete
1508	NICH	120	F	19	125	Screening	PTB	CAT-2	Clinical	Treatment Complete
1509	NICH	48	M	13	92	Screening	EPTB	CAT-1	Clinical	Treatment Complete
1510	NICH	108	F	21	117	Screening	PTB	CAT-1	Clinical	Treatment Complete
1511	NICH	144	F	29.5	134	Screening	PTB	CAT-1	Clinical	Treatment Complete
1512	NICH	24	M	11	90	Screening	PTB	CAT-1	Clinical	Treatment Complete
1513	NICH	120	M	28	135	Screening	PTB	CAT-2	Clinical	Treatment Complete
1514	NICH	108	M	23	126	Referral	EPTB	CAT-1	Clinical	Treatment Complete
1515	NICH	132	F	27	125	Screening	EPTB	CAT-1	Clinical	Still on treatment
1516	NICH	12	F	6	N/A	Screening	PTB	CAT-1	Clinical	Lost to follow up

1517	NICH	36	M	14	89	Screening	PTB	CAT-1	Clinical	Treatment Complete
1518	NICH	96	F	6.5	66	Screening	PTB	CAT-1	Clinical	Treatment Complete
1519	NICH	132	M	52	149	Screening	PTB	CAT-1	Clinical	failure
1520	NICH	84	F	13	96.5	Screening	PTB	CAT-1	Clinical	Treatment Complete
1521	NICH	36	M	13	88	Screening	PTB	CAT-2	Clinical	Treatment Complete
1522	NICH	120	M	25	N/A	Screening	EPTB	CAT-1	Clinical	Still on treatment
1523	NICH	48	F	13	98	Screening	PTB	CAT-1	Clinical	Treatment Complete
1524	NICH	108	M	25	110	Screening	PTB	CAT-2	Clinical	Treatment Complete
1525	NICH	12	M	9.5	72	Referral	PTB	CAT-1	Clinical	Treatment Complete
1526	NICH	48	M	14	89	Screening	PTB	CAT-1	Clinical	Lost to follow up
1527	NICH	60	M	15	107	Screening	EPTB	CAT-1	Clinical	Treatment Complete
1528	NICH	144	F	32	138	Screening	EPTB	CAT-1	Clinical	Treatment Complete
1529	NICH	96	F	26.5	118	Referral	PTB	CAT-2	Clinical	Treatment Complete
1530	NICH	84	M	18.5	111	Referral	EPTB	CAT-1	Clinical	Treatment Complete
1531	NICH	144	F	33	142	Referral	EPTB	CAT-1	Clinical	Treatment Complete
1532	NICH	60	F	13.2	105	Screening	PTB	CAT-1	Clinical	Treatment Complete
1533	NICH	96	F	18	109	Referral	PTB	CAT-1	Clinical	Treatment Complete
1534	NICH	36	M	12	88	Screening	PTB	CAT-1	Clinical	Treatment Complete
1535	NICH	72	F	14	102	Screening	PTB	CAT-1	Clinical	Treatment Complete
1536	NICH	120	F	19.4	121	Screening	EPTB	CAT-1	Clinical	Treatment Complete
1537	NICH	48	M	11	90	Screening	PTB	CAT-1	Clinical	Treatment Complete
1538	NICH	72	M	16	102	Screening	PTB	CAT-1	Clinical	Lost to follow up
1539	NICH	156	M	29	136	Screening	PTB	CAT-2	Clinical	Treatment Complete
1540	NICH	24	F	8.7	74	Screening	PTB	CAT-1	Clinical	Treatment Complete
1541	NICH	84	F	16.3	102	Screening	PTB	CAT-1	Clinical	Transfer out
1542	NICH	144	F	36.9	138	Screening	EPTB	CAT-1	Clinical	Treatment Complete
1543	NICH	36	F	10.9	85	Screening	PTB	CAT-1	Clinical	Treatment Complete
1544	NICH	72	M	17.2	109	Screening	EPTB	CAT-1	Clinical	Lost to follow up
1545	NICH	120	M	23	120	Screening	PTB	CAT-1	Clinical	Treatment Complete
1546	NICH	96	M	17.6	111	Screening	PTB	CAT-1	Clinical	Treatment Complete
1547	NICH	12	M	7.6	71	Screening	PTB	CAT-1	Clinical	Treatment Complete
1548	NICH	36	F	7	97	Screening	PTB	CAT-1	Clinical	Transfer out
1549	NICH	60	F	14	90	Referral	PTB	CAT-1	Clinical	Treatment Complete
1550	NICH	N/A	M	4.5	65	Screening	PTB	CAT-1	Clinical	Treatment Complete
1551	NICH	168	M	40	161	Screening	PTB	CAT-1	Clinical	Treatment Complete
1552	NICH	36	M	16.5	94	Referral	PTB	CAT-1	Clinical	Treatment Complete
1553	NICH	144	F	26	132	Screening	PTB	CAT-1	Clinical	Treatment Complete
1554	NICH	12	M	7.7	71	Screening	PTB	CAT-1	Clinical	Treatment Complete
1555	NICH	31.2	F	12.5	85	Screening	EPTB	CAT-1	Clinical	Treatment Complete
1556	NICH	24	F	10	85	Screening	PTB	CAT-1	Clinical	Treatment Complete
1557	NICH	24	F	9	80	Screening	PTB	CAT-1	Clinical	Treatment Complete

1558	NICH	156	F	23	121	Screening	PTB	CAT-1	Clinical	Treatment Complete
1559	NICH	48	F	13	91	Screening	PTB	CAT-1	Clinical	Transfer out
1560	NICH	48	F	13	85	Screening	PTB	CAT-1	Clinical	Treatment Complete
1561	Murshid Hospital	72	F	15.6	111	Screening	EPTB	CAT-1	Clinical	Treatment Complete
1562	Murshid Hospital	120	M	19	120	Referral	EPTB	CAT-1	Clinical	Lost to follow up
1563	Murshid Hospital	132	F	38	143	Referral	EPTB	CAT-1	Clinical	Treatment Complete
1564	Murshid Hospital	108	M	24	126	Referral	PTB	CAT-1	Clinical	Cure
1565	Murshid Hospital	84	F	18	105	Screening	PTB	CAT-1	Clinical	Treatment Complete
1566	Murshid Hospital	72	M	14.5	102	Screening	PTB	CAT-1	Clinical	Treatment Complete
1567	Murshid Hospital	120	M	22.5	126	Screening	PTB	CAT-1	Clinical	Treatment Complete
1568	Murshid Hospital	156	F	25	145	Screening	EPTB	CAT-1	Clinical	Treatment Complete
1569	Murshid Hospital	24	M	11.4	86	Transfer In	PTB	CAT-1	Clinical	Transfer out
1570	Murshid Hospital	168	F	50	165	Screening	PTB	CAT-1	Clinical	Treatment Complete
1571	Murshid Hospital	144	F	25	125	Screening	PTB	CAT-1	Clinical	Treatment Complete
1572	Murshid Hospital	156	M	26.4	149	Screening	PTB	CAT-1	Clinical	Treatment Complete
1573	Murshid Hospital	156	F	32.4	148	Screening	PTB,EPTB	CAT-1	Clinical	Transfer out
1574	Murshid Hospital	156	F	21.3	148	Screening	PTB	CAT-1	Clinical	Transfer out
1575	Murshid Hospital	72	M	14.9	106	Screening	PTB	CAT-1	Clinical	Treatment Complete
1576	Murshid Hospital	12	M	5	61	Screening	PTB	CAT-1	Clinical	Died
1577	Murshid Hospital	96	F	20	117	Screening	PTB	CAT-1	Clinical	Treatment Complete
1578	Murshid Hospital	84	M	16.3	104	Screening	PTB	CAT-1	Clinical	Lost to follow up
1579	Murshid Hospital	43.2	M	9	83	Screening	PTB	CAT-1	Clinical	Lost to follow up
1580	Murshid Hospital	60	F	14	104	Screening	PTB	CAT-1	Clinical	Treatment Complete
1581	Murshid Hospital	156	F	32	148	Screening	PTB	CAT-1	Clinical	Treatment Complete
1582	Murshid Hospital	55.2	M	17.6	105	Screening	PTB	CAT-1	Clinical	Lost to follow up
1583	Murshid Hospital	72	F	17	111	Screening	PTB	CAT-1	Clinical	Treatment Complete
1584	Murshid Hospital	12	M	6	70	Screening	PTB	CAT-1	Clinical	Lost to follow up
1585	Murshid Hospital	168	M	34	154	Screening	PTB	CAT-1	Clinical	Treatment Complete
1586	Murshid Hospital	156	M	32.6	151	Screening	PTB	CAT-1	Clinical	Lost to follow up
1587	Murshid Hospital	36	F	11	8	Screening	PTB	CAT-1	Clinical	Treatment Complete
1588	Murshid Hospital	108	F	23	129	Screening	PTB	CAT-1	Clinical	Treatment Complete
1589	Murshid Hospital	36	M	10	88	Screening	PTB	CAT-1	Clinical	Lost to follow up
1590	Murshid Hospital	84	M	18.5	112	Screening	PTB	CAT-1	Clinical	Treatment Complete
1591	Murshid Hospital	132	F	28.7	138	Screening	PTB	CAT-1	Clinical	Lost to follow up
1592	Murshid Hospital	48	F	12	115	Transfer In	EPTB	CAT-1	Clinical	Treatment Complete
1593	Murshid Hospital	31.2	F	13.5	86	Screening	PTB	CAT-1	Clinical	Lost to follow up
1594	Murshid Hospital	132	M	22	127	Screening	PTB	CAT-1	Clinical	Treatment Complete
1595	Murshid Hospital	72	F	17	110	Screening	PTB	CAT-1	Clinical	Lost to follow up
1596	Murshid Hospital	84	F	20	111	Referral	PTB	CAT-1	Clinical	Lost to follow up
1597	SGH Qatar	120	F	20	126	Screening	EPTB	CAT-1	Clinical	Treatment Complete
1598	SGH Qatar	18	F	4.8	72	Screening	PTB	CAT-1	Clinical	Treatment Complete

1599	SGH Qatar	120	F	27	130	Screening	PTB,EPTB	CAT-1	B+	Treatment Complete
1600	SGH Qatar	84	M	17	114	Screening	PTB	CAT-1	Clinical	Treatment Complete
1601	SGH Qatar	12	F	7.4	74	Screening	PTB	CAT-1	Clinical	Treatment Complete
1602	SGH Qatar	72	M	16	105	Screening	PTB	CAT-1	Clinical	Treatment Complete
1603	SGH Qatar	96	M	25	124	Screening	PTB	CAT-1	Clinical	Treatment Complete
1604	SGH Qatar	24	M	14	84	Screening	PTB	CAT-1	Clinical	Treatment Complete
1605	SGH Qatar	84	M	19	121	Screening	PTB	CAT-1	Clinical	Treatment Complete
1606	SGH Qatar	48	M	13	98	Screening	PTB	CAT-1	Clinical	Treatment Complete
1607	SGH Qatar	24	F	9	80	Screening	PTB	CAT-1	Clinical	Treatment Complete
1608	SGH Qatar	48	F	12	91	Screening	PTB	CAT-1	Clinical	Treatment Complete
1609	SGH Qatar	96	M	17.5	110	Screening	PTB	CAT-1	Clinical	Treatment Complete
1610	SGH Qatar	120	M	19	121	Screening	PTB	CAT-1	Clinical	Treatment Complete
1611	SGH Qatar	108	F	25	128	Screening	EPTB	CAT-1	Clinical	Treatment Complete
1612	SGH Qatar	60	M	12	97	Screening	PTB	CAT-1	Clinical	Treatment Complete
1613	SGH Qatar	60	F	14	104	Screening	PTB	CAT-1	Clinical	Treatment Complete
1614	SGH Qatar	24	F	7.3	72	Screening	PTB	CAT-1	Clinical	Died
1615	SGH Qatar	96	M	15	115	Screening	PTB	CAT-1	Clinical	Treatment Complete
1616	SGH Qatar	96	M	17	126	Screening	PTB	CAT-1	Clinical	Treatment Complete
1617	SGH Qatar	72	M	15.9	110	Screening	PTB	CAT-1	Clinical	Treatment Complete
1618	SGH Qatar	72	F	15.9	114	Screening	PTB	CAT-1	Clinical	Treatment Complete
1619	SGH Qatar	36	M	10	79	Screening	PTB	CAT-1	Clinical	Treatment Complete
1620	SGH Qatar	48	M	16.9	99	Screening	PTB	CAT-1	Clinical	Treatment Complete
1621	SGH Qatar	36	M	12.6	90	Screening	PTB	CAT-1	Clinical	Treatment Complete
1622	SGH Qatar	84	F	16.9	110	Screening	PTB	CAT-1	Clinical	Treatment Complete
1623	SGH Qatar	108	F	24	133	Screening	PTB	CAT-1	Clinical	Treatment Complete
1624	SGH Qatar	72	M	19	113	Screening	EPTB	CAT-2	Clinical	Treatment Complete
1625	SGH Qatar	72	F	14	96	Screening	PTB	CAT-1	Clinical	Treatment Complete
1626	SGH Qatar	120	F	20.2	120	Screening	PTB	CAT-1	Clinical	Died
1627	SGH Qatar	24	M	9.4	75	Screening	PTB	CAT-1	Clinical	Treatment Complete
1628	SGH Qatar	48	M	12	87	Screening	PTB	CAT-1	Clinical	Treatment Complete
1629	SGH Qatar	13	M	6.6	69	Screening	PTB	CAT-1	Clinical	Treatment Complete
1630	SGH Qatar	84	M	17.9	120	Screening	PTB	CAT-1	Clinical	Treatment Complete
1631	SGH Qatar	96	F	20	121	Screening	EPTB	CAT-1	Clinical	Treatment Complete
1632	SGH Qatar	72	F	14	113	Screening	EPTB	CAT-1	Clinical	Treatment Complete
1633	SGH Qatar	132	F	29	140	Screening	PTB	CAT-1	B+	Lost to follow up
1634	SGH Qatar	96	M	19.5	120	Screening	PTB	CAT-1	Clinical	Treatment Complete
1635	SGH Qatar	168	M	33	133	Screening	PTB	CAT-2	Clinical	Treatment Complete
1636	SGH Qatar	72	M	14.5	105	Screening	PTB	CAT-1	Clinical	Treatment Complete
1637	SGH Qatar	84	M	15.5	100	Screening	PTB	CAT-1	Clinical	Treatment Complete
1638	SGH Qatar	84	F	14	112	Screening	PTB	CAT-1	Clinical	Treatment Complete
1639	SGH Qatar	168	M	24	130	Screening	PTB	CAT-1	Clinical	Treatment Complete

1640	SGH Qatar	108	F	19.4	115	Screening	PTB	CAT-1	Clinical	Treatment Complete
1641	SGH Qatar	84	M	16.7	107	Screening	PTB	CAT-1	B+	Treatment Complete
1642	SGH Qatar	156	M	39	146	Screening	PTB	CAT-1	Clinical	Treatment Complete
1643	SGH Qatar	132	M	21	130	Screening	PTB	CAT-1	Clinical	Treatment Complete
1644	SGH Qatar	84	M	13	105	Screening	PTB	CAT-1	Clinical	Treatment Complete
1645	SGH Qatar	48	M	14.5	101	Screening	PTB	CAT-1	Clinical	Treatment Complete
1646	SGH Qatar	72	M	13.8	100	Screening	PTB	CAT-1	Clinical	Treatment Complete
1647	SGH Qatar	108	M	20	120	Screening	PTB	CAT-1	Clinical	Treatment Complete
1648	SGH Qatar	132	F	35.2	131	Screening	PTB	CAT-1	Clinical	Treatment Complete
1649	SGH Qatar	60	F	14.8	100	Screening	PTB	CAT-1	Clinical	Treatment Complete
1650	SGH Qatar	9	F	4.9	68	Screening	PTB	CAT-1	Clinical	Treatment Complete
1651	SGH Qatar	120	M	30	140	Screening	PTB	CAT-1	Clinical	Treatment Complete
1652	SGH Qatar	168	F	39.5	152	Screening	PTB	CAT-1	B+	Treatment Complete
1653	SGH Qatar	168	F	18	121	Screening	PTB	CAT-1	Clinical	Treatment Complete
1654	SGH Qatar	48	M	14	95	Transfer In	PTB	CAT-1	Clinical	Transfer out
1655	SGH Qatar	156	F	23	131	Screening	PTB	CAT-1	Clinical	Treatment Complete
1656	SGH Qatar	132	F	13.6	105	Screening	PTB	CAT-1	Clinical	Treatment Complete
1657	SGH Qatar	60	F	12	97	Screening	PTB	CAT-1	Clinical	Treatment Complete
1658	SGH Qatar	96	M	18	123	Screening	PTB	CAT-1	Clinical	Treatment Complete
1659	SGH Qatar	156	F	6	69	Screening	PTB	CAT-1	Clinical	Treatment Complete
1660	SGH Qatar	48	M	12.5	94	Screening	PTB	CAT-1	Clinical	Treatment Complete
1661	SGH Qatar	60	F	11.1	100	Screening	PTB	CAT-1	Clinical	Treatment Complete
1662	SGH Qatar	156	M	28	135	Screening	PTB	CAT-1	Clinical	Treatment Complete
1663	SGH Qatar	96	F	18.5	130	Screening	PTB	CAT-1	Clinical	Treatment Complete
1664	SGH Qatar	24	F	7.5	70	Screening	PTB	CAT-1	Clinical	Treatment Complete
1665	SGH Qatar	96	M	15.9	100	Screening	PTB	CAT-1	Clinical	failure
1666	SGH Qatar	132	F	26	130	Screening	PTB	CAT-1	Clinical	Treatment Complete
1667	SGH Qatar	24	M	9	76	Screening	PTB	CAT-1	Clinical	Treatment Complete
1668	SGH Qatar	60	F	11.7	86	Screening	PTB	CAT-1	Clinical	Treatment Complete
1669	SGH Qatar	24	M	9.5	72	Screening	PTB	CAT-1	Clinical	Treatment Complete
1670	SGH Qatar	96	M	19.8	120	Screening	PTB	CAT-1	Clinical	Treatment Complete
1671	SGH Qatar	108	F	19.5	121	Screening	PTB	CAT-1	Clinical	Treatment Complete
1672	SGH Qatar	132	F	23.1	144	Screening	EPTB	CAT-1	B+	Treatment Complete
1673	SGH Qatar	144	M	28	136	Screening	PTB	CAT-1	Clinical	Treatment Complete
1674	SGH Qatar	120	F	19.6	126	Screening	PTB	CAT-1	Clinical	Treatment Complete
1675	SGH Qatar	96	M	13.4	100	Screening	PTB	CAT-1	Clinical	Treatment Complete
1676	SGH Qatar	48	M	10.6	87	Screening	PTB	CAT-1	Clinical	Treatment Complete
1677	SGH Qatar	48	F	13.9	96	Screening	PTB	CAT-1	Clinical	Treatment Complete
1678	SGH Qatar	84	M	17	114	Screening	PTB	CAT-1	Clinical	Transfer out
1679	SGH Qatar	132	M	19.4	126	Screening	PTB	CAT-1	Clinical	Treatment Complete
1680	SGH Qatar	144	F	20.6	129	Screening	PTB	CAT-1	Clinical	Treatment Complete

1681	SGH Qatar	96	M	20.5	120	Screening	EPTB	CAT-1	Clinical	Treatment Complete
1682	SGH Qatar	72	M	13.3	100	Screening	PTB	CAT-1	Clinical	Treatment Complete
1683	SGH Qatar	132	M	22.4	128	Screening	PTB	CAT-1	Clinical	Treatment Complete
1684	SGH Qatar	60	F	13.5	106	Screening	EPTB	CAT-1	Clinical	Transfer out
1685	SGH Qatar	96	F	17.3	111	Screening	PTB	CAT-1	Clinical	Treatment Complete
1686	SGH Qatar	60	M	14.5	109	Screening	PTB	CAT-1	Clinical	Lost to follow up
1687	SGH Qatar	96	M	19.5	113	Screening	PTB	CAT-1	Clinical	Treatment Complete
1688	SGH Qatar	60	F	13.5	104	Screening	PTB	CAT-1	Clinical	Treatment Complete
1689	SGH Qatar	12	F	7	70	Screening	PTB	CAT-1	Clinical	Treatment Complete
1690	SGH Qatar	30	F	11	90	Screening	PTB	CAT-1	Clinical	Treatment Complete
1691	SGH Qatar	144	F	28.6	141	Screening	PTB	CAT-1	Clinical	Treatment Complete
1692	SGH Qatar	84	M	16	111	Screening	PTB	CAT-1	Clinical	Treatment Complete
1693	SGH Qatar	96	M	22	122	Screening	PTB	CAT-1	Clinical	Treatment Complete
1694	SGH Qatar	72	M	17.7	116	Screening	PTB	CAT-1	Clinical	Treatment Complete
1695	SGH Qatar	72	F	14.7	95	Screening	PTB	CAT-1	Clinical	Transfer out
1696	SGH Qatar	72	F	20	114	Screening	EPTB	CAT-1	Clinical	Treatment Complete
1697	SGH Qatar	120	F	23	138	Screening	PTB	CAT-1	B+	Treatment Complete
1698	SGH Qatar	84	F	17.4	102	Screening	PTB	CAT-1	Clinical	Treatment Complete
1699	SGH Qatar	144	M	25.7	139	Screening	PTB	CAT-1	Clinical	Treatment Complete
1700	SGH Qatar	84	M	17.2	116	Screening	PTB	CAT-1	Clinical	Treatment Complete
1701	SGH Qatar	72	F	12.2	106	Screening	EPTB	CAT-1	Clinical	Treatment Complete
1702	SGH Qatar	84	M	16	108	Screening	PTB	CAT-1	Clinical	Transfer out
1703	SGH Qatar	96	F	21	121	Screening	PTB	CAT-1	Clinical	Lost to follow up
1704	SGH Qatar	60	F	15	102	Screening	PTB	CAT-1	Clinical	Treatment Complete
1705	SGH Qatar	108	M	14.8	107	Screening	PTB	CAT-1	Clinical	Treatment Complete
1706	SGH Qatar	96	F	13.8	103	Screening	PTB	CAT-1	Clinical	Treatment Complete
1707	SGH Qatar	120	F	22.7	134	Screening	PTB	CAT-1	Clinical	Treatment Complete
1708	SGH Qatar	84	F	15.8	108	Screening	PTB	CAT-1	Clinical	Lost to follow up
1709	SGH Qatar	120	F	24.3	132	Screening	PTB	CAT-1	Clinical	Lost to follow up
